# Single cell spatial profiling of the matrisome identifies region-specific adhesion and signaling networks in glioblastoma

**DOI:** 10.1038/s42003-025-09270-7

**Published:** 2025-12-09

**Authors:** Arpan De, Santiago A. Forero, Ali Pirani, John E. Morales, Marisol De La Fuente-Granada, Sumod Sebastian, Jason T. Huse, Leomar Y. Ballester, Jeffrey S. Weinberg, Frederick F. Lang, Kadir C. Akdemir, Joseph H. McCarty

**Affiliations:** 1 Department of Neurosurgery, The University of Texas M. D. Anderson Cancer Center, Houston, TX USA; 2 Department of Pathology, The University of Texas M. D. Anderson Cancer Center, Houston, TX USA

**Keywords:** CNS cancer, Cancer microenvironment

## Abstract

The human brain contains a milieu of extracellular matrix (ECM) components that promote normal development and physiology. ECM signaling pathways are often dysregulated in brain pathologies including the malignant cancer glioblastoma (GBM). Here, we used single-cell spatial transcriptomic platforms to map the expression of nearly 400 ECM genes in matching non-cancerous brain and GBM samples. At least four different GBM cell populations have been identified that show unique ECM expression profiles and spatial enrichment in distinct intratumor regions. Spatial mapping demonstrates largely non-overlapping expression signatures of ECM components in GBM stromal cell types, particularly in vascular endothelial cells and microglia/macrophages. Comparisons of GBM versus lower grade astrocytoma samples identifies differential expression of key ECM components. Computational analysis reveals novel ECM ligand-receptor networks between GBM and stromal cells. This spatial atlas provides new insights into ECM control of brain tumor initiation and progression and identifies potential targets for therapy in GBM.

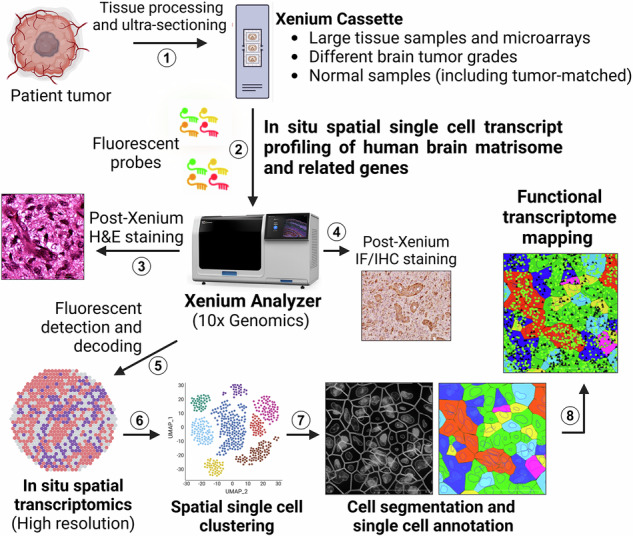

## Abstract (web summary)

Using spatial transcriptomics, this study profiled the expression of hundreds of matrisome genes at single-cell resolution in human brain tumors, revealing targetable adhesion and signalling components in glioblastoma and lower-grade gliomas.

## Introduction

Glioblastoma (GBM) is a primary brain cancer with rapidly proliferative and highly invasive growth features^1,2^. Despite current treatments including surgery and chemoradiation, GBM typically recurs, resulting in a survival rate of about 12–15 months for most patients^3^. Stromal cells in the GBM microenvironment, including reactive astrocytes, microglia and macrophages, and vascular endothelial cells, play key roles in promoting tumor growth and recurrence^4,5^. Prior reports using single-cell RNA-seq, proteomics, and/or spatial transcriptomic approaches have profiled the diverse stromal cell populations in GBM^6,7^. GBM cells and their stromal counterparts also produce hundreds of extracellular matrix (ECM) factors that are a major component of the tumor stroma^8^. ECM adhesion and signaling events are coupled to growth and progression of GBM; for example, stem-like cancer cells localize to ECM-rich vascular niches where they likely exploit local cues for growth and survival benefits^9^. As GBM cells proliferate and infiltrate the brain parenchyma they remodel ECM components in vascular basement membranes to locally activate growth factor and cytokine signaling events, leading to pathological angiogenesis and edema due to the breakdown of the blood-brain barrier^10^. Invasive GBM cells also deposit new ECM factors in the interstitial matrices around neurons and perivascular spaces to form classic “secondary structures of Scherer”^11^.

The matrisome is the repertoire of ECM genes, proteins and ECM-associated factors found in tissues and organisms^12^. It includes core matrisome components such as collagens, proteoglycans, and glycoproteins that provide structural support, as well as ECM-affiliated proteins like growth factors, cytokines, and enzymes that interact with or modify the ECM. The matrisome plays crucial roles in cell adhesion, migration, differentiation, and tissue organization, and its composition varies between different tissues and developmental stages. In addition to providing structural integrity to tissues, the matrisome critically regulates key cellular behaviors, including adhesion, migration, proliferation, differentiation, and signal transduction. There are more than 1000 genes that encode ECM proteins and related components that comprise the human matrisome^13^. In the highly heterogeneous GBM microenvironment, where malignant cells exhibit transcriptional diversity and interact with stromal components, the matrisome likely serves to mediate pro-tumorigenic communication networks. However, our understanding of which specific matrisome genes drive GBM’s notorious invasiveness and therapeutic resistance remains surprisingly limited. In situ spatial transcriptomic platforms can directly detect and quantify RNA transcripts at the single-cell levels within their native tissue environment^14^, making this technology highly suitable for large-scale profiling of matrisome gene expression in intact tumor samples.

Here, we have used Xenium-based single-cell spatial transcriptomics to profile the in situ expression of nearly 400 matrisome genes in human brain tumors, including grade II and III astrocytoma as well as multiple GBM samples. These efforts reveal a heterogeneous pattern of ECM gene expression, and show differential enrichment of many matrisome genes in cancer cells and stromal cell types in select spatial regions in GBM. Computational analyses identify several ECM ligand-receptor pairings that mediate adhesion and communication between cancer cells and stromal cells in the GBM microenvironment. Collectively, these efforts uncover new ECM factors in GBM as well as their specific cells of origin, which may lead to potential therapeutic interventions to improve survival of patients with GBM.

## Results

### Spatial single-cell transcriptomic profiling identifies distinct tumor regions and stromal cell populations in GBM

**Fig. 1:**
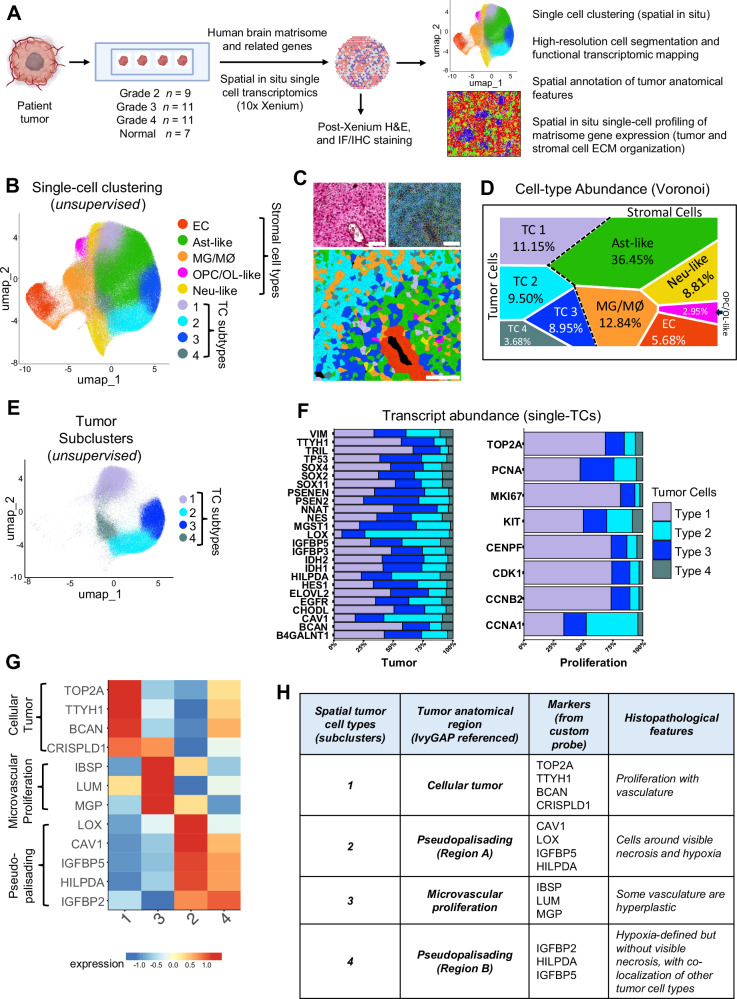
In situ single-cell analysis of matrisome gene expression in GBM. **A** Experimental design showing patient samples and strategies for in situ spatial profiling efforts. A total of 38 patient samples (differentially graded brain tumor and normal) were profiled in this study. A custom probe panel was designed to detect expression of core matrisome, ECM-related, tumor-enriched, and cell-type markers (*n* = 478) of the human brain. This figure was created and modified with BioRender.com. **B** Uniform manifold approximation and projection (UMAP) visualization representing unsupervised single-cell fine clustering of tumor and stromal cell types annotated from a human GBM sample (*n* = 1). Pseudo-colored clusters are defined by differential expression profiles for cell-type-specific and related matrisome markers. **C** Representative region from a GBM sample showing H&E-stained (left, upper panel) and high-resolution cell segmentation images (right, upper panel). Bottom panel shows a magnified image of the region with single cell-types assigned pseudo-colors based on UMAP annotations (**B**). Scale bars: 100 µm. **D** Voronoi diagram showing abundances of tumor and stromal cell-type clusters constituting the GBM sample (*n *= 1). Quantitation of relative percentages based on the total number of cells (*n* = 321,020). **E** Subset UMAP showing the four TC subclusters. **F** Stacked bar plot showing single-cell transcript abundance for TC enriched genes (left) and cell proliferation markers (right) for the four subtypes identified in the GBM sample (**E**). **G** Expression heatmap showing top DEGs in TC subtypes used to annotate GBM anatomic features. Marker genes selected from the Ivy Glioblastoma Atlas Project (GAP) database. Normalized expression values were averaged by tumor subtype, log-transformed and scaled. **H** Summary of the histopathological features identified for each anatomic region in the GBM tissue annotated by the enrichment of select markers detected in the matrisome (**G**).

**Fig. 2:**
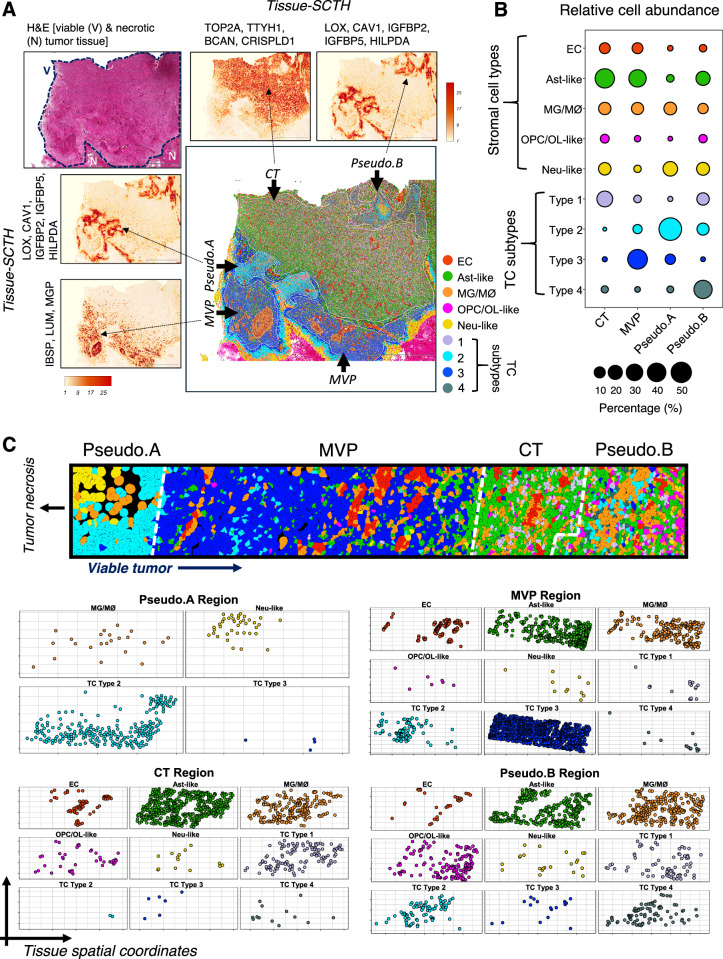
Spatial organization of TCs and stromal cell types in GBM. **A** Representative H&E-stained region of the GBM sample (*n* = 1) showing viable (V) and necrotic (N) tumor tissue (top left). Only viable tissue (encircled by dotted line) was analyzed. Spatial cumulative transcriptomic heatmap (SCTH) showing localization and co-enrichment of select markers used for annotating anatomical regions in the GBM tissue. Cellular tumor (CT), microvascular proliferation (MVP), and pseudopalisading (Pseudo.A and Pseudo.B) regions are indicated in the image showing pseudo-colored TC and stromal cell types. Scale bars: 2000 µm. **B** Dot plot representing relative abundance of TC and stromal cell-types from each anatomical region profiled across a representative GBM tissue subsection (*n* = 1 tumor sample). Dot size corresponds to the percentage of TC subtypes and stromal cell-types per region. Total number of cells profiled per region; CT: 37,192, MVP: 8978, Pseudo.A: 4632, and Pseudo.B: 13,893. **C** Representative localization of TC and stromal cell types highlighting spatial organization and abundance. Single-cells are denoted by cell-type-specific pseudo-colored solid circles across tissue coordinates within segregated GBM anatomical niches. The regions (Pseudo.A, MVP, CT and Pseudo.B) are indicated by white dashed lines (top). Two-dimensional layered representation of spatial cellular organization in each GBM anatomical landscape (bottom). Cell coordinates were extracted using the 10X Genomics Xenium Explorer software.

Single-cell spatial mRNA profiling was performed using multiple human astrocytoma sections of differing grades as well as matching non-cancerous human brain samples (Fig. 1A and Supplementary Table 1). Custom-designed DNA probes recognizing 478 gene products encoding core ECM factors (*n* = 148), ECM-affiliated factors (*n* = 167), integrins (*n* = 23), and other selected markers enriched in tumor and/or brain stromal cell types (*n* = 140) were used to profile gene expression (Supplementary Table 2). Unsupervised single-cell clustering by uniform manifold approximation and projection (UMAP) analyses identified multiple tumor and stromal cell types in GBM (Fig. 1B-D). Detailed analysis of the tumor cell (TC) population identified four distinct subclusters based on differential expression profiles of matrisome markers, tumor-enriched markers, and cell proliferation markers (Figs. 1E, F and 2B). Available RNA-seq data from the Ivy Glioblastoma Atlas Project (GAP)^15^ were cross-referenced to compare gene expression profiles across four GBM histopathological regions to select gene signatures exhibiting pattern enrichment (Fig. 1G, H). Analysis of spatial single-cell transcriptional enrichment profiles in TC subtypes uncovered four anatomically and spatially distinct histopathologic regions within the GBM sample (Figs. 1G, H and 2A). TC subtypes 1 and 3 were more diffusely positioned throughout the tumor in regions annotated as cellular tumor (CT) and microvascular proliferation (MVP), respectively (Figs. 1H and 2A). In contrast, TC subtypes 2 and 4 were more enriched near pseudopalisading regions Pseudo.A and Pseudo.B, respectively.

**Fig. 3:**
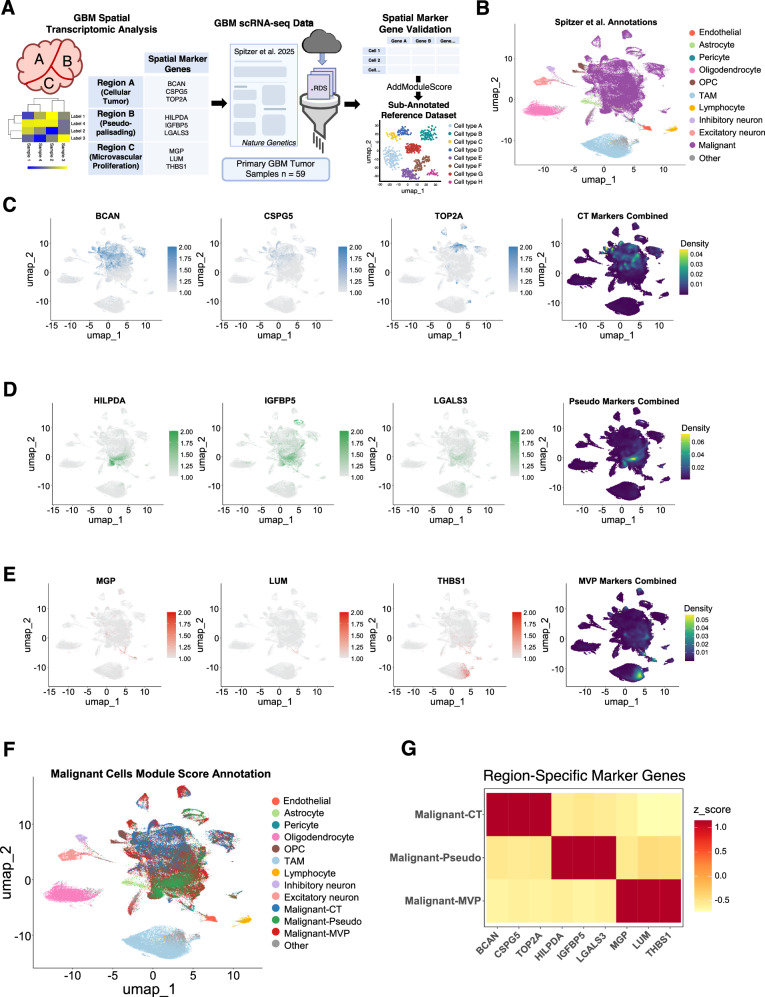
Validation of tumor subregion marker genes in independent scRNA-seq datasets. **A** Graphical depiction of workflow for validation of spatially enriched marker genes. Spatial marker genes for three tumor subregions Cellular tumor (CT), Pseudopalisading (Pseudo), and Microvascular proliferation (MVP) were annotated from our Xenium spatial transcriptomics analysis. A publicly available scRNA-seq GBM dataset was downloaded and subset to match our primary GBM tissue samples. The resulting subset scRNA-seq dataset included 59 samples. The AddModuleScore function from the Seurat workflow was implemented to sub-annotate malignant subregions using spatially enriched marker genes. This figure was created and modified with BioRender.com. **B** UMAP of Spitzer et al. scRNA-seq dataset, reproduced using the author’s original cell-type annotations. **C** FeaturePlots showing spatially enriched marker genes (BCAN, CSPG5, TOP2A) for the Cellular tumor (CT) subregion. Density plot shows combined expression of the three genes. **D** FeaturePlots showing spatially enriched marker genes (HILPDA, IGFBP5, LGALS3) for the Pseudopalisading tumor (Pseudo) subregion. Density plot shows combined expression of the three genes. **E** FeaturePlots showing spatially enriched marker genes (MGP, LUM, THBS1) for the Microvascular proliferation tumor (MVP) subregion. Density plot shows combined expression of the three genes. **F** UMAP of Spitzer et al. data showing malignant tumor cells sub-annotated based on module score of spatially enriched marker genes. **G** Z-score normalized heatmap of averaged scRNA-seq cell segregating into tumor subregions based on the expression of spatially enriched marker genes annotated from our Xenium data.

We next analyzed the spatial distribution of TC and stromal cell types by sampling a tissue area comprising 64,695 single cells within one GBM sample containing all four anatomic regions (Fig. 2A). The proportion of cell types and their corresponding in situ spatial organization showed distinctive patterns of localization (Fig. 2B, C). To validate the robustness and reproducibility of matrisome gene spatial expression patterns, we queried a recently published and publicly available dataset in which 59 different primary GBM samples were profiled by single-cell RNA sequencing (scRNA-seq)^16^. This study also distinguished subpopulations of malignant cells from non-malignant stromal cells based on variations in chromosomal copy number. The AddModuleScore function from the Seurat workflow was implemented to sub-annotate TCs and stromal cell sub-types in the CT, MVP and Pseudo subregions from this independent dataset (Fig. 3A, B). Using three spatially-enriched marker genes for each tumor subregion (CT: BCAN, CSPG5, TOP2A; Pseudo: HILPDA, IGFBP5, LGALS3; and MVP: MGP, LUM, THBS1) we identified distinct sub-populations of malignant cells from the scRNA-seq data (Fig. 3C-E). Comparisons of matrisome gene expression in different populations of TCs and stromal cells from the 59 GBM samples validated the presence of distinct tumor subregions (Fig. 3F, G).

To analyze tumor grade-specific differences in matrisome gene expression, we performed parallel spatial transcript mapping of a grade III astrocytoma sample (IDH1 R132H). Spatial transcript profiling and unsupervised single cell clustering identified diverse TC and stromal cell types in the grade III astrocytoma (Supplementary Fig. 1A, B). Cluster analysis revealed the presence of four TC sub-clusters based on differential gene expression (Supplementary Fig. 1A–D). Several differentially expressed genes (DEGs) linked to cell proliferation, e.g. MKI67 and PCNA, were enriched in TC clusters 1 and 4 in comparison to clusters 2 and 3 (Supplementary Fig. 1D). Several DEGs belonging to different ECM categories of the matrisome were more enriched in TC cluster 4 (Supplementary Fig. 1E). Analysis of spatial positioning of the four different TC clusters revealed largely uniform distribution across the grade III astrocytoma sample (Supplementary Fig. 1F, G).

### Region-specific ECM transcriptomic patterns across cancer cell and stromal cell types

**Fig. 4:**
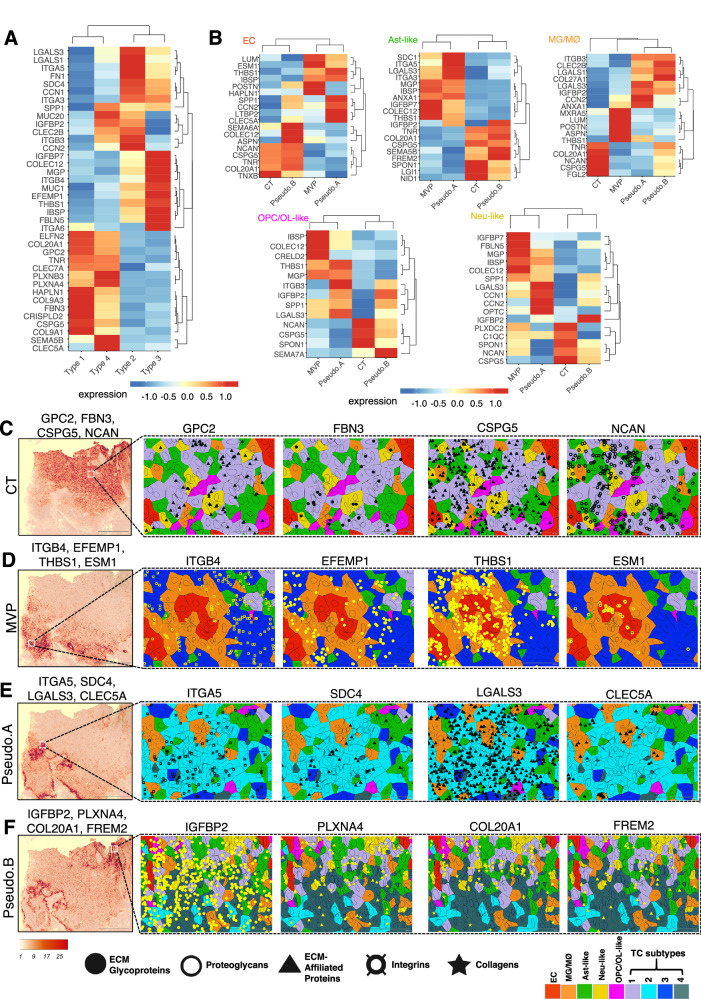
Matrisome gene expression profiles in TCs and stromal cells reveal region specificity. **A** Heatmap showing top DEGs in TC subtypes in different tumor regions from the GBM sample (*n* = 1). TC subtypes residing in different anatomic regions reveal enrichment of unique ECM signatures. Normalized expression values were averaged by tumor subtypes, log-transformed and scaled. **B** Expression heatmaps showing top DEGs in single stromal cells within the four annotated GBM regions (CT, MVP, Pseudo.A, Pseudo.B). Analysis was performed separately for each stromal cell type. Normalized expression values were averaged by region, log-transformed and scaled. **C–F** Spatial in situ transcript localization showing expression patterns of select ECM genes (from **A** and **B**) at single-cell resolution in various GBM regions. Magnified images reveal distinct localization of these ECM signatures in TC subtypes and nearby stromal cell types in regions of interest, shown as boxed areas within SCTHs for select gene groups indicated for CT (**C**), MVP (**D**), Pseudo.A (**E**) and Pseudo.B (**F**) landscapes in the GBM tissue. Transcript shape signifies gene group category (see legend in figure). Scale bars: 2000 µm and 50 µm.

TCs and stromal cells in the four anatomic regions (CT, MVP, Pseudo.A, and Pseudo.B) of the GBM sample were next analyzed. Subtype 4 TCs populating the hypoxic Pseudo.B region revealed comparable gene expression profiles with subtype 1 TCs, localized in close spatial proximity to the CT region (Fig. 4A). Subtype 1 and 4 TCs showed comparable enrichment of ECM genes ELFN2, COL20A1, GPC2, TNR and CLEC7A (Fig. 4A). In contrast, transcripts for HAPLN1, COL9A1, COL9A3, FBN3 and CSPG5 were more abundant in subtype 1 TCs within the CT region. PLXNA4, PLXNB3, SEMA5B, and CLEC5A transcripts displayed elevated expression in the Pseudo.B region comprising subtype 4 TCs (Fig. 4A). Similarly, subtype 2 TCs populating the Pseudo.A region were located near necrotic borders and within the hypoxic microenvironment of MVP region populated by subtype 3 TCs (Figs. 2A and 4A). While certain ECM transcripts, namely IGFBP7, COLEC12, MGP, ITGB4, THBS1, IBSP and FBLN5, showed higher expression in subtype 3 TCs in the MVP region, transcripts for LGALS1, LGALS3, ITGA5 and FN1 were abundant in Pseudo.A region containing subtype 2 TCs (Fig. 4A).

Comprehensive analysis of stromal populations identified microglia (MG), vascular endothelial cells (ECs), neural cells (Neu), oligodendrocyte progenitor cells (OPCs), oligodendrocytes (OLs), and astrocytes in both GBM and grade III astrocytoma samples (Fig. 1B-D and Supplementary Fig. 1). Given that the higher-grade astrocytomas are derived from transformed astroglia/neural stem cells of origin, we will refer to the non-cancerous tumor-infiltrating astrocyte population as “astrocyte-like” (Ast-like). This population likely contains a mixture of reactive non-malignant astrocytes and TCs that express astrocyte markers. Furthermore, the microglial population likely contains macrophages infiltrating from the circulation that express common markers and thus will be referred to as “microglia/macrophages” (MG/MØs). We detected diverse ECM gene expression profiles in the different GBM stromal cell types (Figs. 2 and 4B). ECs and Ast-like cells in both CT and Pseudo.B regions showed comparable expression profiles for some ECM genes (Fig. 4B). In contrast, ECs within the hypoxic Pseudo.B region showed enhanced expression for the ECM genes SEMA6A, COLEC12 and ASPN, while IGFBP2, SEMA5B and FREM2 exhibited higher transcript localization in Ast-like cells (Fig. 4B). Similarly, ECs within the MVP region showed higher abundance of ECM genes LUM, ESM1, POSTN and HAPLN1 whereas those residing in the Pseudo.A region displayed transcript enrichment for SPP1, CCN2, LTBP2 and CLEC5A (Fig. 4B). The Pseudo.A region showed enrichment for SDC1, LGALS3, ITGA3 and ITGA5 transcripts. Tumor-infiltrating MG/MØs reveal distinct ECM expression profiles in the CT and MVP regions (Fig. 4B). MG/MØs of the MVP region were enriched for some ECM genes, namely LUM, POSTN and MXRA5, whereas TNR, COL20A1, CSPG5, NCAN and FGL2 transcripts displayed higher expression in MG/MØs in the CT region.

OPC/OL-like cells in the CT region showed increased expression of NCAN, CSPG5, and SPON1 transcripts whereas those in the MVP region had higher expression of IBSP, COLEC12 and CRELD2 (Fig. 4B). Higher transcript abundance of ITGB3 and LGALS3 were observed in OPC/OL-like cells in the Pseudo.A region in comparison to SEMA7A which displayed increased expression in Pseudo.B region. Some ECM genes were selectively enriched in Neu-like cells residing in the annotated GBM anatomical regions (Fig. 4B). TC subtypes and the stromal cell types revealed distinctly unique spatial expression patterns. For example, GPC2 and FBN3 were enriched in subtype 1 TCs in the CT region in comparison to CSPG5 and NCAN that revealed higher expression in the surrounding stromal cells in addition to the TCs (Fig. 4C). In the MVP region, ITGB4 and EFEMP1 transcripts were more abundant in subtype 3 TCs whereas ESM1 and THBS1 were more localized in proliferating microvascular ECs and perivascular MG/MØs (Fig. 4D). In the hypoxic Pseudo.A region, ITGA5 and SDC4 showed enhanced abundance in subtype 2 TCs in comparison to CLEC5A which was enriched in tumor infiltrating MG/MØs, and LGALS3 that was overall higher in both TCs and surrounding stromal cell types (Fig. 4E). In the Pseudo.B region, IGFBP2 transcripts were found in subtype 4 TCs but other ECM genes like PLXNA4, COL20A1 and FREM2 were elevated in stromal cell types surrounding the necrotic areas (Fig. 4F). Analysis of an independent scRNA-seq dataset replicated spatial patterns of select matrisome genes; for example, NCAN which was expressed in TC1 cells, Ast-like, and Neu-like cells in our GBM sample also showed enriched expression in sub-annotated malignant tumor cells, astrocytes, and neural clusters (Supplementary Fig. 2A-C). We next analyzed expression of a set of genes enriched in GBM stromal cell types (EC, Ast-like, MG/MØ) within each tumor subregion (CT, Pseudo, and MVP). As expected, FeaturePlots of gene expression from the scRNA-seq dataset confirmed our spatial profiling results. Within the various stromal cell clusters, we observed a distinct subpopulation of cells showing enrichment for each tumor subregion-specific DEG (Supplementary Fig. 2D).

We next compared DEGs in the various stromal cell types in grade III astrocytoma versus GBM. The ten most DEGs encoding ECM glycoproteins were enriched in vascular ECs and in MG/MØs whereas overall expression levels of collagen genes were higher in Neu and OPC/OL cells (Supplementary Fig. 3A). While the top ten differentially expressed integrin and proteoglycan genes were distributed between multiple cell types, ECM-affiliated genes showed highest enrichment in ECs followed by MG/MØs and Neu cells (Supplementary Fig. 3A). There were several transcripts showing elevated expression in GBM stromal cell types versus stromal cell types in the grade III tumor, including IGFBP2 (ECM glycoprotein), ANXA1 (ECM-affiliated), ITGA7 (integrin), and SRGN (proteoglycan). Interestingly, we detected upregulation of specific matrisome factors such as MMRN1, SEMA7A, ITGA2, and ESM1 uniquely in GBM vascular ECs (Supplementary Fig. 3B, C).

FeaturePlots confirmed upregulation of select ECM glycoprotein genes, including IGFBP2 and MGP, in the GBM sample versus grade III astrocytoma (Supplementary Fig. 4A-C). Spatial analysis of IGFBP2 and MGP transcript expression revealed upregulated expression in TCs, ECs, and Ast-like populations surrounding blood vessels in GBM (Supplementary Fig. 4C). In comparison to vascular EC-specific expression in the grade III tumor, there was markedly elevated FN1 mRNA abundance in ECs and perivascular cells in the GBM sample (Supplementary Fig. 4D-F). The integrin genes ITGA5 and ITGA7 showed significantly enhanced transcript counts in the GBM tissue compared to the grade III tumor sample (Supplementary Fig. 5A, B). For example, elevated spatial expressions of ITGA5 transcripts were detected primarily in tumor ECs, whereas ITGA7 transcripts were expressed mainly in TCs and Ast-like cells (Supplementary Fig. 5B).

Among the 19 collagen gene transcripts that were profiled, COL16A1, COL8A1, and COL2A1 exhibited higher levels in the GBM tissue versus the grade III astrocytoma sample (Supplementary Fig. 5C, D). There was increased spatial distribution of COL16A1 transcripts primarily in vascular ECs and MG/MØs, with lower expression in surrounding TCs and Ast-like populations in the GBM sample (Supplementary Fig. 5D). Similarly, unlike in grade III astrocytoma, COL8A1 transcripts showed enhanced expression primarily in perivascular MG/MØs in GBM. We also detected increased COL8A1 transcript expression in GBM versus grade III astrocytoma via FeaturePlot visualization of normalized gene expression (Supplementary Fig. 5D). Quantitative profiling of 25 genes encoding proteoglycans revealed that specific transcripts, including LUM and SRGN, exhibit significantly higher counts in the viable region of the GBM sample (Supplementary Fig. 5E, F). We detected enhanced spatial distribution of LUM primarily in GBM ECs and SRGN transcripts mainly in MG/MØs (Supplementary Fig. 5F).

The ECM-affiliated genes ANXA1 and ANXA2 showed robust spatial enrichment in GBM compared to the grade III tumor (Supplementary Fig. 5G, H). There was markedly enhanced expression of ANXA1 and ANXA2 transcripts primarily in GBM cells residing in close juxtaposition with vascular ECs (Supplementary Fig. 5H). In comparison to the grade III sample, ECM-affiliated genes (COLEC12, SDC2 and SEMA5A) showed enhanced spatial expression in GBM regions with perivascular microgliosis associated with pseudopalisading necrosis, that are largely absent in lower-grade gliomas (Supplementary Fig. 6A-C). Additionally, in comparison to the surrounding Ast-like cell populations, the ECM-affiliated gene LGALS3 revealed significantly elevated transcript localization in the pseudopalisading TCs near necrosis (Supplementary Fig. 6D, E). As expected, these four ECM-affiliated genes (COLEC12, SDC2, SEMA5A and LGALS3) did not reveal obvious changes in spatial distribution of cellular transcripts in the grade III astrocytoma sample (Supp. Fig. 6F). We detected lower levels of transcripts for some matrisome genes in GBM versus grade III astrocytoma (Supplementary Fig. 6G-J).

### GBM subregions reveal distinct spatial coherence patterns of matrisome gene expression in blood vessels and perivascular MG/MØs

**Fig. 5:**
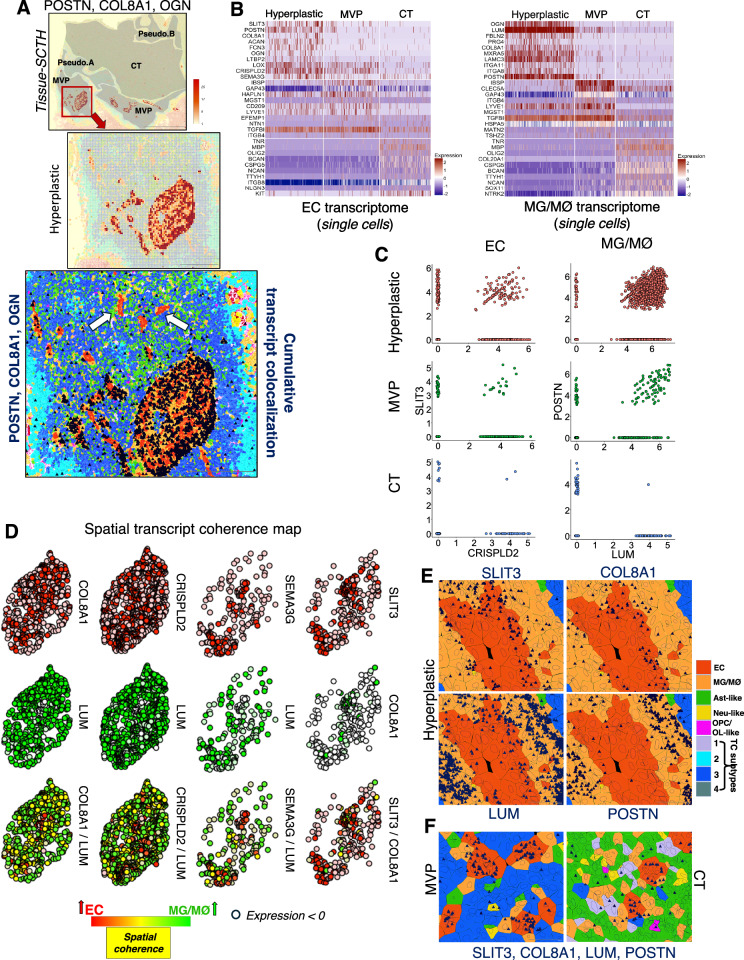
Heterogeneous matrisome gene expression in GBM blood vessels and perivascular MG/MØs. **A** SCTH of the GBM sample (*n* = 1) showing spatial enrichment in hyperplastic blood vessels in the MVP region (top). Magnified image (middle) shows a hyperplastic blood vessel with glomeruloid features in the boxed area (top). The bottom panel shows spatial co-localization of POSTN, COL8A1 and OGN transcripts (dark blue) in ECs and perivascular MG/MØs populating the hyperplastic blood vessels. In comparison, non-hyperplastic blood vessels residing nearby (white arrows) do not show spatial enrichment of POSTN, COL8A1, and OGN transcripts. TC and stromal cell types are shown in pseudo-colors indicated in Figs. 1 and 2. Scale bars: 2000 µm and 500 µm. **B** Single-cell transcriptomic heatmaps showing top DEGs in ECs (left) and MG/MØs (right) residing in hyperplastic blood vessels (2628 cells) as well as non-hyperplastic blood vessels in MVP (1852 cells) and CT (2226 cells) regions. **C** Feature scatter plots showing the correlative expression of select genes from (**B**). **D** Representative coherence maps showing “spatial spots” for EC-expressed (red) and MG/MØ-expressed (green) transcripts for select genes revealing increased correlations in hyperplastic vascular regions in GBM. Enhanced spatial coherence is denoted by yellow “spatial spots” (bottom) for each gene set. **E**, **F** Spatial localization of transcripts (dark blue) for SLIT3, COL8A1, LUM and POSTN in ECs and MG/MØs localized in hyperplastic blood vessels (**E**), and blood vessels located in the MVP and CT regions (**F**). Scale bars: 50 µm.

Next, histologically enriched markers from the Ivy-GAP dataset were used to annotate “hyperplastic” subregions within the GBM MVP area (Fig. 5A). These subregions contained perivascular MG/MØs showing enrichment for POSTN, COL8A1 and OGN transcripts in comparison to the adjacent non-hyperplastic vasculature within the MVP region (arrows in Fig. 5A). Single-cell transcript profiling for ECs and MG/MØs from multiple regions of interest for hyperplastic, MVP (non-hyperplastic) and CT regions revealed enrichment of diverse ECM genes (Fig. 5B). Several DEGs in ECs and MG/MØs were selected for further analysis. ECM glycoproteins SLIT3 and CRSIPLD2 showed co-expression in ECs in hyperplastic blood vessels in comparison to ECs in the non-hyperplastic MVP and CT vasculature (Fig. 5C). The ECM-affiliated gene SEMA3G in ECs displayed spatial coherence with LUM in MG/MØs (Fig. 5D). Also, COL8A1 and CRISPLD2 in ECs showed spatial coherence with LUM in MG/MØs in hyperplastic perivascular niche (Fig. 5D). In comparison to tumor vasculature in MVP and CT regions, SLIT3, COL8A1, LUM and POSTN showed enhanced spatial enrichment within the GBM hyperplastic region (Fig. 5E, F).

### Cell-cell communication networks across GBM histopathological regions

**Fig. 6:**
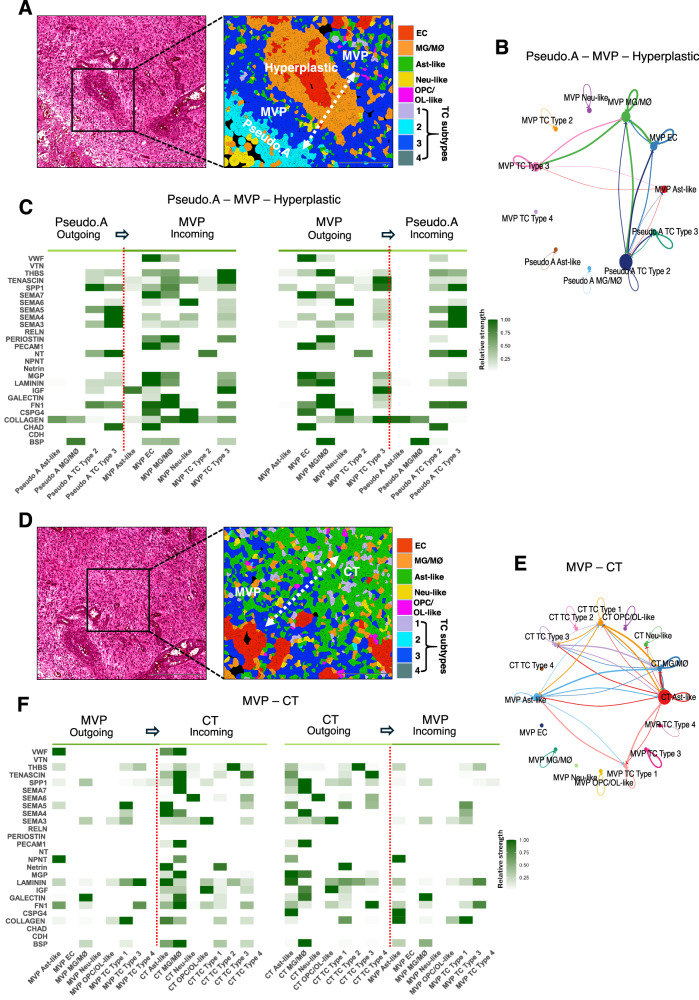
Cell–cell communication networks across GBM anatomic regions. **A** H&E-stained image showing a Pseudo.A-MVP boundary including hyperplastic vasculature (boxed region) in the GBM sample (*n*-=1) (left). Magnified image (right) of the boxed area shown in the H&E-stained image (left) is high-resolution spatial annotation of GBM regions (Pseudo.A, MVP and Hyperplastic) in the GBM sample and UMAP-based spatially segmented pseudo-colored (indicated) cell types. Dashed arrow signifies cellular crosstalk across “boundaries” spatially segregating the annotated GBM regions. Scale bars: 500 µm and 200 µm. **B** Chord plot summarizing interactions between specific cell types across Pseudo.A-MVP regions that include hyperplastic blood vessels. Each node (circle) represents a cell type, with its size equating to overall communication strength. Edges (lines) between nodes represent ligand-receptor interactions with the directionality of interaction indicated by arrows, pointing from the signaling (ligand-expressing) cell to the receiving (receptor-expressing) cell. Notably, MG/MØ and ECs within the MVP region show stronger interactions with sub-type 2 TCs in the Pseudo A region. **C** Heatmap of signaling networks showing outgoing and incoming signaling strength across the Pseudo A-MVP/Hyperplastic region. Color intensity indicates strength of ligand-receptor interactions within each pathway, stronger signals shown in darker green shades. Left panel shows outgoing signals from Pseudo A cell types (light green) to recipient MVP region cell types (dark green). The directionality of outgoing to incoming signaling interactions are displayed above each panel. Right panel shows outgoing signals from MVP region cell types (dark green) to recipient Pseudo A cell types (light green). **D** H&E-stained image showing MVP-CT boundary (boxed area) in the GBM tissue (left). Magnified image (right) of the boxed area shown in the H&E-stained image (left) represents high-resolution spatial annotation of GBM regions (MVP and CT) and UMAP-based pseudo-colored cell types. Dashed arrow signifies representative cellular crosstalk across “boundary” spatially segregating the annotated GBM niches indicated. Scale bars: 500 µm and 200 µm. **E** Chord plot summarizing interactions between region-specific cells across the MVP-CT boundaries. Excluding TC sub-types 2 and 4, cell types in the CT region engage in bidirectional crosstalk with varying strengths. **F** Heatmap of signaling networks showing outgoing and incoming signaling strength across MVP-CT region. Left panel shows outgoing signals from MVP cell types (dark green) to recipient CT region cell types (light green). Right panel shows outgoing signals from CT region cell types (light green) to recipient MVP cell types (dark green).

The CellChat tool^17^ was used to study matrisome interaction networks via known ligand-receptor pairs and their associated signaling pathways. TC subtype 2 located in the Pseudo.A regions showed robust interactions with EC and MG/MØ populations localized in the MVP region primarily through SPP1 and FN1 signaling networks (Fig. 6A-C). The SPP1 network revealed strong outgoing and incoming signaling patterns from TC subtypes 2 and 3 in the Pseudo.A region to EC and MG/MØ populations residing in the adjacent MVP region, respectively. This observation is consistent with SPP1’s role in promoting tumor proliferation, cell migration, and immunomodulation within the perivascular niche^18^. FN1-mediated interactions showed significant bi-directional communication across these cell types, suggesting ECM-driven adhesion and structural organization at the Pseudo.A-MVP boundary, consistent with FN1’s involvement in matrix remodeling and tumor-stroma integration (Fig. 6C). The MVP-CT boundary in the GBM sample also revealed strong cellular interaction patterns (Fig. 6D, E). Ast-like and subtype 3 TCs communicated with adjacent MG/MØ populations across the MVP-CT boundary regions via the FN1 signaling network (Fig. 6E, F). Ast-like cell populations in CT and MVP regions strongly interacted through the laminin signaling network, which was also crucial in mediating cell-cell communication patterns between subtype 3 TCs in the MVP region, and closely localized Ast-like and subtype 1 TCs localized in the CT region (Fig. 6E, F). TCs in the CT region strongly communicated with closely juxtaposed perivascular Ast-like cells via collagens. This highlights the role of ECM scaffolds in regulating cell adhesion, positioning, and local invasion in vascularized regions. In contrast, CT–Pseudo.B regions showed weaker ECM-driven interactions suggestive of a more migratory tumor cell phenotype (Supplementary Fig. 8C, D).

**Fig. 7:**
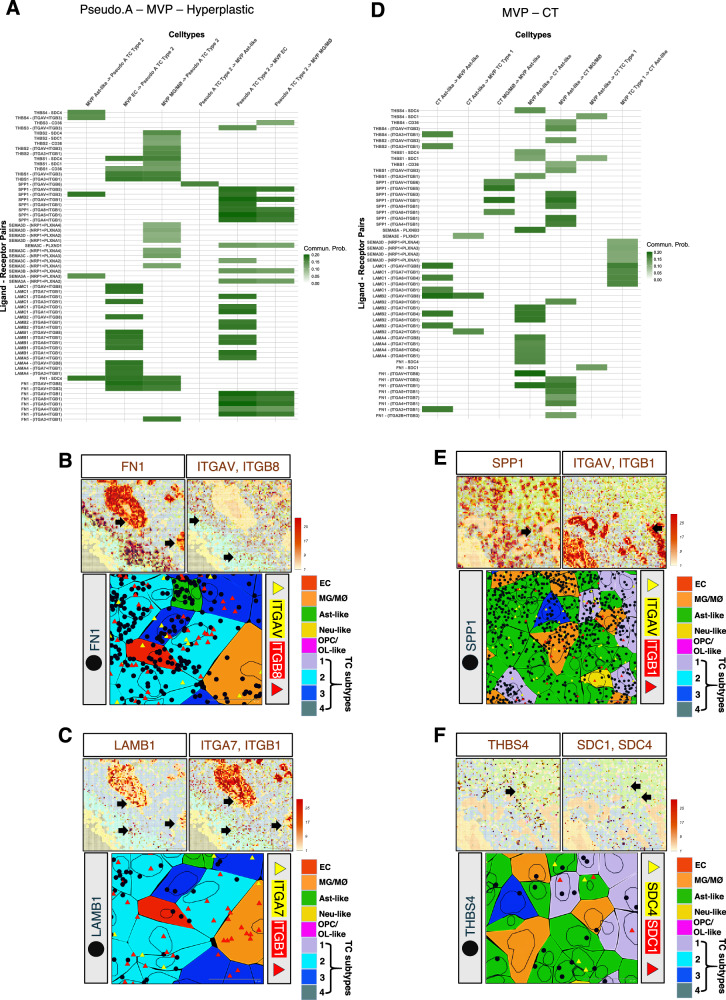
ECM ligand–receptor pairings in different cellular zones in GBM. **A** Heatmap showing communication probabilities for top ligand-receptor pairs enriched in TC and stromal cell types localized across the Pseudo A–MVP–Hyperplastic boundary in the GBM sample (*n* = 1). **B** Top panel shows representative transcript heatmaps for FN1 (ligand) and corresponding integrin receptors (ITGAV and ITGB8) across Pseudo.A-MVP-Hyperplastic boundaries. Bottom panel shows high-resolution image displaying spatial localization of transcripts (dark blue circles for FN1 and multi-colored triangles for integrins) in pseudo-colored cell types. Scale bars: 200 µm and 20 µm. **C** Top panel shows transcript heatmaps for LAMB1 (ligand) and corresponding integrin receptors (ITGA7 and ITGB1) across Pseudo A–MVP–Hyperplastic boundaries. Bottom panel shows representative high-resolution image displaying spatial localization of the transcripts (dark blue solid circle for LAMB1 and multi-colored triangles for integrins) in UMAP-based cell types (pseudo-colors indicated). Scale bars: 200 µm and 20 µm. **D** Heatmap showing communication probabilities for top ligand-receptor pairs enriched in TC and stromal cell types localized across the MVP–CT boundary. **E** Top panel shows transcriptomic heatmaps for SPP1 (ligand) and corresponding integrin receptors (ITGAV and ITGB1) across the MVP–CT boundary. Bottom panel shows representative high-resolution image displaying spatial localization of the transcripts (dark blue solid circle for SPP1 and multi-colored triangles for integrins) in UMAP-based cell types (pseudo-colors indicated). Scale bars: 200 µm and 20 µm. **F** Top panel shows transcriptomic heatmaps for THBS4 (ligand) and corresponding syndecan receptors (SDC1 and SDC4) across the MVP-CT boundary. Bottom panel shows a high-resolution image of transcript spatial localization (dark blue solid circle for THBS4 and multi-colored triangles for syndecans) in pseudocolored cells. Scale bars: 200 µm and 20 µm.

Next, ligand-receptor pairs involved in intercellular signaling pathways were identified for “within” and “boundary” regions across different GBM regions (Fig. 7 and Supplementary Figs. 7, 8). The most notable ligand-receptor pairs were in the Pseudo.A-MVP-Hyperplastic and MVP-CT GBM anatomic regions (Fig. 7A, D). FN1 (ligand) enriched in/around proliferating ECs, and perivascular MG/MØs in the MVP region interacted with integrin receptors (ITGAV and ITGB8) on subtype 2 TCs localized in the neighboring Pseudo.A region (Fig. 7A, B). Bi-directional communication patterns were observed across the subtype 2 TCs (Pseudo.A region) and ECs (MVP region) via LAMB1 (ligand) and integrin receptors (ITGA7 and ITGB1) (Fig. 7A, C). Similar bi-directional interactions were detected across Ast-like cells (MVP region) and MG/MØ populations (CT region) through SPP1 (ligand) and corresponding integrin receptors (ITGAV and ITGB1) localized in both cell types (Fig. 7D, E). Additionally, Ast-like cells (MVP region) revealing enrichment of THBS4 (ligand) communicate with syndecan receptors (SDC1 and SDC4) localized in subtype 1 TCs and Ast-like cells respectively, residing in the neighboring GBM CT region (Fig. 7D, F). Specifically, this demonstrates that perivascular MVP niches act as signaling hubs of ECM-mediated communication coordinating proliferation, ECM deposition, and immune modulation providing ECM framework for GBM growth and invasion. The spatially resolved signaling maps generated by CellChat thus provide a framework for identifying region-specific vulnerabilities and blocking these pathways could disrupt the structural and signaling framework that facilitate tumor growth, invasion, and immune evasion.

### ECM remodeling during tumor growth and infiltration

**Fig. 8:**
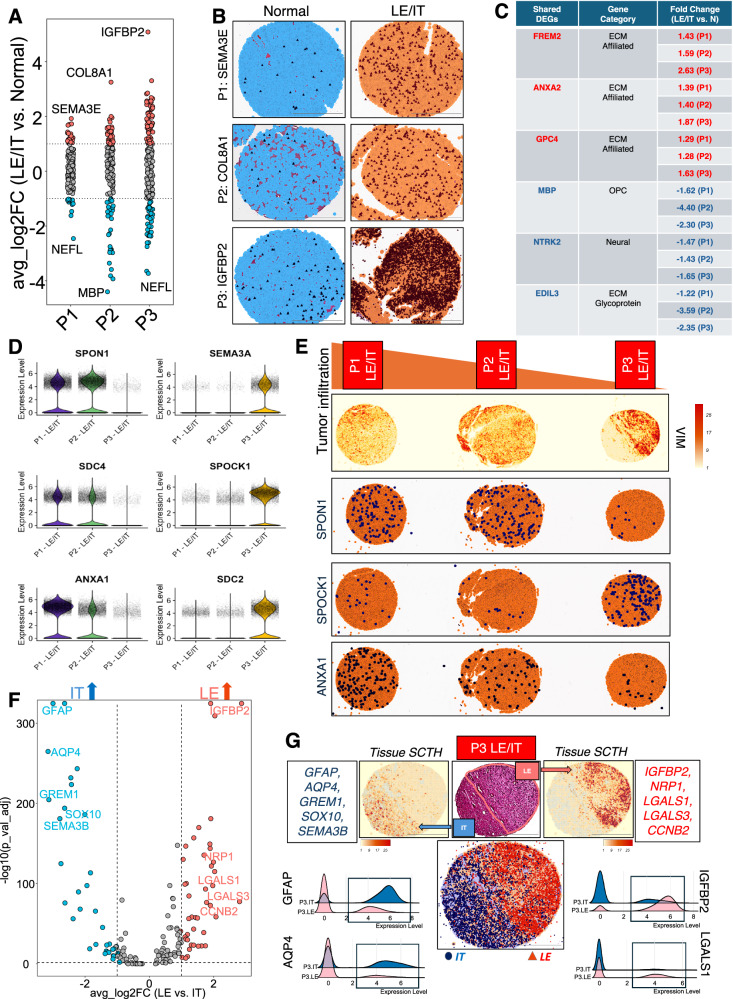
Spatial profiling of matrisome gene expression in GBM infiltrating regions. **A** Strip chart showing differentially expressed matrisome genes (upregulated: red and downregulated: blue) in GBM-LE/IT regions compared to matched non-cancerous brain tissue (*n* = 3 patients). **B** Representative overall spatial expression of top DEGs upregulated in GBM-LE/IT regions compared to the matched normal samples for individual patients (*n* = 3). Transcripts shown in dark blue (normal) and dark purple (LE/IT) localized in single cells (light blue: normal and light orange: LE/IT). Scale bars: 500 µm. **C** Table showing shared upregulated and downregulated DEGs across all three patient GBM-LE/IT samples compared to matched non-cancerous brain tissue. Expression fold-change values in LE/IT samples for shared DEGs for each patient. **D**, **E** Violin plots showing expression of DEGs selected from comparative analysis of GBM-LE/IT patient samples based on degree of infiltration (**D**). Representative VIM spatial transcriptomic heatmaps define varying degrees of infiltration across LE/IT samples from three GBM patients (**E**). Representative images of spatial transcript localization (dark blue) for select genes in single cells (orange) for LE/IT samples across three GBM patients (**E**). Scale bars: 1000 µm. **F**, **G** Volcano plot showing upregulated DEGs (blue: IT and red: LE) in more distinct GBM-LE and GBM-IT regions for samples from P3 (**F**). Representative tissue SCTHs showing elevated compartmentalized expression of select DEGs for adjacent IT (left) and LE (right) regions in the same GBM sample from P3 (**G**). H&E-stained image and spatial image representing cumulative transcript localization of select DEGs for IT (blue) and LE (red) in the GBM sample from P3 are shown in the middle. Ridgeline plots show elevated expression of astroglial markers GFAP and AQP4 in IT region (left) and GBM hallmarks (IGFBP2 and LGALS1) in the LE region (right). Scale bars: 500 µm.

The spatial expression patterns for matrisome and related genes in patient-matched samples from non-cancerous brain and GBM were next analyzed (*n* = 3 of each). The GBM samples contained the tumor leading edge (LE) that included cancer cells infiltrating into brain parenchyma (IT). The annotated LE and IT regions were validated by comparing the expression of gene signatures selected from the Ivy GAP dataset^15^. In comparison to the matching non-cancerous brain samples, ECM genes SEMA3E, COL8A1 and IGFBP2 were spatially elevated in the LE/IT regions in the GBM samples (Fig. 8A, B). Some DEGs like FREM2, ANXA2 and GPC4 revealed common enrichment in LE/IT tumor regions across GBM patient samples compared to the overall downregulation of the OPC/OL marker MBP, the neural marker NTRK2, and the ECM glycoprotein EDIL3 (Fig. 8C). While ECM genes SPON1, SDC4 and ANXA1 were elevated in more infiltrating tumor tissue (patients 1 and 2), SEMA3A, SPOCK1 and SDC2 displayed enhanced expression in less infiltrating GBM sample from patient 3 (Fig. 8D, E). Similarly, immunofluorescence labeling showed robust upregulation of annexin-A1 protein in LE/IT regions (*n* = 2) from an independent cohort of additional patient-derived GBM samples (*n* = 3), further confirming the spatial transcriptomic enrichment of ANXA1 in invasive and infiltrative regions of aggressive brain tumors (Supplementary Fig. 14, top panel). The GBM sample from patient 3 exhibited closely positioned LE and IT regions with a distinctively identifiable tumor periphery. Consequently, we performed spatial profiling of these neighboring areas to analyze the differential expression of matrisome genes (Fig. 8F, G). While GFAP, AQP4, GREM1, SOX10 and SEMA3B (selected from top DEGs shown in Fig. 8F) displayed distinct spatial enrichment in the IT region, IGFBP2, NRP1, LGALS1, LGALS3 and CCNB2 (selected from top DEGs shown in Fig. 8F) were elevated in the GBM periphery (Fig. 8G). In comparison to the LE region, more single cells profiled from the IT region revealed higher expression of both GFAP and AQP4, indicating increased reactive astrogliosis in normal brain areas infiltrated by GBM cells (Fig. 8G). Elevated expression for transcripts IGFBP2 and LGALS1 in cells at the tumor periphery (GBM-LE region) indicate aberrant ECM remodeling at the invasive GBM edge (Fig. 8G).

Matrisome gene expression was profiled using a tumor microarray containing punch biopsy samples from grade II diffuse glioma (*n* = 9), grade III astrocytoma (*n* = 10), and grade IV/GBM (*n* = 7) along with non-cancerous brain tissues (*n* = 4) which included some matching samples. All grade II (G.2) and grade III (G.3) samples harbored R132H mutations in the IDH1 gene, whereas the GBM samples (G.4) were wild type for IDH1. Pseudo-bulk sampling identified variance-based distribution of G.2 and G.3 samples when compared to G.4/GBM (Supplementary Fig. 9A). The majority of the matrisome genes showing significantly elevated transcript abundance in GBM samples belonged to the ECM glycoprotein and ECM-affiliated gene categories, including ANXA2 (Supplementary Fig. 9B). Immunofluorescence labeling revealed robust upregulation of annexin-a2 protein expression in GBM versus normal brain as well as lower grade glioma samples (Supplementary Fig. 9C). We next performed independent pairwise sample-based comparative transcriptomic analysis for matched non-cancerous and differentially graded brain tumor tissues (Supplementary Fig. 10). The top three DEGs in tumor versus matched normal tissues were identified for each matrisome gene group. In comparison to the matched normal samples, the top three DEGs encoding ECM glycoproteins (SMOC1, ABI3BP and SPARC) showed the highest transcript abundance followed by ECM-affiliated DEGs (PLXNB3, SEMA6A and PLXNB1) in grade 2 diffuse glioma samples (Supplementary Fig. 9A). All three DEGs encoding proteoglycans (HAPLN2, ACAN and HAPLN1) showed higher transcript enrichment in the grade 2 gliomas in comparison to the matched normal samples. Interestingly, the only differentially expressed collagen gene (COL24A1) showed decreased transcript abundance in perivascular cells of the grade 2 glioma sample (Supplementary Fig. 9A). In comparison to normal tissue, spatial enrichment for SMOC1, ACAN, and SEMA6A transcripts was evident in cells of grade 2 glioma samples. Comparison of the grade 3 astrocytoma to the matched normal sample, revealed the top three DEGs encoding ECM glycoproteins (NTN1, AEBP1 and SPARC) and ECM-affiliated factors (C1QA, LGALS9 and C1QL1) (Supplementary Fig. 9B). The top three proteoglycan DEGs (SPOCK1, SPOCK3 and HAPLN2) displayed reduced transcript enrichment in single cells of the grade 3 sample when compared to the matched normal sample. Similarly, two differentially expressed collagen genes (COL9A2 and COL16A1) exhibited negative fold-changes in transcript counts for the grade 3 tumor sample (Supplementary Fig. 9B). Also shown are DEG analysis from additional matched grade4/GBM versus normal (Supplementary Fig. 9C) and corresponding variance-based single-cell principal component analysis plots for matched grade 2 diffuse glioma versus normal, grade 3 astrocytoma versus normal, and grade4/GBM versus normal samples from the tumor microarray (Supplementary Fig. 9A-C).

**Fig. 9:**
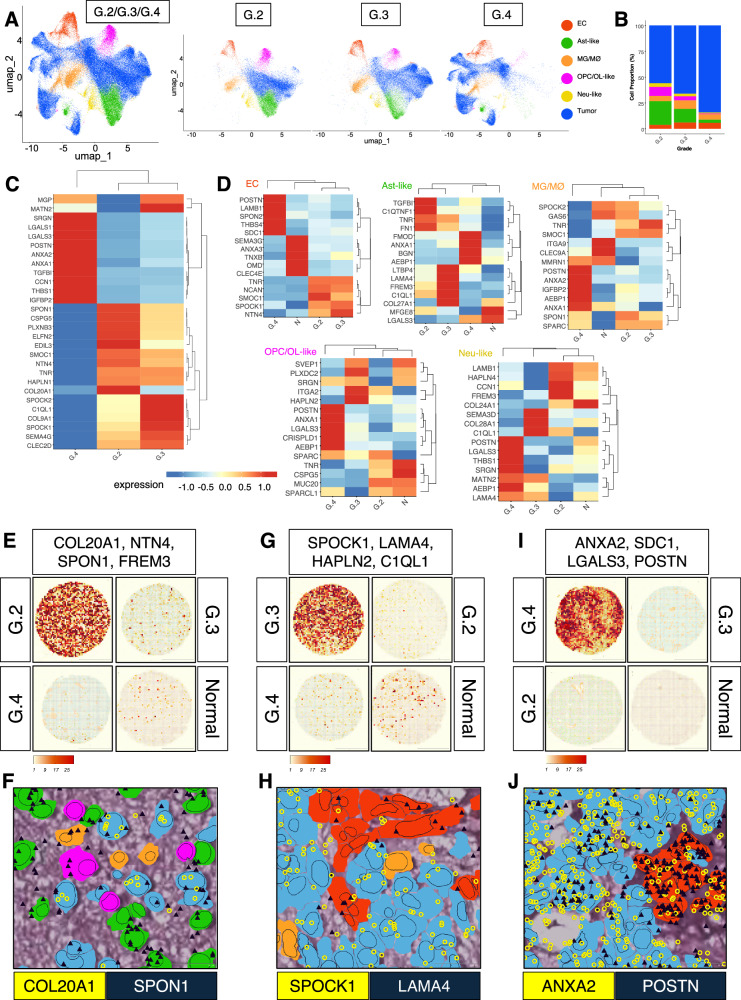
Cross-grade spatial transcriptomic analysis of tumor and stromal cell types. **A** UMAP representing unsupervised single-cell combined (left) and grade-separated (right) clustering of tumor and stromal cell types from grade II diffuse glioma (G.2, *n* = 9), grade III astrocytoma (G.3, *n* = 10), and grade IV/GBM (G.4, *n* = 7) samples. Pseudo-colored clusters defined by differential expression profiles for related matrisome markers. **B** Stacked bar plot showing comparison of TC and stromal cell-type abundances in tumor samples from different grades (**A**). Cell-types shown in the plots correspond to assigned pseudo-colors from UMAPs (**A**). **C** Expression heatmap of TCs showing top DEGs across the multiple brain tumor samples from different grades. Expression profiles for tumor cells from G.2 diffuse glioma and G.3 astrocytoma samples show some overlap compared to GBM samples, which reveal more selective enrichment. Normalized expression values were averaged by tumor grade, log-transformed and scaled. **D** Heatmaps of stromal cell-types showing DEGs across the different glioma grades (G.2, G.3, and G.4) in comparison to non-cancerous (*N*) samples (*n* = 4). Normalized expression values were averaged by sample, log-transformed and scaled. **E**, **F** Representative tissue SCTHs from G.2, G.3, G.4 and non-cancerous samples showing DEGs in G.2 in comparison to G.3, G.4 and normal samples (**E**). DEGs were selected from single-cell transcriptomic heatmaps for TC and stromal cell-types (**C**, **D**) to compare expression levels for matrisome and related gene signatures across brain tumor grades. Representative high-resolution image shows spatial enrichment of COL20A1 transcripts (yellow) in TCs in comparison to higher localization of SPON1 transcripts (dark blue) in surrounding Ast-like cells in G.2 sample. Cells are assigned UMAP-based pseudo-colors shown in (**A**). Scale bars: 500 µm and 20 µm. **G**, **H** SCTHs from G.2, G.3, G.4 and normal samples showing enhanced co-enrichment of DEGs in G.3 in comparison to G.2, G.4 and normal samples (**G**). DEGs selected from single-cell transcriptomic heatmaps for TC and stromal cell-types shown in (**C**, **D**) to represent and compare expression levels for matrisome and related gene signatures across tumor grades. High-resolution image showing spatial enrichment of SPOCK1 transcripts (yellow) in TCs in comparison to higher localization of LAMA4 transcripts (dark blue) in ECs in the G.3 sample. Cells are assigned UMAP-based pseudo-colors shown in (**A**). Scale bars: 500 µm and 20 µm. **I**, **J** SCTHs from G.2, G.3, G.4 and normal samples showing enhanced co-enrichment of DEGs in G.4 in comparison to G.2, G.3 and normal samples (**E**). DEGs were selected from single-cell transcriptomic heatmaps for TC and stromal cell-types shown in (**C**) and (**D**) to represent and compare expression levels for matrisome and related genes. Representative high-resolution image shows concomitant spatial enrichment of both ANXA2 (yellow) and POSTN (dark blue) transcripts in tumor and ECs in G.4 sample. Cells are assigned UMAP-based pseudo-colors shown in (**A**). Scale bars: 500 µm and 20 µm.

Differential gene expression analysis identified variable profiles for each tumor grade when compared to normal samples (Supplementary Fig. 10A-C). Glioma grade-based and integrative single-cell clustering identified diverse enrichment of multiple stromal cell types based on differential gene expression profiles (Fig. 9A, B and Supplementary Fig. 11). Specific ECM gene sets were spatially enriched in TC and stromal cell types for each glioma grade (Fig. 9E-J). For example, COL20A1 and SPOCK1 were elevated in TCs for grade II and 3 astrocytoma samples respectively (Fig. 9C, D, F, H). SPON1 was more diffusely upregulated in grade II tumor and infiltrating multiple stromal cell types, while LAMA4 was enriched in tumor ECs in grade III samples (Fig. 9C, D, F, H). For GBM samples, both ANXA2 and POSTN were significantly enhanced in TCs and ECs (Fig. 9C, D, J).

To further validate the cell type-specific expression of select matrisome genes (Fig. 1G), we analyzed protein expression patterns by immunohistochemistry. Analysis of the CT marker BCAN/brevican, the MVP marker LUM/lumican, and the Pseudo marker IGFBP2/insulin growth factor binding protein 2 confirmed region-specific protein expression (Supplementary Fig. 12A, B). Triple immunofluorescence labeling revealed enrichment of the IGFBP2 protein in GFAP^+^ cells but not in ECs within a GBM region of pseudopalisading necrosis. IGFBP2 protein expression was low or undetectable in lower-grade (G.2 and G.3) astrocytoma samples (Supplementary Fig. 12C, D). In contrast, G.2 and G.3 astrocytoma samples showed elevated expression of NTN4/netrin-4 protein in vascular basement membranes and in some TCs (Supplementary Fig. 13A-C), which confirms the transcript expression data (Fig. 9C-E). We also confirmed differential expression levels of ANXA1/annexin-a1 protein at the GBM leading edge (Supplementary Fig. 14A) that matched the spatial mRNA expression patterns (Fig. 8E). Triple immunofluorescence analysis of additional patient samples revealed elevated levels of ANXA1 protein in GFAP^+^ cells but not in CD31^+^ blood vessels in GBM samples in comparison to G.2 and G.3 astrocytoma samples (Supplementary Fig. 14B). Collectively, these data reveal that matrisome protein expression patterns largely match the differential mRNA expression patterns identified by 10X Xenium analysis.

## Discussion

The ECM represents a critical component of the GBM microenvironment that fundamentally shapes tumor behavior and therapeutic resistance. As the complete set of ECM proteins and ECM-associated factors, the matrisome creates the structural and biochemical scaffold within which GBM cells proliferate and invade. In GBM, the matrisome is profoundly dysregulated compared to normal brain tissue. The healthy brain ECM consists mainly of hyaluronic acid, proteoglycans, and basement membranes around blood vessels and lining the ventricles. In this report, we show that malignant gliomas dramatically remodel this environment, depositing multiple factors that are normally absent or minimal in normal brain tissue. Using high-resolution spatial transcriptomics, we have mapped the expression patterns of nearly 400 human ECM genes at single-cell resolution, revealing important insights into the stromal landscape of malignant brain tumors. By focusing our probe panel on matrisome genes, we have gained new spatial resolution of ECM organization and cell-ECM interactions within the GBM microenvironment. This targeted approach also allows for the detection of lower-abundance matrisome transcripts that might be missed in broader panels, while also reducing data complexity and computational burden for ECM-focused analyses. The probe set also contains nearly 100 cell-type-specific markers, allowing characterization of changes in matrisome expression patterns in different stromal and cancer cell sub-populations. However, the probe panel does not allow for analysis of chromosomal copy number variation that would be more accurately distinguish tumor cells from non-malignant cells. For example, the Ast-like population that is enriched in CT regions of GBM samples is likely a mixture of reactive non-malignant astrocyte and tumor cells. In addition, by using the matrisome probe set we cannot analyze broader transcriptional programs related to metabolic states, stress responses, and/or developmental pathways that could provide important context for understanding how ECM changes integrate with other cellular processes.

Our spatial profiling of the matrisome identifies at least four different tumor cell states in GBM, which largely match with recent single-cell transcriptome sequencing studies^6,16,19–21^. Clusters 2 and 4 in this study localize to hypoxic and necrotic regions, consistent with recent evidence showing hypoxia-dependent ECM remodeling promotes GBM aggressiveness through HIF-1α-dependent mechanisms^22^. While matrisome transcripts like ITGA5 and LGALS3 were enriched in TCs and infiltrating stromal cells residing around necrotic regions, the hypoxic tumor regions revealed enhanced expression of IGFBP2 and COL20A1. The ECM-affiliated genes LGALS1 and LGALS3 showed significant upregulation in GBM, particularly in regions of pseudopalisading necrosis. These galectins have recently been implicated in immune evasion and maintenance of cancer stem cells^23^, suggesting potential roles in GBM progression and/or therapy resistance.

Spatial enrichment of tumor cluster 1 in the well-vascularized CT region, and cluster 3 in the MVP region suggests involvement in cerebral blood vessel co-option, a process increasingly recognized as crucial for GBM progression^9,24^. In comparison to the CT vasculature, ECM glycoproteins IBSP and MGP, both calcium-binding ECM proteins, were upregulated in MVP regions. Studies showing calcium-dependent regulation of GBM invasion^25^ suggest that MGP might influence tumor progression through modulation of local calcium dynamics and/or mechanotransduction pathways. In comparison to angiogenic blood vessels in neighboring CT regions, hyperplastic blood vessels within MVP regions differentially expressed matrisome factors like POSTN, OGN and LUM in perivascular MG/MØs. In contrast, SLIT3 and COL8A1 displayed elevated levels in hyperplastic ECs. Interestingly, SLIT3 is a factor that orchestrates angiogenesis during embryonic development^26^. Our findings regarding the ECM proteoglycans LUM and SRGN build upon emerging evidence that proteoglycans influence the immune microenvironment in GBM^27^. The specific enrichment of these factors in ECs and MG/MØs suggests potential roles in the recently described immunosuppressive vascular niche^25^. This spatial organization may regulate immune cell trafficking and activation.

Recent studies have demonstrated that integrin signaling influences GBM stem cell maintenance through YAP/TAZ-dependent pathways^28^. Our spatial mapping data showing ITGA5 in pseudopalisading TCs and ITGA6/ITGB4 in MVP regions suggests that these signaling events may originate in specific cellular compartments. Our analysis revealed significant upregulation of ANXA1 and ANXA2 in GBM, extending beyond previous TCGA findings by providing crucial spatial contexts^29^. While ANXA1 was enriched in MVP, pseudopalisading regions, and more diffusely infiltrative GBM peripheries, ANXA2 was elevated in TCs and ECs in comparison to lower-grade tumor samples. The enrichment of these calcium-dependent phospholipid-binding proteins in perivascular regions suggests roles in maintaining the perivascular niche, particularly relevant given recent evidence that annexins regulate calcium-dependent exosome release in GBM^30^.

In summary, this work establishes a foundation for several future areas of investigation. First, functional studies are needed to determine how the identified ECM components may cooperatively influence tumor development, progression, and therapy resistance. Second, the integration of our spatial matrisome data with other emerging datasets, including spatial metabolomics^31^ and single-cell chromatin accessibility data^21^, could provide a more complete understanding of the GBM microenvironment. The integration of spatial gene expression analysis into routine clinical practice for GBM patients represents a transformative advancement that could fundamentally reshape tumor diagnosis, classification, and treatment. Like how whole genome sequencing has impacted cancer treatment decisions, spatial transcriptomics offers the ability to map gene expression patterns across tumor tissue while preserving crucial spatial context. This technology enables identification of not only which genes are expressed, but precisely where they are active within the tumor microenvironment, providing insights into complex cellular interactions driving tumor progression and treatment resistance. From a clinical diagnostic perspective, spatial gene expression profiling could enable more sophisticated molecular classification systems beyond current histopathological markers. By analyzing differential expression patterns across distinct tumor regions, clinicians may identify molecular signatures correlating with infiltrative disease extent - critical for GBM prognosis given these tumors’ invasive nature along white matter tracts and vascular structures. Such spatial analysis could reveal heterogeneous expression patterns within individual tumors, identifying high invasive potential regions missed by traditional bulk sequencing or standard histology. This enhanced understanding could directly impact surgical planning, radiation field design, and adjuvant therapy selection, leading to personalized treatment strategies accounting for each tumor’s unique spatial molecular landscape. The therapeutic implications extend particularly to treatments targeting specific ECM components and their cellular sources. Spatial transcriptomics could identify regions where particular ECM components are overexpressed, guide targeted delivery system development, and predict patient response to ECM-targeted therapies based on spatial molecular profiles.

## Materials and methods

### Human tissue samples

All ethical regulations relevant to human research participants were followed. All patient tumor data included in this publication were obtained with appropriate informed consent. The study protocol and consent procedures were reviewed and approved by the Institutional Review Board of The University of Texas MD Anderson Cancer Center. All samples were de-identified for experimental studies. Patient-derived solid tissue samples included grade II diffuse astrocytoma (*n* = 9), grade III astrocytoma (*n* = 11), GBM (*n* = 8), normal (*n* = 4), and tissue matched GBM core and normal (*n *= 3) tissue samples. Additional samples used for spatial gene expression validation studies via immunohistochemical and immunofluorescence techniques included an independent set of grade II diffuse astrocytoma (*n* = 3), grade III astrocytoma (*n* = 3) and GBM (*n* = 5).

### Xenium gene panel design and analysis

Using the MatrisomeDB (https://matrisomedb.org) that comprises “a searchable collection of curated proteomic data on the ECM of healthy and diseased human and murine samples”, we selected genes that belong to human core matrisome and matrisome-affiliated categories and displayed detectable expression in the human brain (https://www.proteinatlas.org). A total of 478 genes were selected to design the probe panel that also included cellular markers relevant to normal human brain functions and brain tumor growth/progression, selected and curated based on single-cell atlas data^32^. Fluorophore-tagged probes were designed by 10x Genomics (Pleasanton, CA). All parameters for Xenium analysis are manufacturer-designed and optimized^16^.

### Cell segmentation

Grade III astrocytoma (*n* = 1), GBM (*n* = 1), and tissue matched normal and GBM samples (*n* = 3) were used for cell segmentation on the Xenium analyzer with Xenium ranger version 1.7.1.0 (10x Genomics, Pleasanton, CA) which utilizes nuclear expansion distance of 15 µm to establish cell boundaries. Grade II glioma (*n* = 9), grade III astrocytoma (*n* = 10), GBM (*n* = 7) and non-cancerous brain tissues (*n* = 4) were used for cell segmentation on the Xenium analyzer with Xenium ranger version 2.0.0.10 (10x Genomics, Pleasanton, CA) which utilizes nuclear expansion distance of 5 µm to establish cell boundaries.

### Downstream analysis

Single cell IDs for regions of interest were exported from the Xenium Explorer software (10x Genomics, Pleasanton, CA) as CSV files and used for downstream analysis. Off-instrument downstream analyses were performed on RStudio 4.4.0. Molecular coordinates and h5 matrix files from each xenium experiment were imported using the Seurat and scCustomize packages, and CSV files containing cell IDs for regions of interest were imported into R using the csv package (Bergsma 2022, 10.32614/CRAN.package.csv). Cluster analyses and differential gene expression were performed using built in functions from the Seurat package. Data integration was performed using the Harmony package^33^.

### Spatial data integration

Grade III astrocytoma and GBM data were integrated in R studio by merging the two data sets into one Seurat object and then performing the Harmony integration method, which projects all cells into a shared embedding, which allows cells to be grouped by cell type rather than sample identity. Cluster analysis was performed by assessing the differential expression of conserved marker genes following the Seurat workflow for integrated single cell data.

### Annotating tumor regions of interest using Ivy GAP

Marker gene RNA-seq data from the IVY Glioblastoma Atlas Project^15^ was used to identify anatomical features in the GBM sample. Using region-specific marker genes present in our Xenium panel, we identified Cellular Tumor (CT), Pseudopalisading (Pseudo.A, Pseudo.B), Microvascular proliferation (MVP), and Hyperplastic MVP regions.

### Cell-type and transcript abundance

The relative abundance of cell types was assessed by taking the proportion of each cell-type for a whole sample, or for a selected region of interest. Proportions were shown as Voronoi plots, stacked barplots, or dotplots of percentages. Transcript abundance was assessed by taking the proportion of transcripts expressed in a region or cluster out of the total transcripts for a gene.

### Differential gene expression

Differential gene expression analyses in GBM, grade III astrocytoma, integrated grade III and GBM data, paired tumor vs. normal comparison, tumor grade comparison, and tissue matched grade III vs. normal (*n* = 1), grade III vs. normal (*n* = 2) and GBM vs. normal (*n* = 1) were performed using the default statistical tests from the FindAllMarkers and FindMarkers functions in the Seurat workflow which identifies differentially expressed genes between groups of cells. A log_2_fold change (log_2_FC) greater than 1 or less than −1 and an adjusted p-value (Bonferroni correction for multiple comparisons) of less than 0.05 were used as cutoffs for statistical significance. Differential gene expression analysis for patient brain tumor grade comparisons (grade II vs. GBM and grade III vs. GBM) was performed following the DESeq2 protocol for pseudo-bulk data (Harvard Chan Bioinformatics Core website).

### Z-score calculations

For grade III astrocytomas, the top 10 differentially expressed genes for glycoproteins, collagens, integrins, proteoglycans, and ECM-affiliated proteins marker genes were filtered for Ast-like, ECs, MG/MØs, OPC/OL-like, and Neu-like cells. Z-scores were calculated by taking the average normalized transcript count of a gene for each cell type or tumor subcluster minus the average normalized transcript count for all cell types or subclusters and dividing the difference by the standard deviation of all cell types or subclusters.

### CellChat cell–cell interactions

CellChat v2.1.0 was used to analyze region-specific cell–cell interactions and identify key ECM-mediated signaling pathways along with their corresponding ligand–receptor pairs. The analysis was conducted in a contact- or spatial distance-dependent manner, utilizing the truncated mean method to compute average gene expression and communication probabilities of signaling pathways.

### Immunohistochemistry and immunofluorescence

Formalin fixed paraffin embedded sections from tumor tissue samples were deparaffinized and heat-induced epitope retrieval was performed using 1x Tris-EDTA buffer (pH 9.0, without detergent) for 30 min at 95 °C. Sections were permeabilized and blocked with 10% donkey serum and 5% BSA in 1x PBS containing 0.25% Triton X-100 and 0.05% Tween-20) at RT for 1 h. Immunohistochemical analysis was performed with mouse anti-fibronectin antibody (1:300, cat#66042-1-Ig, Proteintech). Slides were incubated in ABC Reagent (catalog #PK-4000, Vector Laboratories) for 30 min at RT. ImmPACT DAB Substrate (catalog #SK-4105, Vector Laboratories) was added for signal detection. For immunofluorescence, the following primary antibodies were used: rabbit anti-annexin A1 (1:200, cat#21990-1-AP, Proteintech), rabbit anti-annexin A2 (1:200, cat#11256–1-AP, Proteintech), rabbit anti-IGFBP2 (1:200, cat#ab188200, Abcam), goat anti-netrin 4 (1:200, cat#AF1254-SP, R&D Systems), chicken anti-GFAP (1:2000, cat#NBP1-05198, Novus Biologicals), goat anti-CD31 (1:100, cat#AF3628, R&D Systems), rabbit anti-Iba1 (1:200, cat#013-27691, Fujifilm Wako), rabbit anti-brevican (1:500, cat#NBP2-15616, Novus) and goat anti-lumican (1:500, cat#AF2846, R&D systems). Following washes with PBS-Tween20, the sections were incubated with the following secondary antibodies at RT for 1 hour: Alexa Flour® 594 donkey anti-chicken (1:500, cat#703-585-155, Jackson ImmunoResearch), Alexa Flour® 488 donkey anti-rabbit (1:500, cat#711-545-152, Jackson ImmunoResearch) and Alexa Fluor® 647 donkey anti-goat (1:500, cat#705-605-147, Jackson ImmunoResearch). Secondary antibodies used for immunohistochemistry include horse anti-goat IgG (H + L), biotinylated (1:500, cat#BA-9500, Vector Laboratories Inc.) and horse anti-rabbit IgG (H + L), biotinylated (1:500, cat#BA-1100, Vector Laboratories Inc.).

### Image acquisition and analysis

Immunofluorescence images were acquired using Olympus FLUOVIEW FV3000 confocal laser scanning microscope^34^. Multi-dimensional acquisition was carried out using *z*-stacks with 1.0-3.0 μm slicing intervals at a scan rate of 4 μs/pixel with a resolution of at least 1024×1024 pixels per slice and digitally compiled in FV31S-SW (ver. 2.4.1.198). Image acquisition parameters including laser power, exposure time, voltages and gain were assigned uniformly across all samples for each imaging channel. All images were analyzed using Fiji and Image J software packages^35^ and serial *z*-stack were used for all analysis and image processing using same parameters for all samples. Multiple areas of different tissue regions (*n* ≥ 1) were imaged across all samples. Bright-field images were acquired using a light microscope (Olympus BX43) equipped with 10× and 20× objectives, with identical acquisition parameters maintained across all samples.

### Validation of xenium results with an independent scRNA-seq dataset

Gene expression validation was performed on a publicly available annotated scRNA-seq dataset. Data from Spitzer et al.^16^ was downloaded from Gene Expression Omnibus (GSE274546). From this dataset, primary GBM (*n* = 59) samples were subset and used for validation of our spatial transcriptomics results. Pre-annotated malignant cells from Spitzer et al. data were sub-annotated for tumor subregion using the AddModuleScore function from the Seurat package in R. Malignant cells were scored as Cellular tumor cells (CT), Pseudopalisading (Pseudo), or Microvascular proliferation (MVP) based on their average expression of spatially enriched marker genes for each tumor subregion (CT: BCAN, CSPG5, TOP2A; Pseudo: HILPDA, IGFBP5, LGALS3; MVP: MGP, LUM, THBS1). FeaturePlots of individual genes and density plots of combined gene expression were created to observe patterns of gene expression. A z-score heatmap of averaged CT, Pseudo, and MVP was created to observe the specificity of marker gene expression across these three cell sub-types.

### Statistics and reproducibility

Statistical analyses were conducted in RStudio (v4.4.0). Differential gene expression was assessed by implementing the FindAllMarkers and FindMarkers functions from the Seurat package, which identify differentially expressed genes (DEGs) between cell groups based on the Wilcoxon rank-sum test with Bonferroni correction for multiple comparisons. For pseudo-bulk data, DESeq2 was implemented to perform DEG analysis using a negative binomial generalized linear model and the Wald test, applying the Benjamini-Hochberg correction to control the false discovery rate. Genes with an absolute log_2_ fold-change >1 and an adjusted p-value ≤ 0.05 were considered statically significant. Z-scores were calculated by taking the average normalized transcript count of a gene for each cell type or tumor subcluster minus the average normalized transcript count for all cell types or subclusters and dividing the difference over the standard deviation of all cell types or subclusters. CellChat analyses were performed using CellChat v2.1.0 in R (version 4.4.1). CellChat objects were generated with spatial distance parameters set to ratio = 1 and tol = 1.5 to account for spatial factors. Communication probability estimation was conducted in a contact- or spatial distance-dependent manner using the truncated mean method with the following settings: trim = 0, distance.use = TRUE, interaction.range = 100, scale.distance = 1, contact.dependent = TRUE, and contact.range = 10. Default threshold parameters were applied (thresh = 0.05) to determine significant pathways and ligand–receptor interactions.

### Reporting summary

Further information on research design is available in the Nature Portfolio Reporting Summary linked to this article.

## Supplementary information

Supplementary Information

Reporting Summary

## Data Availability

All spatial transcriptomic data, including raw Xenium output files, processed data, and annotated metadata have been deposited in the EMBL-EBI BioStudies database under accession number S-BSST2273. All other data are available from the corresponding author on reasonable request.

## Code availability

Analysis scripts used in this study are available from the corresponding author upon request. No custom code was developed or used in this study. Data normalization and analyses were performed in RStudio (v4.4.0) using publicly available software packages, including Seurat (v5.3.1), Harmony (v1.2.4), DESeq2 (v1.48.2), and CellChat (v2.1.0).
