# Supplementary material for: Single cell spatial profiling of the matrisome identifies region-specific adhesion and signaling networks in glioblastoma
Source: Commun Biol. 2025 Dec 9;9:17. doi: 10.1038/s42003-025-09270-7 (PMC12769532; doi:10.1038/s42003-025-09270-7)
Supplement: Supplementary file 1 — Supplementary Information [file 42003_2025_9270_MOESM1_ESM.pdf]

Supp. Fig. 1

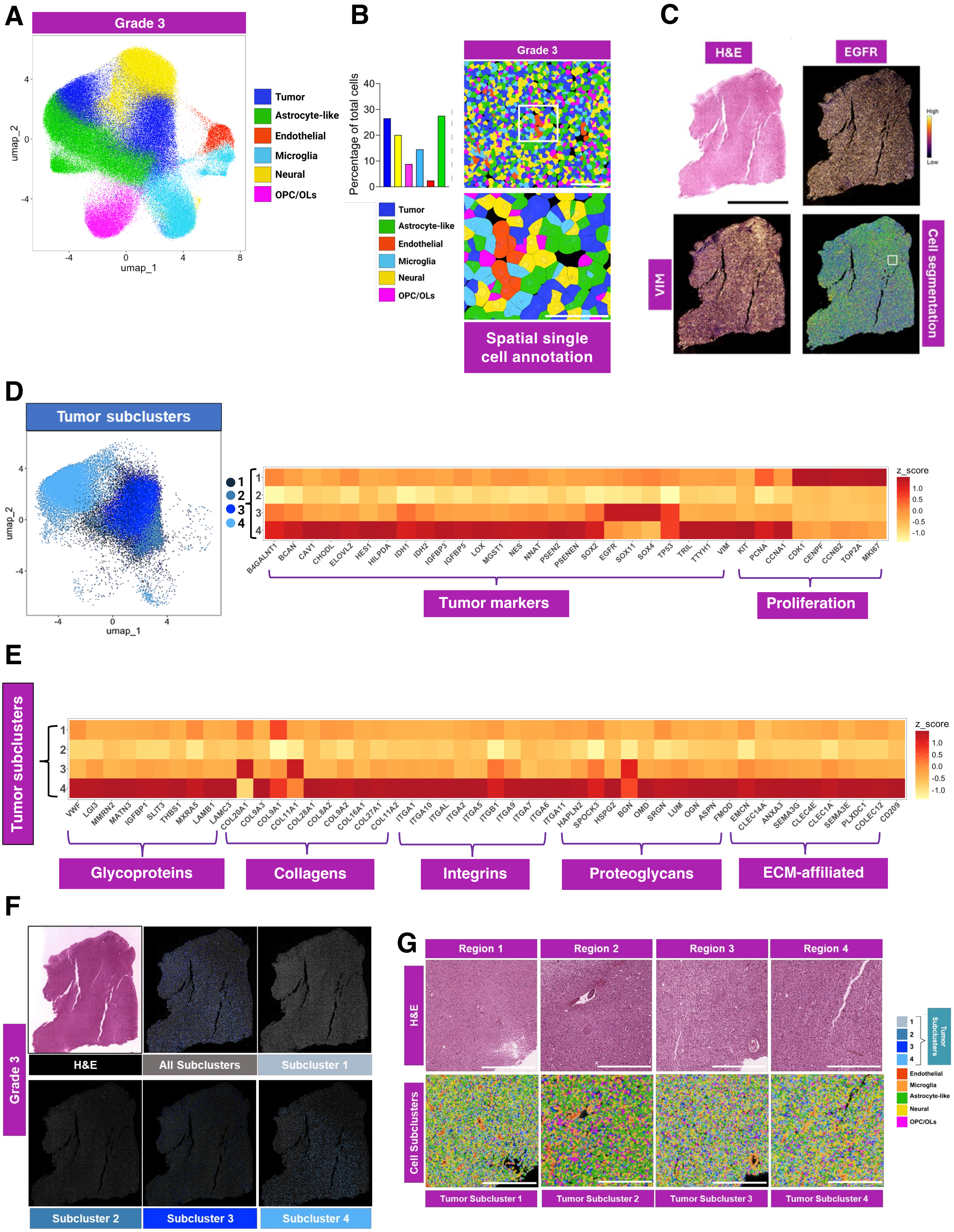

A
Grade 3
umap_2
umap_1
Tumor
Astrocyte-like
Endothelial
Microglia
Neural
OPC/OLs
B
Percentage of total cells
Grade 3
Spatial single cell annotation
C
H&E
EGFR
VIM
Cell segmentation
High
Low
D
Tumor subclusters
z_score
B4GALNT1 BCAN CAV1 CHODL ELOVL2 HES1 HILPDA IDH1 IDH2 IGFBP3 IGFBP5 LOX MGST1 NES NNAT PSEN2 PSENEN SOX2 EGFR SOX11 SOX4 TP53 TRIL TTYH1 VIM KIT PCNA CCNA1 CDK1 CENPF CCNB2 TOP2A MKI67
Tumor markers
Proliferation
E
Tumor subclusters
VWF LGI3 MMRN2 MATN3 IGFBP1 SLIT3 THBS1 MXRA5 LAMB1 LAMC3 COL20A1 COL9A3 COL9A1 COL11A1 COL28A1 COL8A2 COL9A2 COL16A1 COL27A1 COL11A2 ITGA1 ITGA10 ITGAL ITGA2 ITGA5 ITGB1 ITGA9 ITGA7 ITGA6 ITGA11 HAPLN2 SPOCK3 HSPG2 BGN OMD SRGN LUM OGN ASPN FMOD EMCN CLEC14A ANXA3 SEMA3G CLEC4E CLEC1A SEMA3E PLXDC1 COLEC12 CD209
Glycoproteins
Collagens
Integrins
Proteoglycans
ECM-affiliated
F
Grade 3
H&E
All Subclusters
Subcluster 1
Subcluster 2
Subcluster 3
Subcluster 4
G
Region 1
Region 2
Region 3
Region 4
H&E
Cell Subclusters
Tumor Subcluster 1
Tumor Subcluster 2
Tumor Subcluster 3
Tumor Subcluster 4
Tumor Subclusters
Endothelial
Microglia
Astrocyte-like
Neural
OPC/OLs

Supp. Fig. 2

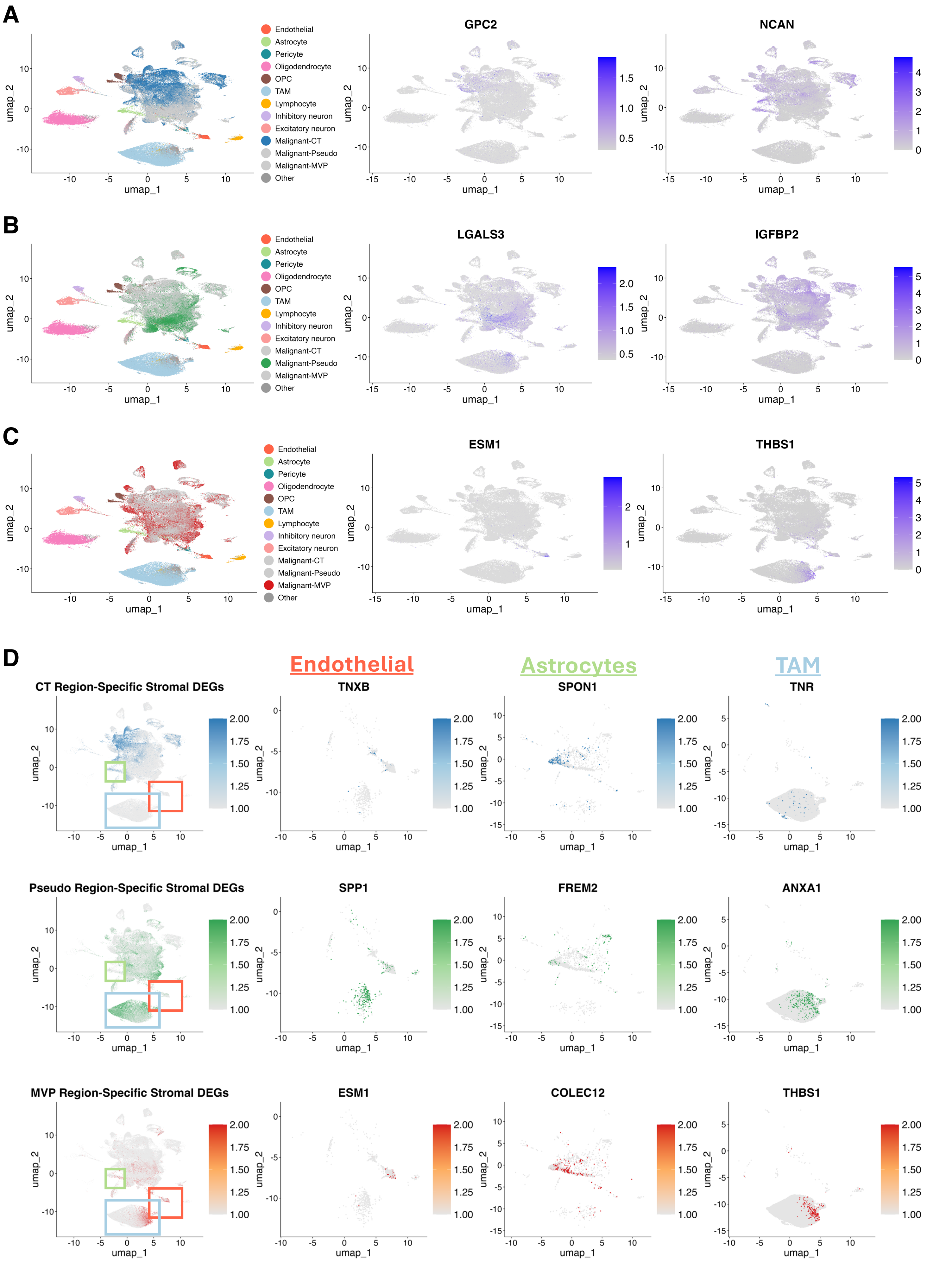
A
Endothelial
Astrocyte
Pericyte
Oligodendrocyte
OPC
TAM
Lymphocyte
Inhibitory neuron
Excitatory neuron
Malignant-CT
Malignant-Pseudo
Malignant-MVP
Other
GPC2
NCAN
umap_1
umap_2
B
LGALS3
IGFBP2
C
ESM1
THBS1
D
Endothelial
Astrocytes
TAM
CT Region-Specific Stromal DEGs
TNXB
SPON1
TNR
Pseudo Region-Specific Stromal DEGs
SPP1
FREM2
ANXA1
MVP Region-Specific Stromal DEGs
ESM1
COLEC12
THBS1

# Supp. Fig. 3

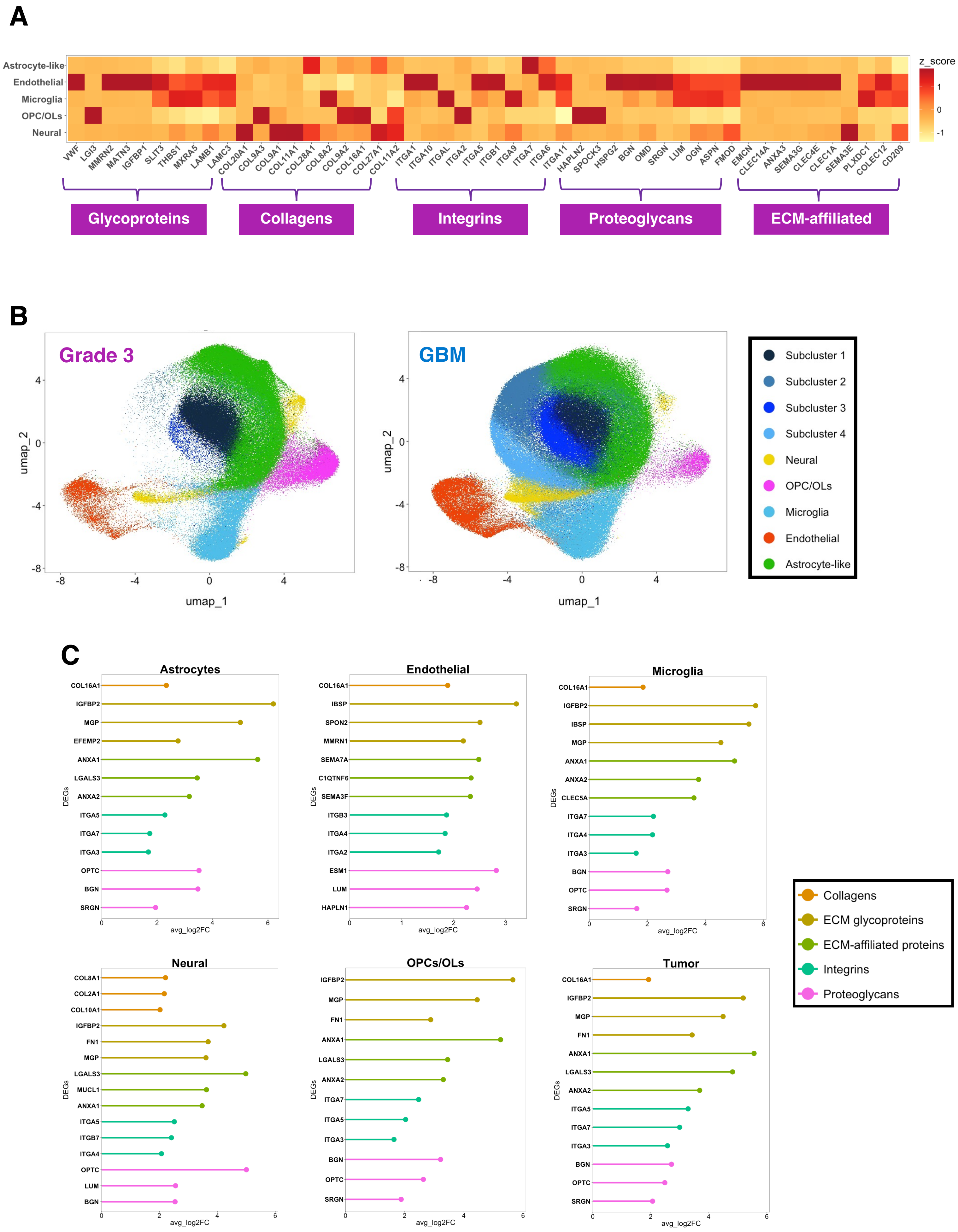

A
Astrocyte-like
Endothelial
Microglia
OPC/OLs
Neural
z_score
1
0
-1
VWF LGI3 MMRN2 MATN3 IGFBP1 SLIT3 THBS1 MXRA5 LAMB1 LAMC3 COL20A1 COL9A3 COL9A1 COL11A1 COL28A1 COL8A2 COL9A2 COL16A1 COL27A1 COL11A2 ITGA1 ITGA10 ITGAL ITGA2 ITGA5 ITGB1 ITGA9 ITGA7 ITGA6 ITGA11 HAPLN2 SPOCK3 HSPG2 BGN OMD SRGN LUM OGN ASPN FMOD EMCN CLEC14A ANXA3 SEMA3G CLEC4E CLEC1A SEMA3E PLXDC1 COLEC12 CD209
Glycoproteins
Collagens
Integrins
Proteoglycans
ECM-affiliated
B
Grade 3
GBM
umap_2
umap_1
Subcluster 1
Subcluster 2
Subcluster 3
Subcluster 4
Neural
OPC/OLs
Microglia
Endothelial
Astrocyte-like
C
Astrocytes
COL16A1 IGFBP2 MGP EFEMP2 ANXA1 LGALS3 ANXA2 ITGA5 ITGA7 ITGA3 OPTC BGN SRGN
Endothelial
COL16A1 IBSP SPON2 MMRN1 SEMA7A C1QTNF6 SEMA3F ITGB3 ITGA4 ITGA2 ESM1 LUM HAPLN1
Microglia
COL16A1 IGFBP2 IBSP MGP ANXA1 ANXA2 CLEC5A ITGA7 ITGA4 ITGA3 BGN OPTC SRGN
Neural
COL8A1 COL2A1 COL10A1 IGFBP2 FN1 MGP LGALS3 MUCL1 ANXA1 ITGA5 ITGB7 ITGA4 OPTC LUM BGN
OPCs/OLs
IGFBP2 MGP FN1 ANXA1 LGALS3 ANXA2 ITGA7 ITGA5 ITGA3 BGN OPTC SRGN
Tumor
COL16A1 IGFBP2 MGP FN1 ANXA1 LGALS3 ANXA2 ITGA5 ITGA7 ITGA3 BGN OPTC SRGN
DEGs
avg_log2FC
Collagens
ECM glycoproteins
ECM-affiliated proteins
Integrins
Proteoglycans

Supp. Fig. 4

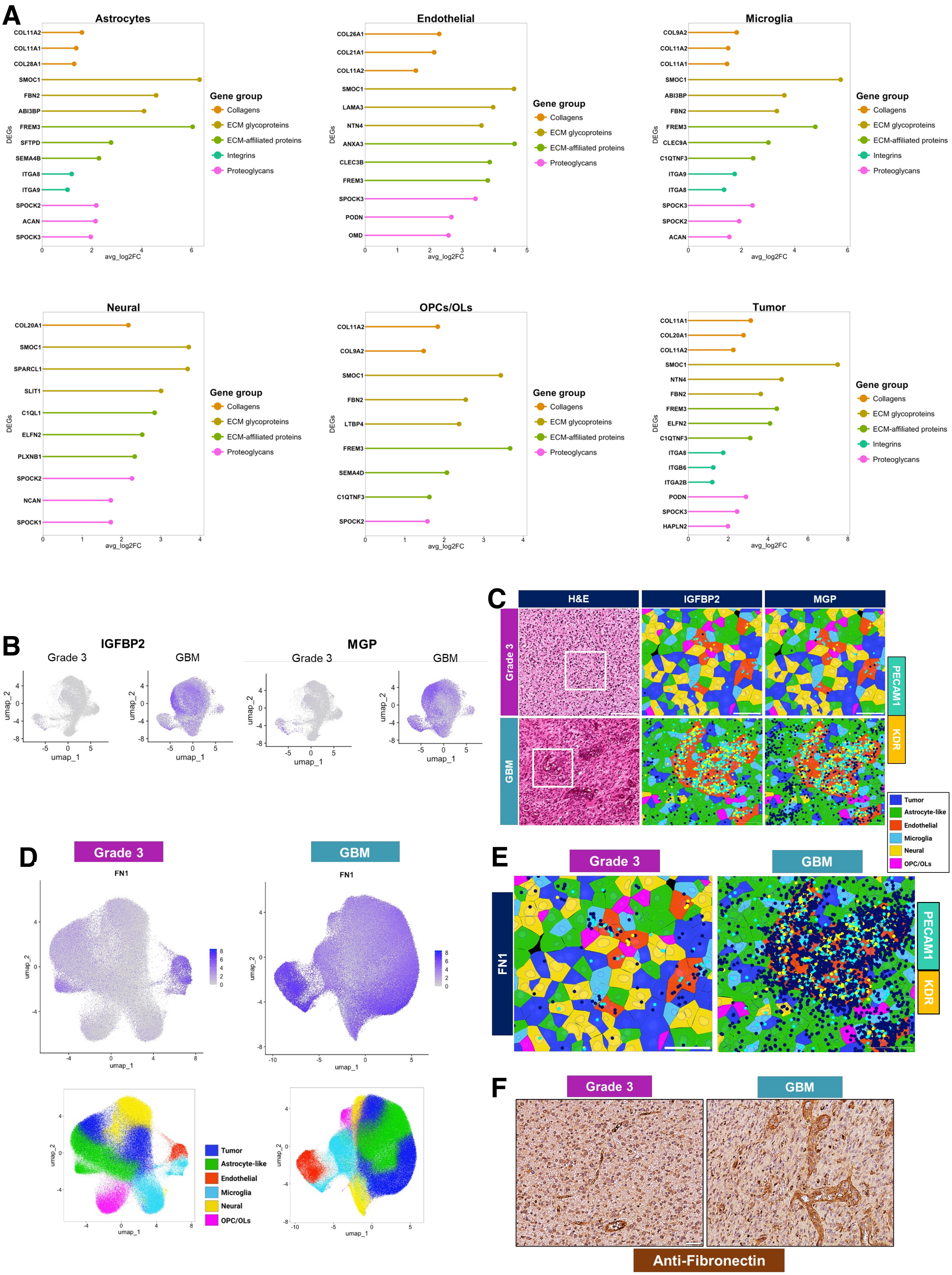
A
Astrocytes
Endothelial
Microglia
Neural
OPCs/OLs
Tumor
DEGs
avg_log2FC
Gene group
Collagens
ECM glycoproteins
ECM-affiliated proteins
Integrins
Proteoglycans
COL11A2
COL11A1
COL28A1
SMOC1
FBN2
ABI3BP
FREM3
SFTPD
SEMA4B
ITGA8
ITGA9
SPOCK2
ACAN
SPOCK3
COL26A1
COL21A1
LAMA3
NTN4
ANXA3
CLEC3B
PODN
OMD
COL9A2
CLEC9A
C1QTNF3
COL20A1
SPARCL1
SLIT1
C1QL1
ELFN2
PLXNB1
NCAN
SPOCK1
LTBP4
SEMA4D
ITGB6
ITGA2B
HAPLN2
B
IGFBP2
MGP
Grade 3
GBM
umap_1
umap_2
C
H&E
IGFBP2
MGP
PECAM1
KDR
Tumor
Astrocyte-like
Endothelial
Microglia
Neural
OPC/OLs
D
FN1
E
F
Anti-Fibronectin

Supp. Fig. 5

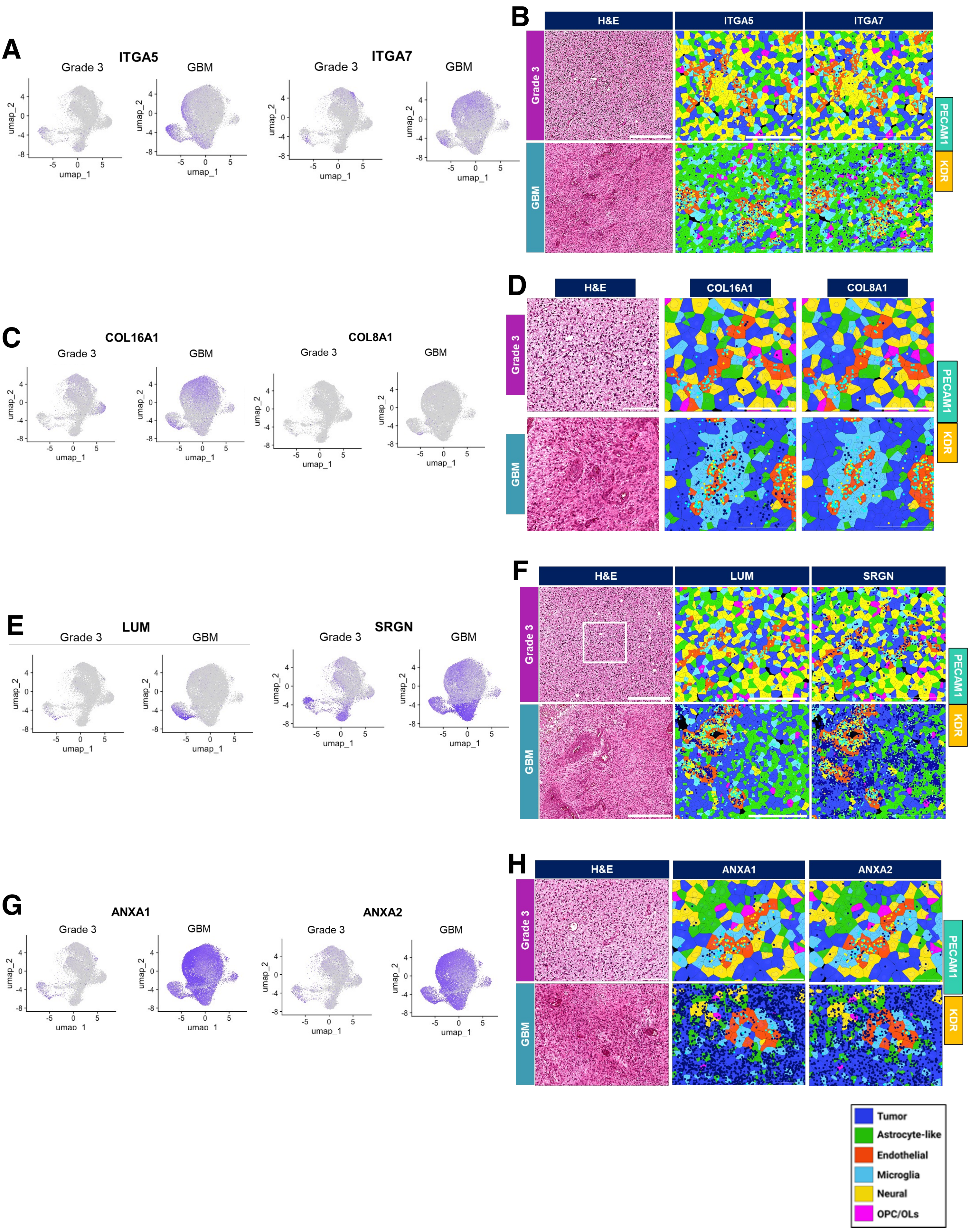

A
ITGA5
ITGA7
Grade 3
GBM
umap_1
umap_2
B
H&E
ITGA5
ITGA7
PECAM1
KDR
C
COL16A1
COL8A1
D
H&E
COL16A1
COL8A1
E
LUM
SRGN
F
H&E
LUM
SRGN
G
ANXA1
ANXA2
H
H&E
ANXA1
ANXA2
Tumor
Astrocyte-like
Endothelial
Microglia
Neural
OPC/OLs

**Supp. Fig. 6**

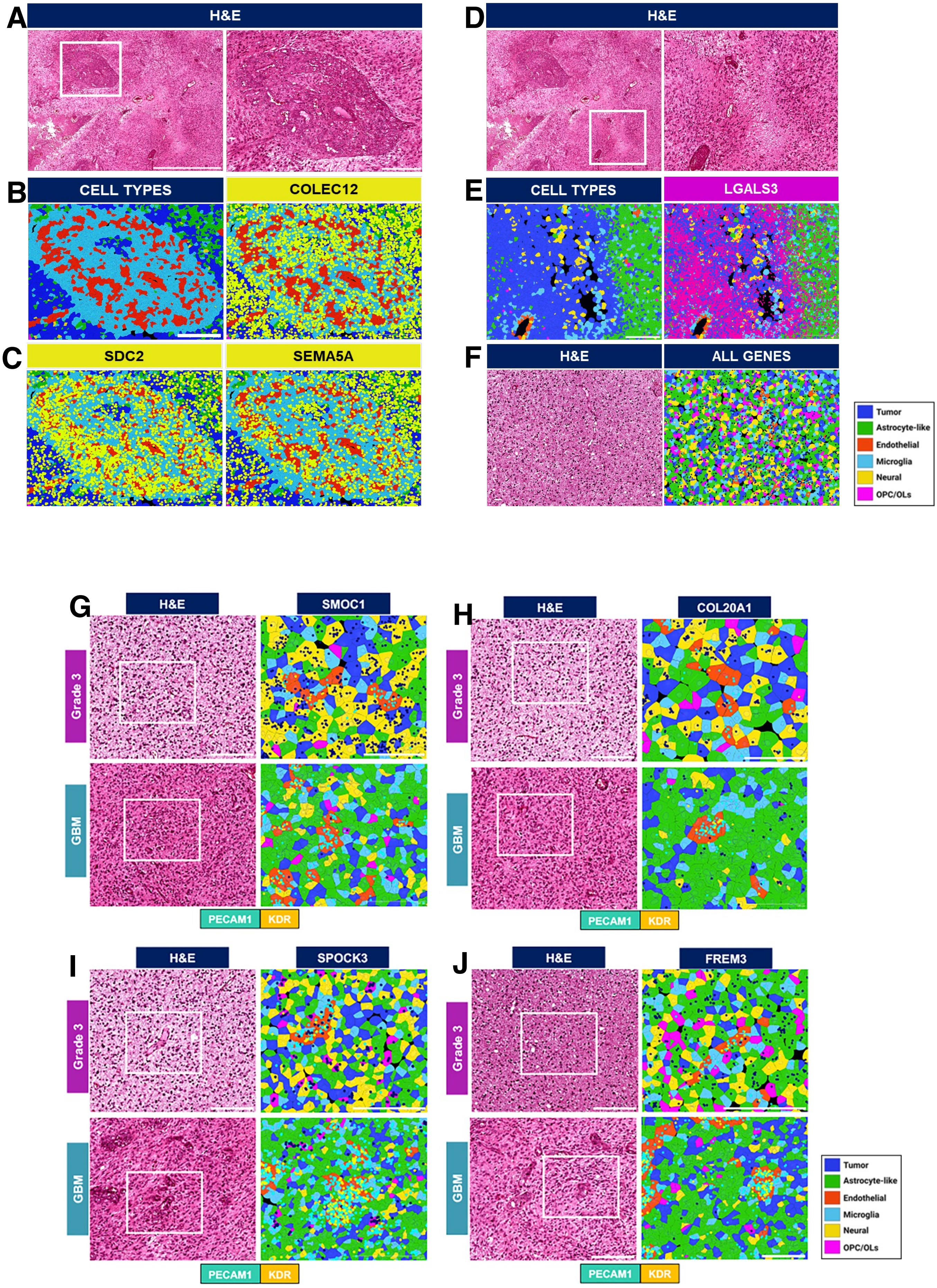
A
H&E
B
CELL TYPES
COLEC12
C
SDC2
SEMA5A
D
H&E
E
CELL TYPES
LGALS3
F
H&E
ALL GENES
Tumor
Astrocyte-like
Endothelial
Microglia
Neural
OPC/OLs
G
H&E
SMOC1
Grade 3
GBM
PECAM1
KDR
H
H&E
COL20A1
Grade 3
GBM
PECAM1
KDR
I
H&E
SPOCK3
Grade 3
GBM
PECAM1
KDR
J
H&E
FREM3
Grade 3
GBM
PECAM1
KDR
Tumor
Astrocyte-like
Endothelial
Microglia
Neural
OPC/OLs

Supp. Fig. 7

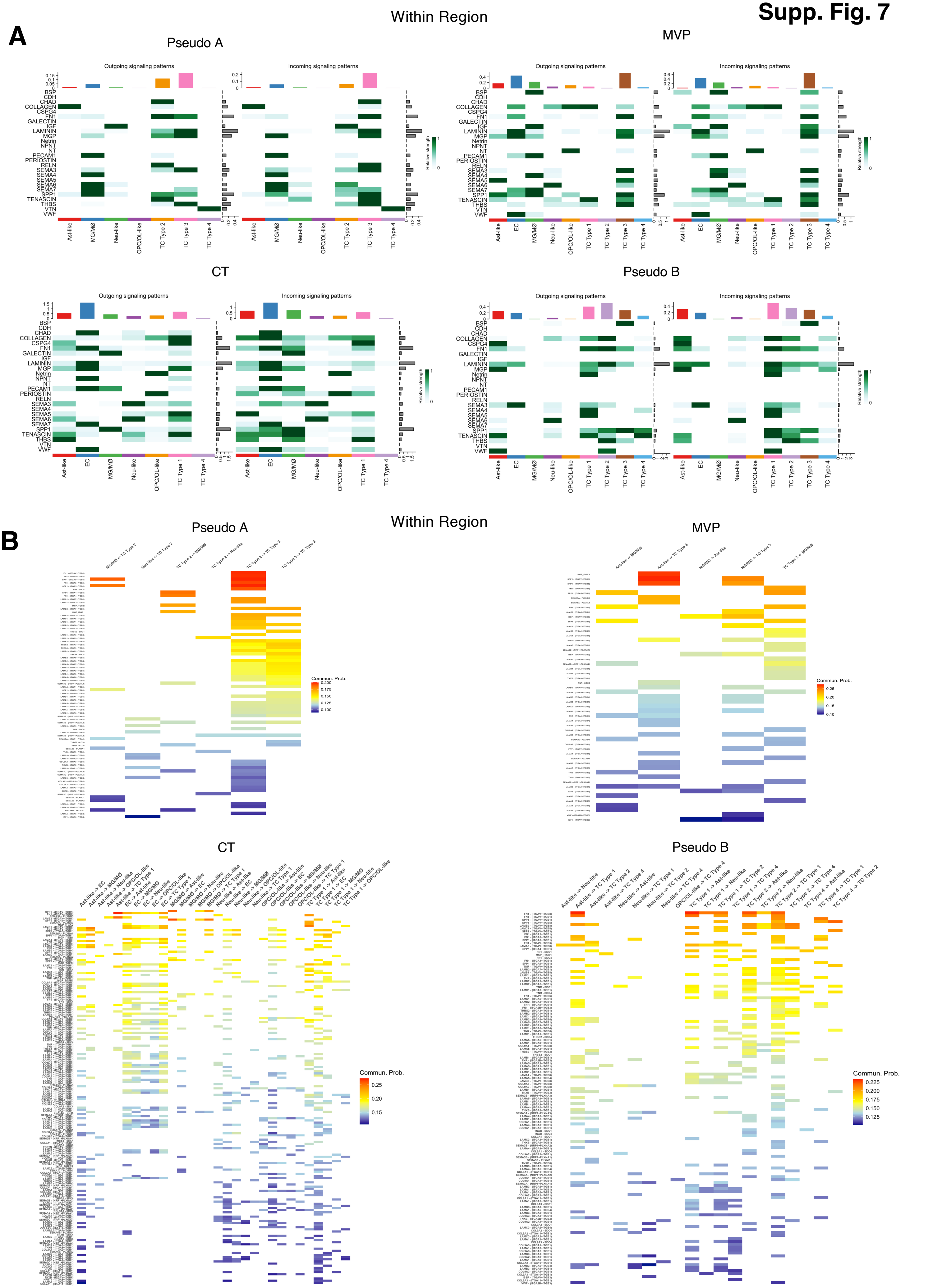
Within Region
A
Pseudo A
MVP
CT
Pseudo B
Outgoing signaling patterns
Incoming signaling patterns
BSP
CDH
CHAD
COLLAGEN
CSPG4
FN1
GALECTIN
IGF
LAMININ
MGP
Netrin
NPNT
NT
PECAM1
PERIOSTIN
RELN
SEMA3
SEMA4
SEMA5
SEMA6
SEMA7
SPP1
TENASCIN
THBS
VTN
VWF
Relative strength
Ast-like
EC
MG/MØ
Neu-like
OPC/OL-like
TC Type 1
TC Type 2
TC Type 3
TC Type 4
B
Commun. Prob.

Across Region

**Supp. Fig. 8**

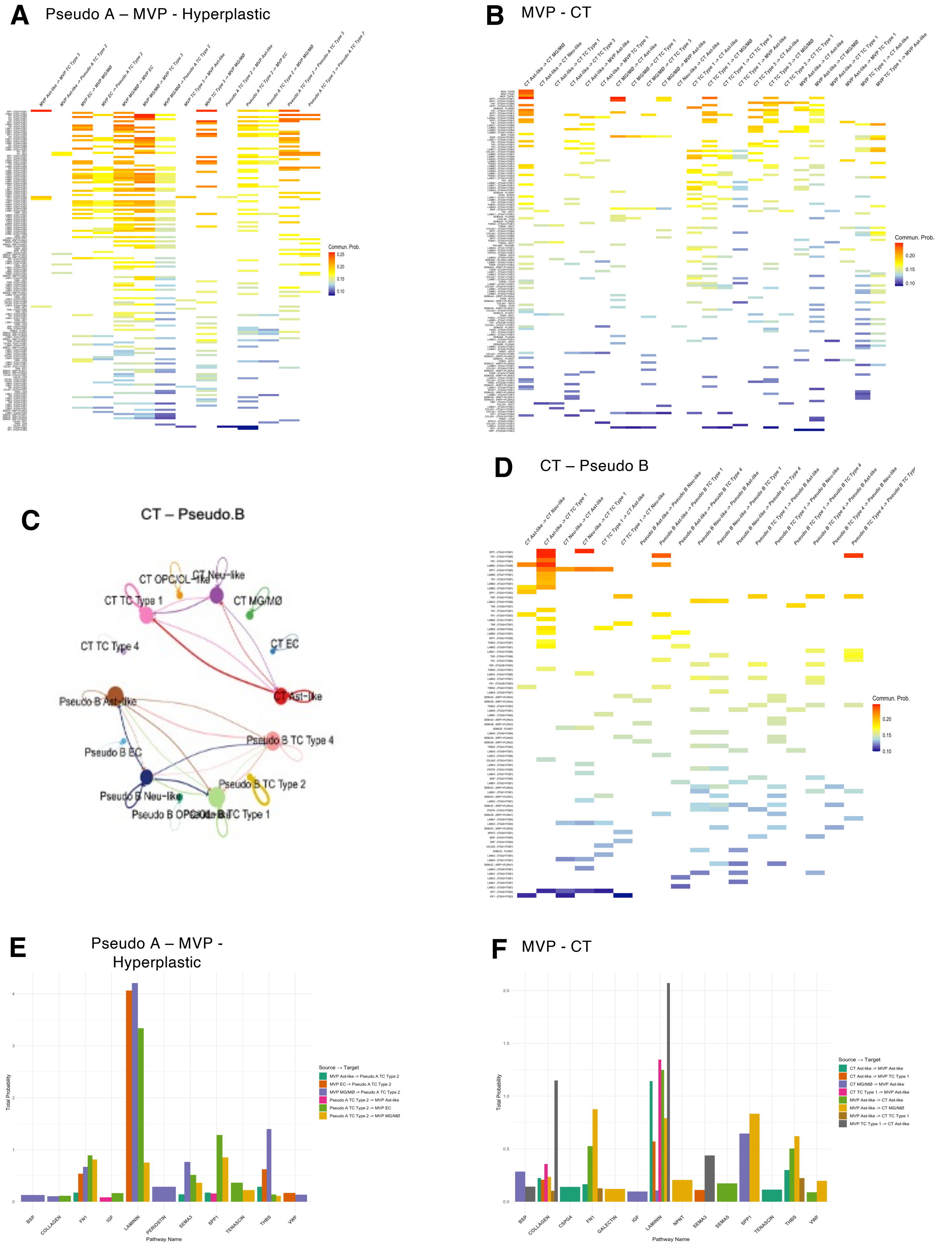

Supp. Fig. 9

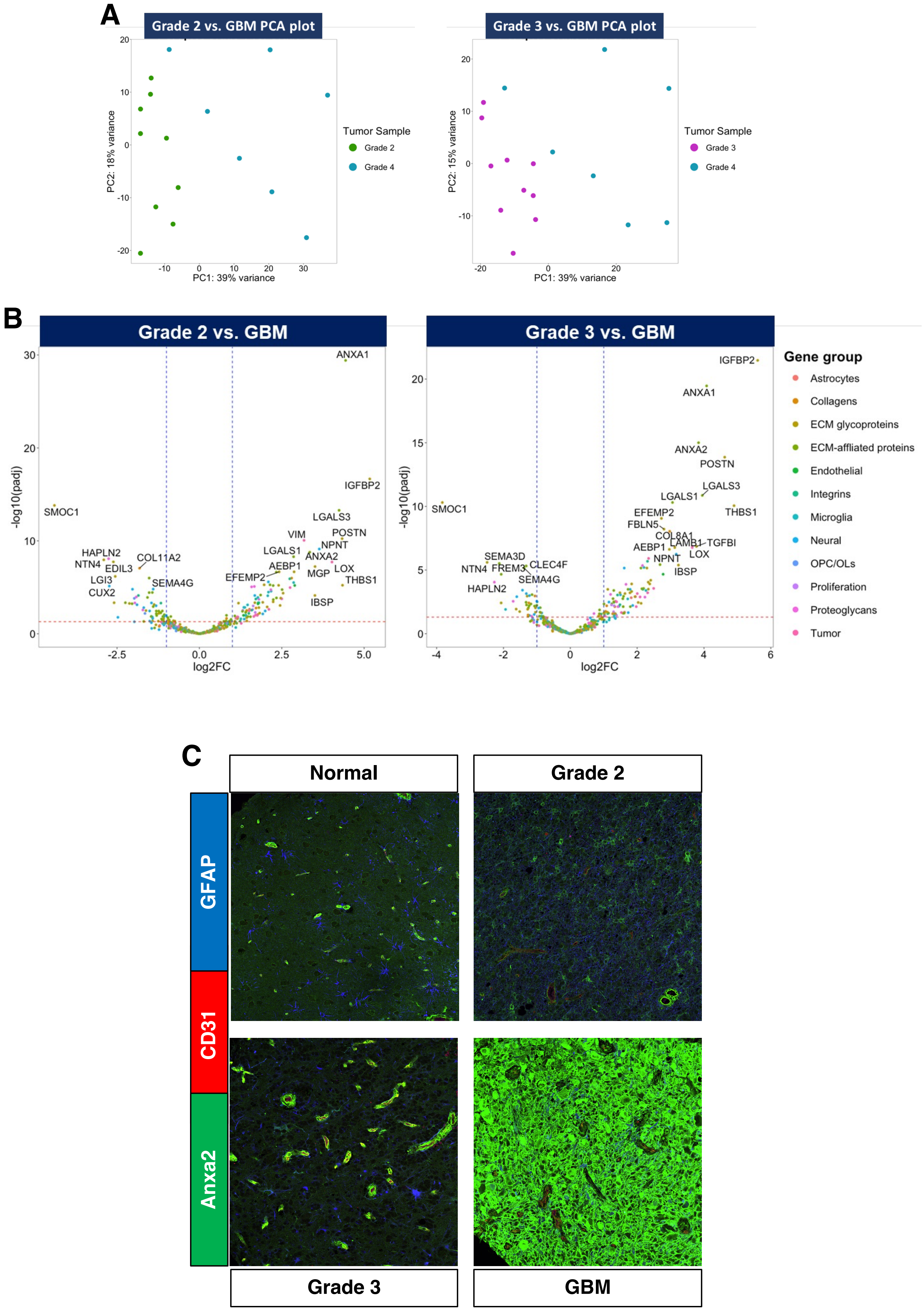
A
Grade 2 vs. GBM PCA plot
PC2: 18% variance
PC1: 39% variance
Tumor Sample
Grade 2
Grade 4
Grade 3 vs. GBM PCA plot
PC2: 15% variance
PC1: 39% variance
Tumor Sample
Grade 3
Grade 4
B
Grade 2 vs. GBM
-log10(padj)
log2FC
ANXA1
IGFBP2
SMOC1
LGALS3
VIM
POSTN
NPNT
LGALS1
ANXA2
AEBP1
MGP
LOX
THBS1
EFEMP2
IBSP
HAPLN2
NTN4
EDIL3
COL11A2
LGI3
CUX2
SEMA4G
Grade 3 vs. GBM
IGFBP2
ANXA1
ANXA2
POSTN
LGALS3
LGALS1
THBS1
EFEMP2
FBLN5
COL8A1
LAMB1
TGFBI
AEBP1
NPNT
LOX
IBSP
SEMA3D
NTN4
FREM3
CLEC4F
SEMA4G
HAPLN2
Gene group
Astrocytes
Collagens
ECM glycoproteins
ECM-affliated proteins
Endothelial
Integrins
Microglia
Neural
OPC/OLs
Proliferation
Proteoglycans
Tumor
C
Normal
Grade 2
GFAP
CD31
Anxa2
Grade 3
GBM

Supp. Fig. 10

A

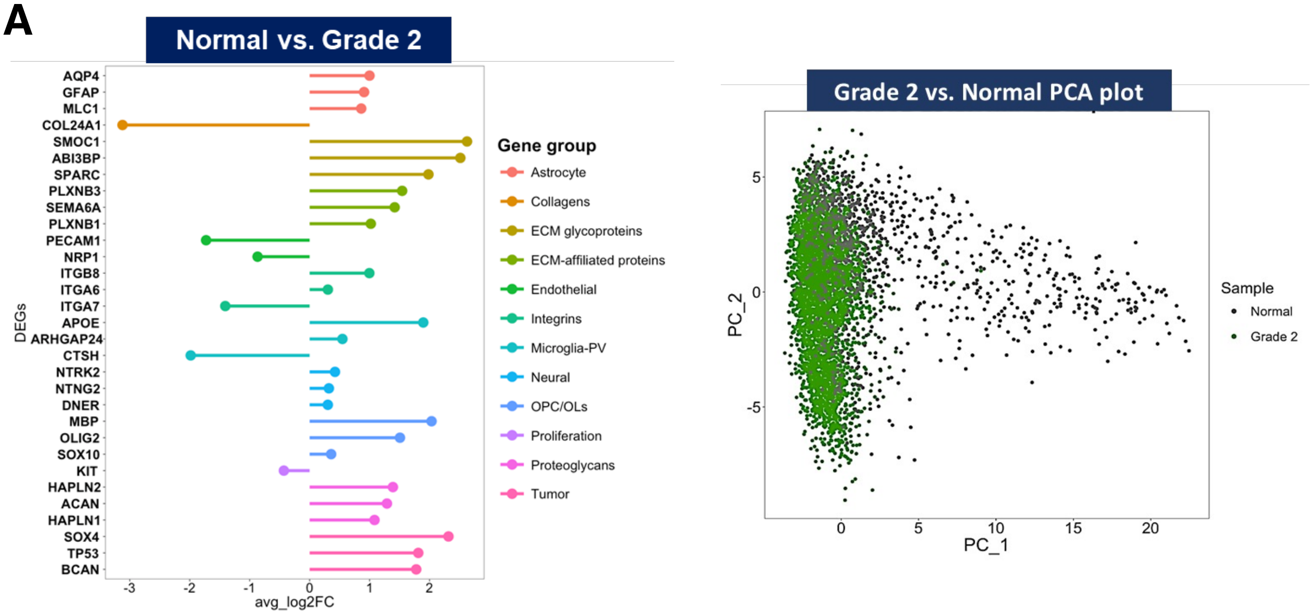


B

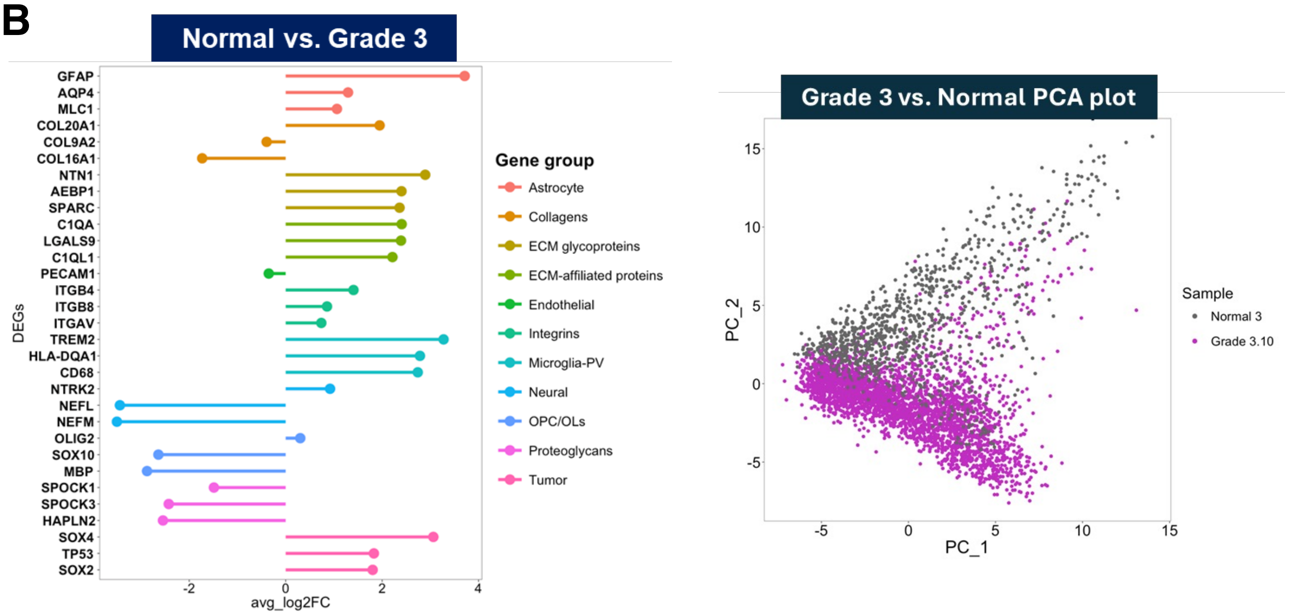


C

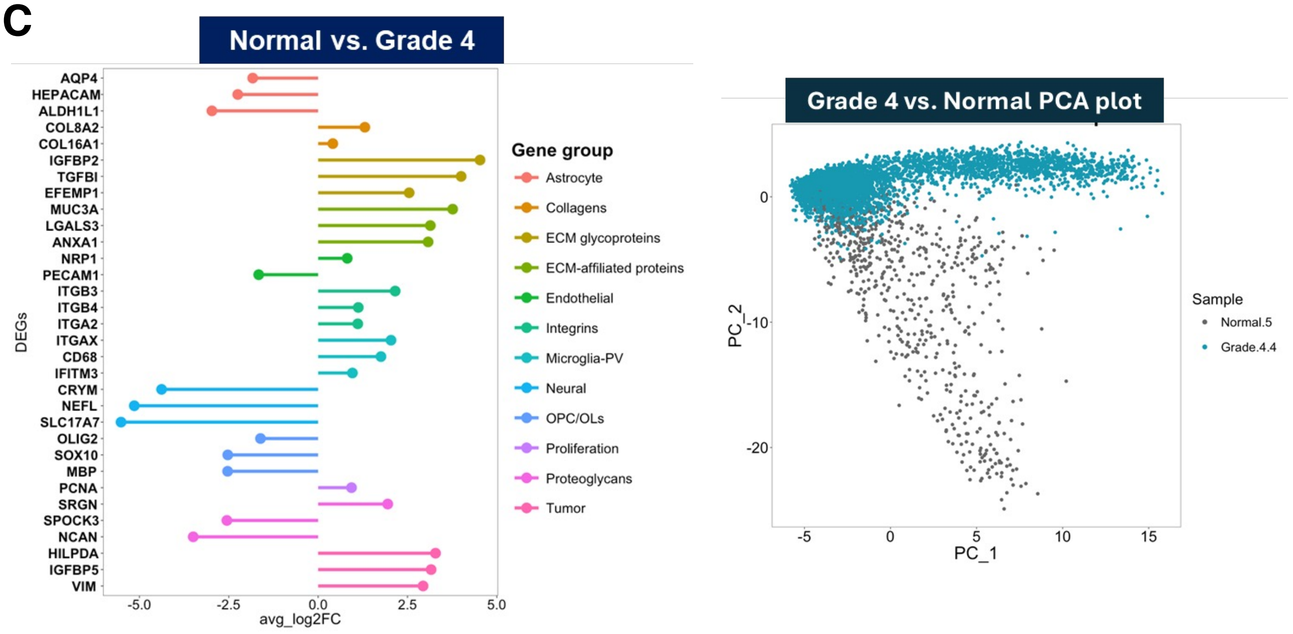

**Supp. Fig. 11**

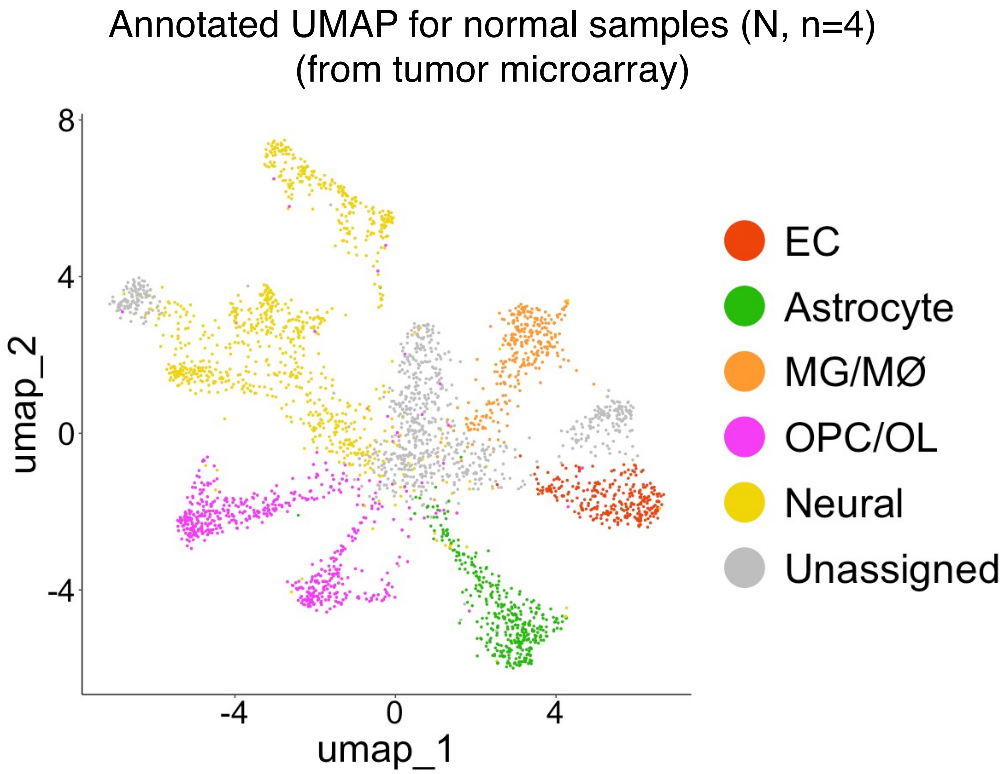
Annotated UMAP for normal samples (N, n=4)
(from tumor microarray)
umap_2
umap_1
8
4
0
-4
-4
0
4
EC
Astrocyte
MG/MØ
OPC/OL
Neural
Unassigned

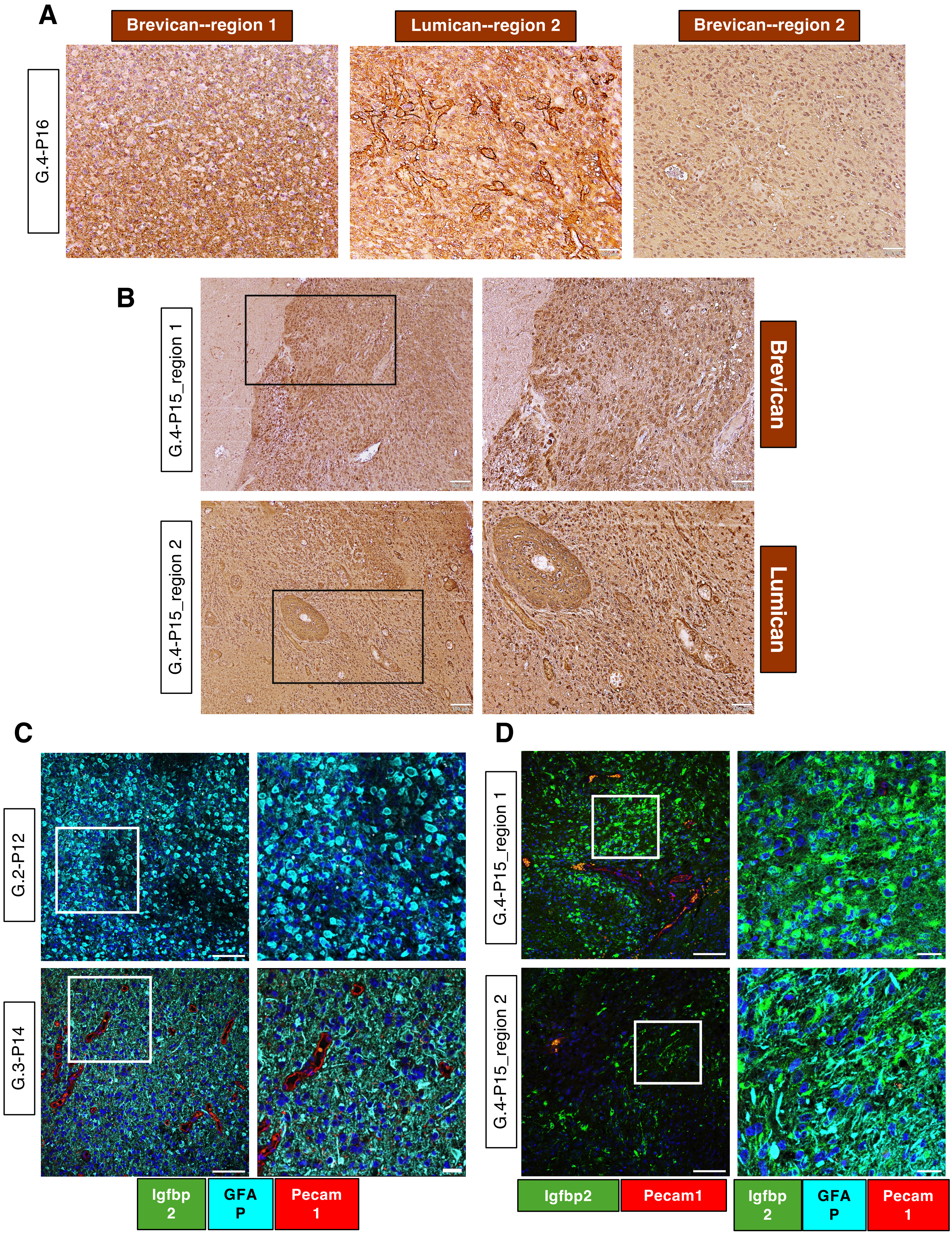
A
Brevican--region 1
Lumican--region 2
Brevican--region 2
G.4-P16
B
G.4-P15_region 1
Brevican
G.4-P15_region 2
Lumican
C
G.2-P12
G.3-P14
Igfbp 2
GFA P
Pecam 1
D
G.4-P15_region 1
G.4-P15_region 2
Igfbp2
Pecam1
Igfbp 2
GFA P
Pecam 1

# Supp. Fig. 13

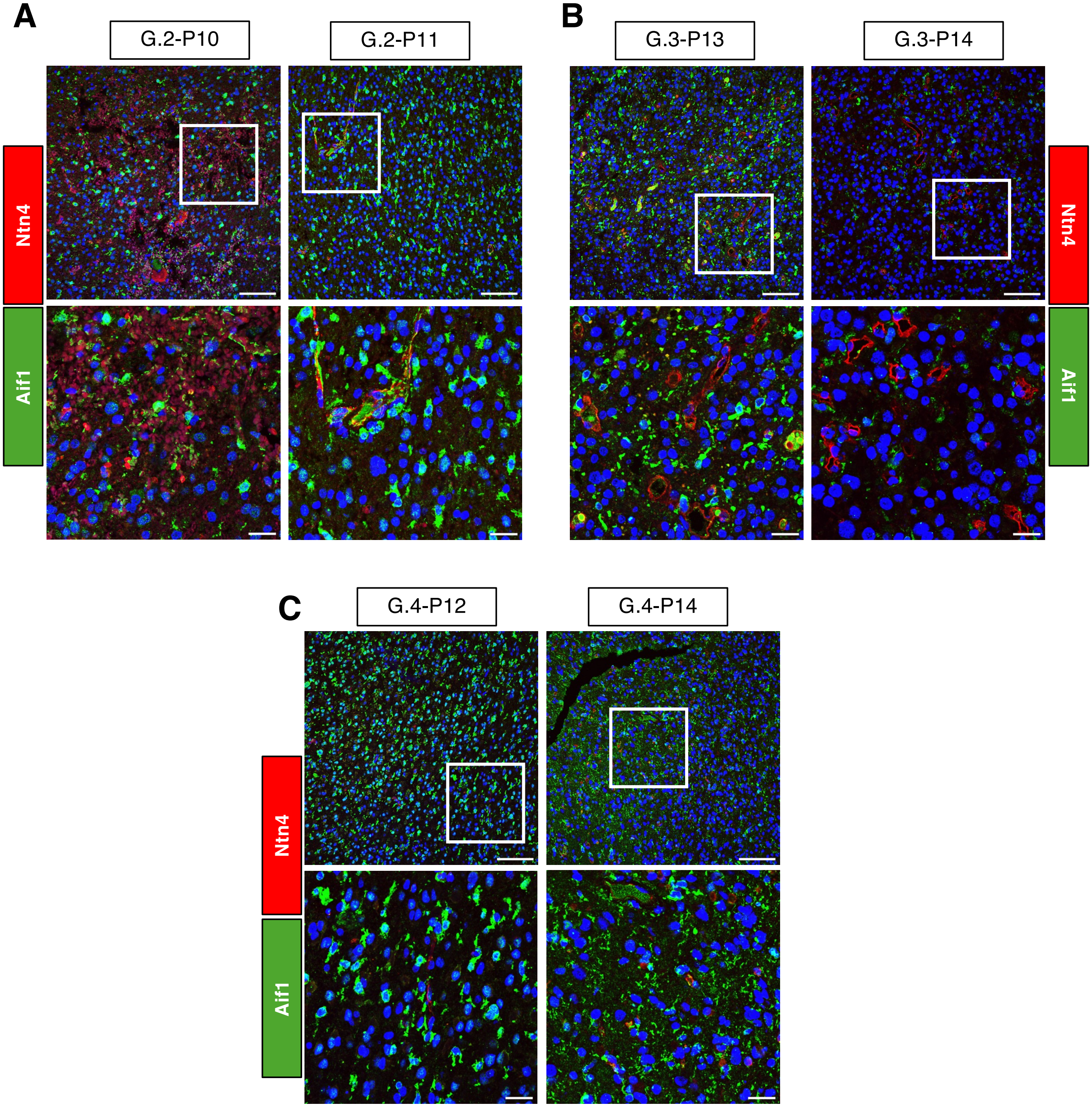
A
G.2-P10
G.2-P11
Ntn4
Aif1
B
G.3-P13
G.3-P14
Ntn4
Aif1
C
G.4-P12
G.4-P14
Ntn4
Aif1

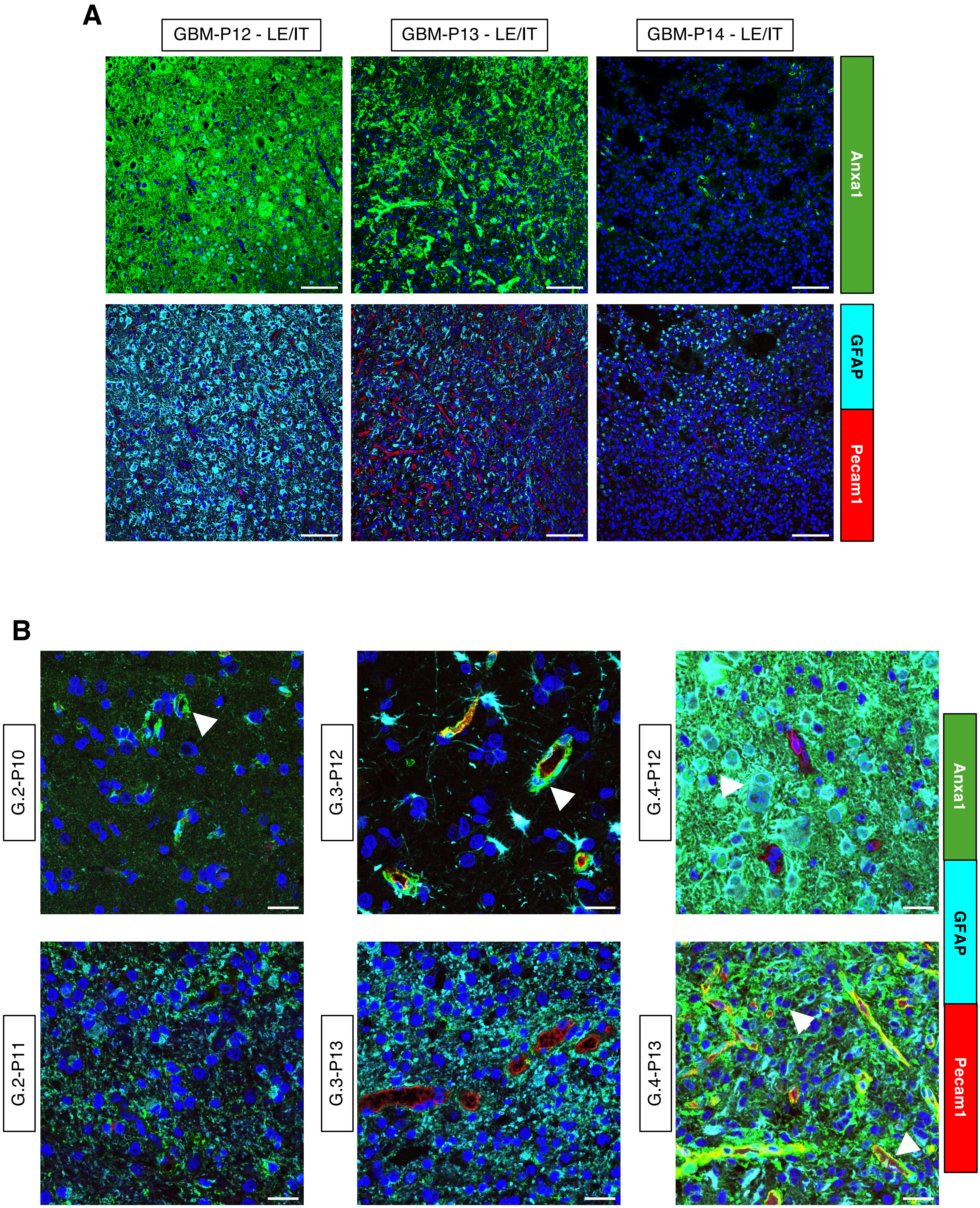

A
GBM-P12 - LE/IT
GBM-P13 - LE/IT
GBM-P14 - LE/IT
Anxa1
GFAP
Pecam1
B
G.2-P10
G.3-P12
G.4-P12
G.2-P11
G.3-P13
G.4-P13
Anxa1
GFAP
Pecam1

## Supplemental Figure Legends

**Supplemental Figure 1. High resolution *in situ* spatial mapping of tumor and matrisome genes in grade III astrocytoma. (A);** UMAP plot of a human grade III astrocytoma sample (n=1; IDH1 R132H) showing pseudo-colored clusters based on matrisome and cell-type specific marker gene expression profiles. **(B);** Graph showing the percentages for the six different stromal and cancer cell types within the grade III sample identified from the UMAP plot (left). Representative image of the grade III astrocytoma sample (n=1) showing different cell types assigned pseudo-colors (right) based on UMAP clustering (A). Bottom panel shows magnified image of the boxed area with high-resolution cell segmentation and spatial annotation. Scale bars: 200 µm (upper) and 100 µm (lower)**. (C);** H&E-stained image of a grade III astrocytoma tissue section (top, left) showing viable tumor. Heatmaps from spatial single cell transcript analysis showing EGFR (top, right), and VIM (bottom, left). Spatial segmentation from UMAP analysis showing annotation of different cell types based on gene enrichment in the viable tumor tissue (bottom, right). Scale bar: 5000 µm. **(D);** UMAP plot (left) showing four tumor sub-clusters identified by in-depth single cell transcript expression profiling shown in A. Heatmap (right) showing expression profiles of TC and proliferation markers in the four TC sub-clusters. **(E);** Heatmap showing transcript enrichment of select matrisome genes in different TC subtypes. Shown on the horizontal axis are top DEG from each matrisome category. **(F);** Representative H&E-stained section (left panel) and pseudocolored cell images (right panel) for the grade III astrocytoma sample reveals uniform spatial patterning of TC subclusters. Scale bars: 500 µm. **(G);** Representative H&E (top) and pseudocolored cells (bottom) for four different grade III astrocytoma areas reveals non-distinct and largely overlapping spatial positioning of TCs belonging to the four different subclusters. Scale bars: 500 µm.

**Supplemental Figure 2. Cell-type specific matrisome gene expression validation. (A);** Sub-annotated UMAP (left) highlighting stromal cells and CT malignant cells from the Spitzer et

al. scRNA-seq dataset (n=59 samples). FeaturePlots showing GPC2 (middle) and NCAN (right) transcript expression levels. **(B);** Sub-annotated UMAP (left) highlighting stromal cells and Pseudo malignant cells from Spitzer et al. scRNA-seq dataset. FeaturePlots showing LGALS3 (middle) and IGFBP2 (right) transcript expression levels. **(C);** Sub-annotated UMAP (left) highlighting stromal cells and MVP malignant cells from Spitzer et al. scRNA-seq dataset. FeaturePlots showing ESM1 (middle) and THBS1 (right) transcript expression levels. **(D);** FeaturePlots of malignant cells, endothelial cells (red square), astrocytes (green square), and tumor associated macrophages (TAM; blue square) showing combined expression of genes in each row (left column). FeaturePlots of filtered Endothelial cells, Astrocytes, and TAMs are shown in consecutive panels in respective order. Top row shows expression levels of CT region-specific DEGs enriched for each cell type (Endothelial: TNXB, Astrocytes: SPON1, TAM: TNR). Middle row shows expression levels of Pseudo region-specific DEGs enriched for each cell-type (Endothelial: SPP1, Astrocytes: FREM2, TAM: ANXA1). Bottom row shows expression levels of MVP region-specific DEGs enriched for each cell type (Endothelial: ESM1, Astrocytes: COLEC12, TAM: THBS1).

**Supplemental Figure 3. Spatial gene expression analysis of stromal cell types in grade III astrocytoma and GBM. (A);** Heatmap showing enrichment of selected matrisome transcripts in different single cell types in the grade III astrocytoma sample (n=1). Shown on the horizontal axis are the top DEGs from each group: ECM glycoproteins, collagens, integrins, proteoglycans, and ECM-affiliated factors. **(B);** Integrated UMAP plots from the human grade III astrocytoma sample (left; n=1) and GBM sample (right, n=1) showing pseudo-colored cell clusters based on matrisome gene expression profiles. **(C);** Lollipop plots from integrated UMAP subclusters (B) showing transcript expression profiles for different stromal cells and cancer cell clusters in the GBM sample. All DEGs in the lollipop plots are statistically significant ($p < 0.0001$). All transcripts show higher expression in single cells from the GBM versus the grade III astrocytoma.

**Supplemental Figure 4. DEGs in grade III astrocytoma sample and spatial mapping of upregulated matrisome genes in GBM. (A);** Single-cell transcript analysis of integrated UMAP cell subclusters, displayed as lollipop plots, showing statistically significant differential gene expression profiles for stromal cells and TC clusters in grade III astrocytoma ($p < 0.0001$). All transcripts display higher expression in cells from the grade III astrocytoma sample (n=1) versus the GBM sample (n=1). **(B);** Integrated UMAP plots showing elevated IGFBP2 (left) and MGP (right) gene expression in multiple cell types in the GBM sample versus the grade III astrocytoma sample. **(C);** Representative images showing *in situ* spatial distribution of the ECM glycoprotein transcripts IGFBP2 and MGP at single-cell resolution. Cells are pseudo-colored based on UMAP analyses of grade III and GBM samples. The right panels are magnified regions from the boxed region of interest (H&E) of the grade III (top) and GBM (bottom) samples. In comparison to grade III astrocytoma, both transcripts show significantly increased expression in different cell types proximal to ECs in the GBM sample. PECAM1; cyan, KDR; yellow and ECM glycoprotein gene transcripts; dark blue. Scale bars: 200 $\mu$m for H&E images and 50 $\mu$m for pseudocolored images. **(D);** UMAP plot for the grade III astrocytoma sample (left) showing FN1 expression primarily in the vascular EC cluster and in some astrocyte-like cells and microglia. This is different than the UMAP plot (right) showing robust FN1 enrichment in multiple cell types in addition to vascular ECs in a GBM sample. The UMAP plots along the bottom show the pseudo-colored cell cluster identities. **(E);** In comparison to grade III astrocytoma (left), there is elevated FN1 mRNA (dark blue transcripts) in GBM (right). **(F);** Representative images showing elevated fibronectin protein expression in GBM by immunohistochemistry. These data validate the spatial transcriptome analysis. Scale bar: 50 $\mu$m.

**Supplemental Figure 5. High resolution *in situ* single cell spatial mapping for genes encoding integrins, collagens, proteoglycans and ECM-affiliated factors. (A);** Integrated

UMAP plots revealing elevated ITGA5 (left) and ITGA7 (right) transcripts in multiple cell types in the GBM sample (n=1) versus the grade III astrocytoma sample (n=1). **(B);** Representative images showing spatial localization of select integrin transcripts (ITGA5 and ITGA7) at single-cell resolution. Shown are magnified regions from boxed ROIs (H&E) of the grade III astrocytoma (top) and GBM (bottom). In comparison to grade III sample, ITGA5 transcripts (dark blue) show higher expression in ECs whereas ITGA7 transcripts (dark blue) show elevated expression in perivascular astrocyte-like cells and cancer cells within the GBM sample. PECAM1; cyan, KDR; yellow. Scale bars: 500 $\mu$m for H&E images and 200 $\mu$m for high-resolution single-cell images. **(C);** Integrated UMAP plots revealing elevated COL16A1 (left) and COL8A1 (right) gene expression in multiple cell types in the GBM sample versus the grade III astrocytoma sample. **(D);** Representative images showing spatial distribution of selected collagen transcripts (COL16A1 and COL8A1) at single cell resolution (cells pseudo-colored) in the magnified region from boxed region of interest (H&E) of the grade III astrocytoma (top) and GBM (bottom) tissue sections. In comparison to the grade III tumor, both selected collagen genes show enhanced expression in ECs (red), microglia (light blue), astrocyte-like cells (green), and TCs (dark blue) in the GBM sample. PECAM1; cyan, KDR; yellow and collagen gene transcripts; dark blue. Scale bars: 200 $\mu$m for H&E images and 100 $\mu$m for single cell images. **(E);** Integrated UMAP plots showing elevated LUM (left) and SRGN (right) transcripts in different cell types in the GBM sample versus the grade III astrocytoma sample. Note that LUM expression increases primarily in ECs whereas increased SRGN expression is detected in multiple cell types. **(F);** Representative images showing spatial distribution of proteoglycan transcripts LUM and SRGN at single-cell resolution in the magnified region from boxed ROI (H&E) of the grade III (top) and GBM sample (bottom). In comparison to grade III astrocytoma sample, LUM shows enhanced transcript levels primarily in ECs whereas SRGN shows transcript enrichment in tumor and different stromal cell types in the GBM sample. PECAM1; cyan, KDR; yellow and proteoglycan gene transcripts; dark blue. Scale bars: 500 $\mu$m for H&E images and 200 $\mu$m for pseudocolored images. **(G);** Integrated UMAPs showing elevated

ANXA1 (left) and ANXA2 (right) gene expression in different cell types in the GBM sample versus the grade III astrocytoma sample. **(H);** Representative images showing *in situ* spatial distribution of ECM-affiliated transcripts ANXA1 and ANXA2 at single cell resolution (cells pseudo-colored) in the magnified region from boxed ROI (H&E) of the grade III tissue (top) and GBM (bottom). Both ANXA1 and ANXA2 transcripts show significantly increased expression predominantly in TCs (dark blue) and other cell types: astrocyte-like (green) and microglia (light blue), as well as in ECs (red) in the GBM sample. PECAM1; cyan, KDR; yellow and ECM-affiliated gene transcripts; dark blue. Scale bars: 200 $\mu$m for H&E images and 50 $\mu$m for pseudo-colored images.

**Supplemental Figure 6. Analysis of matrisome gene expression in regions of neuroinflammation in GBM. (A);** Representative ROI in H&E-stained GBM sample (n=1) showing perivascular microgliosis. Boxed area on the left is shown as a magnified image on the right. Scale bars: 100 $\mu$m (left) and 200 $\mu$m (right). **(B);** Single cell spatial analysis of boxed region shown in (A) identifies clusters of microglial cells (light blue) mixed with ECs (red), (left, B). Note the presence of GBM cells (dark blue) and astrocyte-like cells (green) in the surrounding region. Scale bars: 200 $\mu$m. ECM-affiliated transcript COLEC12 (right, B) shows enhanced expression in diverse cell types identified. COLEC12 transcripts are shown in yellow. Scale bars: 200 $\mu$m**. (C);** SDC2 (left) and SEMA5A (right) show elevated expression in different cell types localized around necrosis. All gene transcripts are shown in yellow. Scale bars: 200 $\mu$m**. (D);** Representative region of interest in H&E-stained GBM sample showing a region with TC pseudopalisading. Boxed area on the left is shown as a magnified image on the right. Scale bars: 100 $\mu$m (left) and 200 $\mu$m (right). **(E);** Single cell transcript analysis of the region shown in (D), highlighting abundant tumor (dark blue) and astrocyte-like (green) cell populations along with less abundant microglia (light blue), neural (yellow) and ECs (red). Increased LGALS3 transcripts (dark pink) in TCs (dark blue) compared to astrocyte-like cells (green) in the pseudopalisading region (right). Scale bar: 200 $\mu$m**. (F);** Representative images showing H&E-

stained grade III sample (left) and corresponding pseudo-colored cell types (right) display lower levels of transcripts for COLEC12, SDC2, SEMA5A and LGALS3 (all shown in dark blue) in different cell types (right). Scale bars: 200 $\mu$m. **(G-J);** Matrisome transcripts with reduced levels in GBM versus grade III astrocytoma. Representative images showing *in situ* single cell spatial transcript localization for grade III (top) and GBM (bottom) from the boxed ROIs shown in corresponding H&E images reveal comparatively elevated cellular transcript enrichment of ECM glycoprotein SMOC1 (G), collagen COL20A1 (H), proteoglycan SPOCK3 (I) and ECM-affiliated gene FREM3 (J) in grade III astrocytoma. *In situ* spatial localization of matrisome transcripts (dark blue) in pseudo-colored cells with the EC markers PECAM1 (cyan) and KDR (yellow). Scale bars: 200 $\mu$m for H&E images, and 100 $\mu$m for high-resolution single-cell spatial transcriptomic images.

**Supplemental Figure 7. Cell-cell communication networks within GBM anatomic regions.**

**(A);** Heatmap representation of signaling networks showing outgoing and incoming signaling patterns within the Pseudo.A, MVP, CT and Pseudo.B regions from the GBM sample (n=1). The intensity of each color indicates the strength of ligand-receptor interactions within each pathway, with stronger signals shown in darker green shades. **(B);** Communication probability of all the Ligand-Receptor pairs within regions showing only above-mean communication probability and sorted by their probabilities.

**Supplemental Figure 8. Communication probabilities for signaling pathways inferred by CellChat. (A);** Communication probability of all ligand-receptor pairs across the regions from the GBM sample (n=1), showing only above-mean communication probability. **(B);** Communication probabilities of all the signaling pathways for Pseudo.A-MVP-Hyperplastic and MVP-CT regions from the GBM sample (n=1). **(C);** Chord plot summarizing interactions between region-specific cell types across the CT-Pseudo.B boundaries. There was no significant cellular crosstalk detected across the Pseudo.B-CT boundaries. **(D);** Heatmap

representation of networks showing outgoing and incoming signaling strength across CT-Pseudo.B boundary. Left panel shows outgoing signals from CT cell types (light green) to recipient Pseudo.B region cell types (dark green). Right panel shows outgoing signals from Pseudo.B region cell types (dark green) to recipient Pseudo.B cell types (light green). **(E);** Bar plot showing distribution of communication probabilities (taken as a sum of all L-R pairs probability) for all the signaling pathways across the region (Pseudo-MVP-Hyperplastic). The bars are colored by cell types showing the directionality of crosstalk across the boundaries. **(F);** Bar plot showing distribution of communication probabilities (taken as a sum of all LR pairs probability) for all the signaling pathways across the region MVP-CT. The bars are colored by cell types showing the directionality of crosstalk across the boundaries.

**Supplemental Figure 9. Single cell spatial analysis of matrisome expression in lower grade gliomas and GBM samples (A);** PCA plots comparing variance-based distribution of grade II diffuse glioma (n=9) and grade III astrocytoma (n=10) versus GBM (n=7) pseudo-bulk patient samples. **(B);** Volcano plot showing DEGs representing different cell markers and matrisome groups compared across grade II glioma (n=9) versus GBM (n=7) pseudo-bulk samples (left), and grade III astrocytoma (n=10) versus GBM (n=7) pseudo-bulk samples (right) resected from different patients. Selected genes are highlighted that show statistically significant (above horizontal red dotted line) $\log_2$ fold change ≥ +1.0 or ≤ -1.0 (right or left of vertical blue dotted lines respectively) in cellular transcript counts for GBM in comparison to grade II and grade III samples. All analyses were based on pseudo-bulk transcript counts from single cells. **(C);** Expression of annexin-a2 protein was analyzed in fixed sections from non-cancerous brain, grade II diffuse glioma, grade III astrocytoma, and GBM by immunofluorescence. Note that in support of the spatial transcriptomic data for ANXA2 expression (A-B), there is an increase in annexin-a2 protein levels in GBM versus lower grade brain tumor samples.

**Supplemental Figure 10. Single cell analysis of matrisome transcript expression in differentially graded astrocytoma samples versus matching non-cancerous brain tissues. (A);** Lollipop plot showing top DEGs across grade II (n=1) and matched normal sample (n=1). Differential gene expressions were determined by analyzing transcript counts from single cells identified by the xenium platform. Selected DEGs are from gene groups of the brain matrisome and cell-type specific markers (left). Also shown is the PCA plot representing variance-based distribution of single cells (pseudo-bulk analysis) from these grade II diffuse glioma and matched normal patient samples (right). **(B);** Lollipop plot showing top DEGs across grade III (n=1) and matched normal sample (n=1). Differential gene expressions were determined by analyzing transcript counts from single cells identified by the xenium platform. Selected DEGs are from gene groups of the brain matrisome and cell-type specific markers (left). Also shown is the PCA plot representing variance-based distribution of single cells (pseudo-bulk analysis) from these grade III astrocytoma and matched normal patient samples (right). **(C);** Lollipop plot showing top DEGs across GBM (n=1) and matched normal sample (n=1). Differential gene expressions were determined by analyzing transcript counts from single cells identified by the xenium platform. Selected DEGs are from gene groups of the brain matrisome and cell-type specific markers (left). Also shown is the PCA plot representing variance-based distribution of single cells (pseudo-bulk analysis) from these grade IV/GBM and matched normal patient samples (right).

**Supplemental Figure 11. Annotation of cell-types in non-cancerous brain samples.** UMAP showing unsupervised single-cell fine clustering of cell types identified (pseudo-bulk analysis) in patient-derived punch biopsy samples (n=4) from non-cancerous brain samples (normal; N) in the tissue microarray. Pseudo-colored cell clusters are defined by differential expression profiles for cell-type specific and related matrisome markers.

**Supplemental Figure 12. Analysis of region-specific ECM protein expression in GBM. (A);** Representative immunohistochemical images revealing elevated expression of Brevican protein in region 1, which is likely CT (left) and Lumican protein in region 2, which contains blood vessels indicative of MVP (middle) in a GBM sample (n=1). Note the significantly reduced expression of Brevican in the same MVP region (right). **(B);** Representative images showing enriched expression of Brevican (region 1, top panel) and Lumican (region 2, bottom panel) in different GBM sample (n=1) by immunohistochemistry. Magnified images of boxed areas for each region are shown on the right. Note the distinguishable CT and MVP regions identified by Brevican and Lumican expression, respectively. Scale bars: 100 $\mu$m. **(C, D);** Spatial enrichment of IGFBP2 protein in TCs within pseudopalisading regions of a GBM sample (n=1). Immunofluorescence labeling shows overall low expression of IGFBP2 protein (green) in both G.2 (n=1) and G.3 (n=1) astrocytoma samples (C) whereas specific regions (1 and 2) within the GBM tissue revealed robust expression of IGFBP2 protein (D). Also shown are tumor blood vessels (Pecam1, red), Ast-like cells (GFAP, cyan) and DAPI-positive cell nuclei (blue). Note the spatial localization pattern of IGFBP2-positive TCs in pseudopalisading region 1 (arrows, top left panel) in the GBM sample (n=1). Magnified images (right) are indicated by boxed areas. Region 2 (bottom panel) reveals IGFBP2$^{+}$ TCs and GFAP$^{+}$ cells (GFAP, cyan, arrows in bottom right panel). Also shown are Pecam1$^{+}$ tumor blood vessels (red) and DAPI-positive cell nuclei (blue). Scale bars: 100 $\mu$m and 25 $\mu$m (magnified images).

**Supplemental Figure 13. Analysis of Netrin 4 protein expression in human brain tumor patient samples. (A-C);** Representative images of G.2 (A) and G.3 (B) astrocytoma samples, and higher G.4 GBM samples (C) showing NTN4/netrin 4 (red) and the MG/MØ marker AIF1 (green). Note the increased expression of netrin 4 protein in ECs and some TCs in both G.2 (n=2) and G.3 (n=2) astrocytoma samples in comparison to G.4 GBM samples (n=2), which express lower levels of netrin 4 protein. Also note the varying population of tumor infiltrating

AIF1+ MG/MØ cells across patient samples. Bottom panels show magnified images of boxed areas in upper panels.

**Supplemental Figure 14. Analysis of Annexin A1 protein expression in human brain tumor patient samples. (A);** Representative images of LE/IT regions in GBM samples (n=3) showing expression of ANXA1/annexin-a1 (green, top panel) and GFAP (cyan, bottom panel). Note the heterogeneous levels of annexin-a1 protein expression, indicative of varying degrees of tumor infiltration in the different patient samples. These samples also show different levels of GFAP protein expression in regions of the tumor infiltrating brain parenchyma (bottom panel). Also shown are tumor endothelial cells (Pecam1, red) and DAPI-positive cell nuclei (blue). Scale bars: 100 $\mu$m. **(B);** Representative images showing annexin-a1 protein expression in G.2 and G.3 astrocytomas, as well as G.4 GBM samples. Annexin-a1 protein expression is significantly upregulated in both TC and Ast-like cells (indicated by arrows) in G.4 GBM samples (n=2) versus lower grade G.2 (n=2) and G.3 (n=2) astrocytoma samples. Also shown are tumor blood vessels (Pecam1, red) and DAPI-positive cell nuclei (blue). Scale bars: 25 $\mu$m.

**Supplemental Table 1. Details of brain tumor patients with tumor grades and IDH mutational status of samples used for in situ single-cell spatial transcriptomic profiling of tumor matrisome gene expression patterns and related cell markers.**
Information for IDH mutational status is unavailable for two grade 2 and three grade 3 astrocytoma samples (*). Another sample from grade 2 astrocytoma is IDH2-mutant (**) whereas one grade 4 GBM sample is IDH1-mutant (***).
Patient/tumor-matched non-cancerous/normal samples indicated.
TMA: tumor microarray, LE/IT: leading edge/infiltrating tumor.

| Tissue Sample Group | Sample ID | Sex | Age (yrs.) | IDH Status | Matched normal samples |
|---|---|---|---|---|---|
| Grade 3 astrocytoma | G3-3.0 | F | 33 | Mut | |
| GBM | GBM-4.0 | F | 54 | WT | |
| TMA 4 GBM (LE/IT) | GBM-LE/IT-01 | M | 75 | WT | Normal-1 |
| TMA 4 GBM (LE/IT) | GBM-LE/IT-02 | F | 46 | WT | Normal-2 |
| TMA 4 GBM (LE/IT) | GBM-LE/IT-03 | F | 71 | WT | Normal-3 |
| TMA 6 Grade 2 | G.2.1 | F | 39 | IDH1 Mut | |
| TMA 6 Grade 2 | G.2.2 | M | 46 | * | |
| TMA 6 Grade 2 | G.2.3 | M | 59 | IDH1 Mut | |
| TMA 6 Grade 2 | G.2.4 | M | 35 | IDH1 Mut | |
| TMA 6 Grade 2 | G.2.6 | M | 27 | IDH1 Mut | |
| TMA 6 Grade 2 | G.2.7 | F | 27 | IDH1 Mut | |
| TMA 6 Grade 2 | G.2.8 | M | 58 | IDH1 Mut | |
| TMA 6 Grade 2 | G.2.9 | F | 26 | IDH2 Mut ** | |
| TMA 6 Grade 2 | G.2.10 | F | 32 | IDH1 Mut | Normal-1 |
| TMA 6 Grade 3 | G.3.1 | F | 27 | IDH1 Mut | |
| TMA 6 Grade 3 | G.3.2 | M | 55 | IDH1 Mut | |
| TMA 6 Grade 3 | G.3.3 | F | 79 | IDH1 Mut | |
| TMA 6 Grade 3 | G.3.4 | F | 53 | IDH1 Mut | |
| TMA 6 Grade 3 | G.3.5 | F | 46 | IDH1 Mut | |
| TMA 6 Grade 3 | G.3.6 | M | 28 | IDH1 Mut | |
| TMA 6 Grade 3 | G.3.7 | M | 55 | IDH1 Mut | |
| TMA 6 Grade 3 | G.3.8 | F | 43 | IDH1 Mut | |
| TMA 6 Grade 3 | G.3.9 | M | 33 | IDH1 Mut | Normal-2 |
| TMA 6 Grade 3 | G.3.10 | M | 52 | IDH1 Mut | Normal-3 |
| TMA 6 Grade 4 | G.4.1 | F | 72 | WT | |
| TMA 6 Grade 4 | G.4.4 | M | 65 | WT | Normal-5 |
| TMA 6 Grade 4 | G.4.5 | F | 57 | WT | |
| TMA 6 Grade 4 | G.4.6 | M | 72 | WT | |
| TMA 6 Grade 4 | G.4.7 | M | 61 | WT | |
| TMA 6 Grade 4 | G.4.8 | M | 47 | WT | |
| TMA 6 Grade 4 | G.4.9 | M | 66 | WT | |
| TMA 2 Grade 2 | G.2-P10 | M | 46 | * | |
| TMA 2 Grade 2 | G.2-P11 | M | 43 | IDH1 Mut | |
| TMA 2 Grade 3 | G.3-P14 | F | 46 | * | |
| TMA 2 Grade 3 | G.3-P13 | M | 36 | * | |
| TMA 2 Grade 3 | G.3-P12 | F | 32 | * | |
| TMA 2 Grade 4 | G.4-P13 | F | 61 | WT | |
| TMA 2 Grade 4 | G.4-P14 | F | 42 | IDH1 Mut*** | |
| TMA 2 Grade 4 | G.4-P12 | M | 74 | WT | |
| GBM | G.4-P15 | F | 55 | WT | |
| Grade 2 astrocytoma | G.2-P12 | M | 43 | IDH1 Mut | |
| GBM | G.4-P16 | M | 45 | WT | |

**Supplemental Table 2. Details of the matrisome probe panel.**

Information for all genes used to custom-design the probe panel from 10x Genomics for Xenium *in situ* single cell spatial detection and analysis.

Gene categories representing human core matrisome, ECM-affiliated and cellular markers have been differentially color-coded.

| Gene | Ensembl_ID | Category |
|---|---|---|
| ABI3BP | ENSG00000154175 | ECM glycoproteins |
| ADIPOQ | ENSG00000181092 | ECM glycoproteins |
| AEBP1 | ENSG00000106624 | ECM glycoproteins |
| AGRN | ENSG00000188157 | ECM glycoproteins |
| AMELX | ENSG00000125363 | ECM glycoproteins |
| BMPER | ENSG00000164619 | ECM glycoproteins |
| CRELD1 | ENSG00000163703 | ECM glycoproteins |
| CRELD2 | ENSG00000184164 | ECM glycoproteins |
| CRISPLD1 | ENSG00000121005 | ECM glycoproteins |
| CRISPLD2 | ENSG00000103196 | ECM glycoproteins |
| CCN2 | ENSG00000118523 | ECM glycoproteins |
| CYR61 | ENSG00000142871 | ECM glycoproteins |
| DMBT1 | ENSG00000187908 | ECM glycoproteins |
| DPT | ENSG00000143196 | ECM glycoproteins |
| ECM1 | ENSG00000143369 | ECM glycoproteins |
| ECM2 | ENSG00000106823 | ECM glycoproteins |
| EDIL3 | ENSG00000164176 | ECM glycoproteins |
| EFEMP1 | ENSG00000115380 | ECM glycoproteins |
| EFEMP2 | ENSG00000172638 | ECM glycoproteins |
| EGFLAM | ENSG00000164318 | ECM glycoproteins |
| ELN | ENSG00000049540 | ECM glycoproteins |
| FBLN2 | ENSG00000163520 | ECM glycoproteins |
| FBLN5 | ENSG00000140092 | ECM glycoproteins |
| FBLN7 | ENSG00000144152 | ECM glycoproteins |
| FBN1 | ENSG00000166147 | ECM glycoproteins |
| FBN2 | ENSG00000138829 | ECM glycoproteins |
| FBN3 | ENSG00000142449 | ECM glycoproteins |
| FGL1 | ENSG00000104760 | ECM glycoproteins |
| FGL2 | ENSG00000127951 | ECM glycoproteins |
| FN1 | ENSG00000115414 | ECM glycoproteins |
| FNDC1 | ENSG00000164694 | ECM glycoproteins |
| FNDC7 | ENSG00000143107 | ECM glycoproteins |
| FNDC8 | ENSG00000073598 | ECM glycoproteins |
| FRAS1 | ENSG00000138759 | ECM glycoproteins |

| | | |
|---|---|---|
| GAS6 | ENSG00000183087 | ECM glycoproteins |
| IBSP | ENSG00000029559 | ECM glycoproteins |
| IGFALS | ENSG00000099769 | ECM glycoproteins |
| IGFBP1 | ENSG00000146678 | ECM glycoproteins |
| IGFBP2 | ENSG00000115457 | ECM glycoproteins |
| IGFBP6 | ENSG00000167779 | ECM glycoproteins |
| IGFBP7 | ENSG00000163453 | ECM glycoproteins |
| IGFBPL1 | ENSG00000137142 | ECM glycoproteins |
| IGSF10 | ENSG00000152580 | ECM glycoproteins |
| LAMA1 | ENSG00000101680 | ECM glycoproteins |
| LAMA3 | ENSG00000053747 | ECM glycoproteins |
| LAMA4 | ENSG00000112769 | ECM glycoproteins |
| LAMA5 | ENSG00000130702 | ECM glycoproteins |
| LAMB1 | ENSG00000091136 | ECM glycoproteins |
| LAMB2 | ENSG00000172037 | ECM glycoproteins |
| LAMB3 | ENSG00000196878 | ECM glycoproteins |
| LAMB4 | ENSG00000091128 | ECM glycoproteins |
| LAMC1 | ENSG00000135862 | ECM glycoproteins |
| LAMC2 | ENSG00000058085 | ECM glycoproteins |
| LAMC3 | ENSG00000050555 | ECM glycoproteins |
| LGI1 | ENSG00000108231 | ECM glycoproteins |
| LGI2 | ENSG00000153012 | ECM glycoproteins |
| LGI3 | ENSG00000168481 | ECM glycoproteins |
| LGI4 | ENSG00000153902 | ECM glycoproteins |
| LRG1 | ENSG00000171236 | ECM glycoproteins |
| LTBP1 | ENSG00000049323 | ECM glycoproteins |
| LTBP2 | ENSG00000119681 | ECM glycoproteins |
| LTBP3 | ENSG00000168056 | ECM glycoproteins |
| LTBP4 | ENSG00000090006 | ECM glycoproteins |
| MATN2 | ENSG00000132561 | ECM glycoproteins |
| MATN3 | ENSG00000132031 | ECM glycoproteins |
| MFGE8 | ENSG00000140545 | ECM glycoproteins |
| MGP | ENSG00000111341 | ECM glycoproteins |
| MMRN1 | ENSG00000138722 | ECM glycoproteins |
| MMRN2 | ENSG00000173269 | ECM glycoproteins |
| MXRA5 | ENSG00000101825 | ECM glycoproteins |
| NDNF | ENSG00000173376 | ECM glycoproteins |
| NID1 | ENSG00000116962 | ECM glycoproteins |
| NOV | ENSG00000136999 | ECM glycoproteins |
| NTN1 | ENSG00000065320 | ECM glycoproteins |
| NTN3 | ENSG00000162068 | ECM glycoproteins |

| | | |
|---|---|---|
| NTN4 | ENSG00000074527 | ECM glycoproteins |
| NTN5 | ENSG00000142233 | ECM glycoproteins |
| PAPLN | ENSG00000100767 | ECM glycoproteins |
| POSTN | ENSG00000133110 | ECM glycoproteins |
| RELN | ENSG00000189056 | ECM glycoproteins |
| RSPO1 | ENSG00000169218 | ECM glycoproteins |
| RSPO2 | ENSG00000147655 | ECM glycoproteins |
| RSPO3 | ENSG00000146374 | ECM glycoproteins |
| RSPO4 | ENSG00000101282 | ECM glycoproteins |
| SLIT1 | ENSG00000187122 | ECM glycoproteins |
| SLIT2 | ENSG00000145147 | ECM glycoproteins |
| SLIT3 | ENSG00000184347 | ECM glycoproteins |
| SMOC1 | ENSG00000198732 | ECM glycoproteins |
| SNED1 | ENSG00000162804 | ECM glycoproteins |
| SPARC | ENSG00000113140 | ECM glycoproteins |
| SPARCL1 | ENSG00000152583 | ECM glycoproteins |
| SPON1 | ENSG00000262655 | ECM glycoproteins |
| SPON2 | ENSG00000159674 | ECM glycoproteins |
| SPP1 | ENSG00000118785 | ECM glycoproteins |
| SVEP1 | ENSG00000165124 | ECM glycoproteins |
| TGFBI | ENSG00000120708 | ECM glycoproteins |
| THBS1 | ENSG00000137801 | ECM glycoproteins |
| THBS2 | ENSG00000186340 | ECM glycoproteins |
| THBS3 | ENSG00000169231 | ECM glycoproteins |
| THBS4 | ENSG00000113296 | ECM glycoproteins |
| TNR | ENSG00000116147 | ECM glycoproteins |
| TNXB | ENSG00000168477 | ECM glycoproteins |
| VTN | ENSG00000109072 | ECM glycoproteins |
| VWF | ENSG00000110799 | ECM glycoproteins |
| | | |
| COL10A1 | ENSG00000123500 | Collagens |
| COL11A1 | ENSG00000060718 | Collagens |
| COL11A2 | ENSG00000204248 | Collagens |
| COL16A1 | ENSG00000084636 | Collagens |
| COL17A1 | ENSG00000065618 | Collagens |
| COL20A1 | ENSG00000101203 | Collagens |
| COL21A1 | ENSG00000124749 | Collagens |
| COL23A1 | ENSG00000050767 | Collagens |
| COL24A1 | ENSG00000171502 | Collagens |
| COL26A1 | ENSG00000160963 | Collagens |
| COL27A1 | ENSG00000196739 | Collagens |

| | | |
|---|---|---|
| COL28A1 | ENSG00000215018 | Collagens |
| COL2A1 | ENSG00000139219 | Collagens |
| COL7A1 | ENSG00000114270 | Collagens |
| COL8A1 | ENSG00000144810 | Collagens |
| COL8A2 | ENSG00000171812 | Collagens |
| COL9A1 | ENSG00000112280 | Collagens |
| COL9A2 | ENSG00000049089 | Collagens |
| COL9A3 | ENSG00000092758 | Collagens |
| | | |
| ACAN | ENSG00000157766 | Proteoglycans |
| ASPN | ENSG00000106819 | Proteoglycans |
| BGN | ENSG00000182492 | Proteoglycans |
| CHAD | ENSG00000136457 | Proteoglycans |
| ESM1 | ENSG00000164283 | Proteoglycans |
| FMOD | ENSG00000122176 | Proteoglycans |
| HAPLN1 | ENSG00000145681 | Proteoglycans |
| HAPLN2 | ENSG00000132702 | Proteoglycans |
| HAPLN3 | ENSG00000140511 | Proteoglycans |
| HAPLN4 | ENSG00000187664 | Proteoglycans |
| HSPG2 | ENSG00000142798 | Proteoglycans |
| IMPG1 | ENSG00000112706 | Proteoglycans |
| IMPG2 | ENSG00000081148 | Proteoglycans |
| LUM | ENSG00000139329 | Proteoglycans |
| NCAN | ENSG00000130287 | Proteoglycans |
| OGN | ENSG00000106809 | Proteoglycans |
| OMD | ENSG00000127083 | Proteoglycans |
| OPTC | ENSG00000188770 | Proteoglycans |
| PODN | ENSG00000174348 | Proteoglycans |
| PODNL1 | ENSG00000132000 | Proteoglycans |
| PRG4 | ENSG00000116690 | Proteoglycans |
| SPOCK1 | ENSG00000152377 | Proteoglycans |
| SPOCK2 | ENSG00000107742 | Proteoglycans |
| SPOCK3 | ENSG00000196104 | Proteoglycans |
| SRGN | ENSG00000122862 | Proteoglycans |
| | | |
| FREM1 | ENSG00000164946 | ECM-affliated proteins |
| FREM2 | ENSG00000150893 | ECM-affliated proteins |
| FREM3 | ENSG00000183090 | ECM-affliated proteins |
| ELFN1 | ENSG00000225968 | ECM-affliated proteins |
| ELFN2 | ENSG00000166897 | ECM-affliated proteins |
| ELFN2 | ENSG00000243902 | ECM-affliated proteins |

| | | |
|---|---|---|
| EMCN | ENSG00000164035 | ECM-affliated proteins |
| FCN1 | ENSG00000085265 | ECM-affliated proteins |
| FCN2 | ENSG00000160339 | ECM-affliated proteins |
| FCN3 | ENSG00000142748 | ECM-affliated proteins |
| SDC1 | ENSG00000115884 | ECM-affliated proteins |
| SDC2 | ENSG00000169439 | ECM-affliated proteins |
| SDC3 | ENSG00000162512 | ECM-affliated proteins |
| SDC4 | ENSG00000124145 | ECM-affliated proteins |
| SEMA3A | ENSG00000075213 | ECM-affliated proteins |
| SEMA3B | ENSG00000012171 | ECM-affliated proteins |
| SEMA3C | ENSG00000075223 | ECM-affliated proteins |
| SEMA3D | ENSG00000153993 | ECM-affliated proteins |
| SEMA3E | ENSG00000170381 | ECM-affliated proteins |
| SEMA3F | ENSG00000001617 | ECM-affliated proteins |
| SEMA3G | ENSG00000010319 | ECM-affliated proteins |
| SEMA4A | ENSG00000196189 | ECM-affliated proteins |
| SEMA4B | ENSG00000185033 | ECM-affliated proteins |
| SEMA4C | ENSG00000168758 | ECM-affliated proteins |
| SEMA4D | ENSG00000187764 | ECM-affliated proteins |
| SEMA4F | ENSG00000135622 | ECM-affliated proteins |
| SEMA4G | ENSG00000095539 | ECM-affliated proteins |
| SEMA5A | ENSG00000112902 | ECM-affliated proteins |
| SEMA5B | ENSG00000082684 | ECM-affliated proteins |
| SEMA6A | ENSG00000092421 | ECM-affliated proteins |
| SEMA6B | ENSG00000167680 | ECM-affliated proteins |
| SEMA6C | ENSG00000143434 | ECM-affliated proteins |
| SEMA6D | ENSG00000137872 | ECM-affliated proteins |
| SEMA7A | ENSG00000138623 | ECM-affliated proteins |
| PLXDC1 | ENSG00000161381 | ECM-affliated proteins |
| PLXDC2 | ENSG00000120594 | ECM-affliated proteins |
| PLXNA1 | ENSG00000114554 | ECM-affliated proteins |
| PLXNA2 | ENSG00000076356 | ECM-affliated proteins |
| PLXNA3 | ENSG00000130827 | ECM-affliated proteins |
| PLXNA4 | ENSG00000221866 | ECM-affliated proteins |
| PLXNB1 | ENSG00000164050 | ECM-affliated proteins |
| PLXNB2 | ENSG00000196576 | ECM-affliated proteins |
| PLXNB3 | ENSG00000198753 | ECM-affliated proteins |
| PLXNC1 | ENSG00000136040 | ECM-affliated proteins |
| PLXND1 | ENSG00000004399 | ECM-affliated proteins |
| MUC1 | ENSG00000185499 | ECM-affliated proteins |
| MUC12 | ENSG00000205277 | ECM-affliated proteins |

| | | |
|---|---|---|
| MUC13 | ENSG00000173702 | ECM-affliated proteins |
| MUC15 | ENSG00000169550 | ECM-affliated proteins |
| MUC16 | ENSG00000181143 | ECM-affliated proteins |
| MUC17 | ENSG00000169876 | ECM-affliated proteins |
| MUC19 | ENSG00000205592 | ECM-affliated proteins |
| MUC2 | ENSG00000198788 | ECM-affliated proteins |
| MUC20 | ENSG00000176945 | ECM-affliated proteins |
| MUC21 | ENSG00000204544 | ECM-affliated proteins |
| MUC22 | ENSG00000261272 | ECM-affliated proteins |
| MUC3A | ENSG00000169894 | ECM-affliated proteins |
| MUC4 | ENSG00000145113 | ECM-affliated proteins |
| MUC5AC | ENSG00000215182 | ECM-affliated proteins |
| MUC5B | ENSG00000117983 | ECM-affliated proteins |
| MUC6 | ENSG00000184956 | ECM-affliated proteins |
| MUC7 | ENSG00000171195 | ECM-affliated proteins |
| MUCL1 | ENSG00000172551 | ECM-affliated proteins |
| GPC1 | ENSG00000063660 | ECM-affliated proteins |
| GPC2 | ENSG00000213420 | ECM-affliated proteins |
| GPC3 | ENSG00000147257 | ECM-affliated proteins |
| GPC4 | ENSG00000076716 | ECM-affliated proteins |
| GPC5 | ENSG00000179399 | ECM-affliated proteins |
| GPC6 | ENSG00000183098 | ECM-affliated proteins |
| SFTA2 | ENSG00000196260 | ECM-affliated proteins |
| SFTA3 | ENSG00000229415 | ECM-affliated proteins |
| SFTPA1 | ENSG00000122852 | ECM-affliated proteins |
| SFTPA2 | ENSG00000185303 | ECM-affliated proteins |
| SFTPB | ENSG00000168878 | ECM-affliated proteins |
| SFTPC | ENSG00000168484 | ECM-affliated proteins |
| SFTPD | ENSG00000133661 | ECM-affliated proteins |
| CD209 | ENSG00000090659 | ECM-affliated proteins |
| CLC | ENSG00000105205 | ECM-affliated proteins |
| CLEC10A | ENSG00000132514 | ECM-affliated proteins |
| CLEC12A | ENSG00000172322 | ECM-affliated proteins |
| CLEC12B | ENSG00000256660 | ECM-affliated proteins |
| CLEC14A | ENSG00000176435 | ECM-affliated proteins |
| CLEC17A | ENSG00000187912 | ECM-affliated proteins |
| CLEC18A | ENSG00000157322 | ECM-affliated proteins |
| CLEC18B | ENSG00000140839 | ECM-affliated proteins |
| CLEC18C | ENSG00000157335 | ECM-affliated proteins |
| CLEC19A | ENSG00000261210 | ECM-affliated proteins |
| CLEC1A | ENSG00000150048 | ECM-affliated proteins |

| | | |
|---|---|---|
| CLEC1B | ENSG00000165682 | ECM-affliated proteins |
| CLEC2A | ENSG00000188393 | ECM-affliated proteins |
| CLEC2B | ENSG00000110852 | ECM-affliated proteins |
| CLEC2D | ENSG00000069493 | ECM-affliated proteins |
| CLEC2L | ENSG00000236279 | ECM-affliated proteins |
| CLEC3A | ENSG00000166509 | ECM-affliated proteins |
| CLEC3B | ENSG00000163815 | ECM-affliated proteins |
| CLEC4A | ENSG00000111729 | ECM-affliated proteins |
| CLEC4C | ENSG00000198178 | ECM-affliated proteins |
| CLEC4D | ENSG00000166527 | ECM-affliated proteins |
| CLEC4E | ENSG00000166523 | ECM-affliated proteins |
| CLEC4F | ENSG00000152672 | ECM-affliated proteins |
| CLEC4G | ENSG00000182566 | ECM-affliated proteins |
| CLEC4M | ENSG00000104938 | ECM-affliated proteins |
| CLEC5A | ENSG00000258227 | ECM-affliated proteins |
| CLEC6A | ENSG00000205846 | ECM-affliated proteins |
| CLEC7A | ENSG00000172243 | ECM-affliated proteins |
| CLEC9A | ENSG00000197992 | ECM-affliated proteins |
| COLEC10 | ENSG00000184374 | ECM-affliated proteins |
| COLEC11 | ENSG00000118004 | ECM-affliated proteins |
| COLEC12 | ENSG00000158270 | ECM-affliated proteins |
| CSPG4 | ENSG00000173546 | ECM-affliated proteins |
| CSPG5 | ENSG00000114646 | ECM-affliated proteins |
| C1QA | ENSG00000173372 | ECM-affliated proteins |
| C1QB | ENSG00000173369 | ECM-affliated proteins |
| C1QC | ENSG00000159189 | ECM-affliated proteins |
| C1QL1 | ENSG00000131094 | ECM-affliated proteins |
| C1QL2 | ENSG00000144119 | ECM-affliated proteins |
| C1QL3 | ENSG00000165985 | ECM-affliated proteins |
| C1QL4 | ENSG00000186897 | ECM-affliated proteins |
| C1QTNF1 | ENSG00000173918 | ECM-affliated proteins |
| C1QTNF2 | ENSG00000145861 | ECM-affliated proteins |
| C1QTNF3 | ENSG00000082196 | ECM-affliated proteins |
| C1QTNF5 | ENSG00000223953 | ECM-affliated proteins |
| C1QTNF6 | ENSG00000133466 | ECM-affliated proteins |
| C1QTNF7 | ENSG00000163145 | ECM-affliated proteins |
| C1QTNF8 | ENSG00000184471 | ECM-affliated proteins |
| C1QTNF9 | ENSG00000240654 | ECM-affliated proteins |
| ANXA1 | ENSG00000135046 | ECM-affliated proteins |
| ANXA10 | ENSG00000109511 | ECM-affliated proteins |
| ANXA11 | ENSG00000122359 | ECM-affliated proteins |

| | | |
|---|---|---|
| ANXA13 | ENSG00000104537 | ECM-affliated proteins |
| ANXA2 | ENSG00000182718 | ECM-affliated proteins |
| ANXA3 | ENSG00000138772 | ECM-affliated proteins |
| ANXA4 | ENSG00000196975 | ECM-affliated proteins |
| ANXA5 | ENSG00000164111 | ECM-affliated proteins |
| ANXA6 | ENSG00000197043 | ECM-affliated proteins |
| ANXA7 | ENSG00000138279 | ECM-affliated proteins |
| ANXA8 | ENSG00000265190 | ECM-affliated proteins |
| ANXA8L1 | ENSG00000264230 | ECM-affliated proteins |
| ANXA9 | ENSG00000143412 | ECM-affliated proteins |
| GREM1 | ENSG00000166923 | ECM-affliated proteins |
| HPX | ENSG00000110169 | ECM-affliated proteins |
| LGALSL | ENSG00000119862 | ECM-affliated proteins |
| ITLN1 | ENSG00000179914 | ECM-affliated proteins |
| ITLN2 | ENSG00000158764 | ECM-affliated proteins |
| LGALS1 | ENSG00000100097 | ECM-affliated proteins |
| LGALS12 | ENSG00000133317 | ECM-affliated proteins |
| LGALS13 | ENSG00000105198 | ECM-affliated proteins |
| LGALS14 | ENSG00000006659 | ECM-affliated proteins |
| LGALS16 | ENSG00000249861 | ECM-affliated proteins |
| LGALS2 | ENSG00000100079 | ECM-affliated proteins |
| LGALS3 | ENSG00000131981 | ECM-affliated proteins |
| LGALS4 | ENSG00000171747 | ECM-affliated proteins |
| LGALS8 | ENSG00000116977 | ECM-affliated proteins |
| LGALS9 | ENSG00000168961 | ECM-affliated proteins |
| LGALS9B | ENSG00000170298 | ECM-affliated proteins |
| LGALS9C | ENSG00000171916 | ECM-affliated proteins |
| LMAN1 | ENSG00000074695 | ECM-affliated proteins |
| LMAN1L | ENSG00000140506 | ECM-affliated proteins |
| MBL2 | ENSG00000165471 | ECM-affliated proteins |
| OVGP1 | ENSG00000085465 | ECM-affliated proteins |
| PARM1 | ENSG00000169116 | ECM-affliated proteins |
| OPRPN | ENSG00000171199 | ECM-affliated proteins |
| REG1A | ENSG00000115386 | ECM-affliated proteins |
| REG1B | ENSG00000172023 | ECM-affliated proteins |
| REG3A | ENSG00000172016 | ECM-affliated proteins |
| REG3G | ENSG00000143954 | ECM-affliated proteins |
| REG4 | ENSG00000134193 | ECM-affliated proteins |
| | | |
| ITGAD | ENSG00000156886 | Integrins |
| ITGAE | ENSG00000083457 | Integrins |

| | | |
|---|---|---|
| ITGAL | ENSG00000005844 | Integrins |
| ITGAV | ENSG00000138448 | Integrins |
| ITGA1 | ENSG00000213949 | Integrins |
| ITGA2 | ENSG00000164171 | Integrins |
| ITGA2B | ENSG00000005961 | Integrins |
| ITGA3 | ENSG00000005884 | Integrins |
| ITGA4 | ENSG00000115232 | Integrins |
| ITGA5 | ENSG00000161638 | Integrins |
| ITGA6 | ENSG00000091409 | Integrins |
| ITGA7 | ENSG00000135424 | Integrins |
| ITGA8 | ENSG00000077943 | Integrins |
| ITGA9 | ENSG00000144668 | Integrins |
| ITGA10 | ENSG00000143127 | Integrins |
| ITGA11 | ENSG00000137809 | Integrins |
| ITGB1 | ENSG00000150093 | Integrins |
| ITGB3 | ENSG00000259207 | Integrins |
| ITGB4 | ENSG00000132470 | Integrins |
| ITGB5 | ENSG00000082781 | Integrins |
| ITGB6 | ENSG00000115221 | Integrins |
| ITGB7 | ENSG00000139626 | Integrins |
| ITGB8 | ENSG00000105855 | Integrins |
| | | |
| PECAM1 | ENSG00000261371 | Endothelial |
| KDR | ENSG00000128052 | Endothelial |
| NRP1 | ENSG00000099250 | Endothelial |
| CD34 | ENSG00000174059 | Endothelial |
| AQP4 | ENSG00000171885 | Astrocyte |
| MLC1 | ENSG00000100427 | Astrocyte |
| GFAP | ENSG00000131095 | Astrocyte |
| ALDH1L1 | ENSG00000144908 | Astrocyte |
| HEPACAM | ENSG00000165478 | Astrocyte |
| RBFOX3 | ENSG00000167281 | Neural |
| CALB1 | ENSG00000104327 | Neural |
| CALB2 | ENSG00000172137 | Neural |
| S100G | ENSG00000169906 | OPC |
| SCGN | ENSG00000079689 | Neural |
| NEFL | ENSG00000277586 | Neural |
| NEFH | ENSG00000100285 | Neural |
| NEFM | ENSG00000104722 | Neural |
| AIF1 | ENSG00000204472 | Microglia |
| CD68 | ENSG00000129226 | Microglia |

| | | |
|---|---|---|
| TMEM119 | ENSG00000183160 | Microglia |
| OLIG2 | ENSG00000205927 | OPC |
| SOX10 | ENSG00000100146 | Oligodendrocytes |
| MBP | ENSG00000197971 | Oligodendrocytes |
| VIM | ENSG00000026025 | GBM |
| EGFR | ENSG00000146648 | GBM |
| IDH1 | ENSG00000138413 | GBM |
| IDH2 | ENSG00000182054 | GBM |
| NES | ENSG00000132688 | GSC |
| SOX2 | ENSG00000181449 | GSC |
| GRIP1 | ENSG00000155974 | Neural |
| GRIP2 | ENSG00000144596 | Neural |
| NPNT | ENSG00000168743 | Neural |
| | | |
| | | |
| CUX2 | ENSG00000111249 | Neural |
| TESPA1 | ENSG00000135426 | Neural |
| ZBBX | ENSG00000169064 | Neural |
| ADAMTS16 | ENSG00000145536 | Neural |
| FSTL4 | ENSG00000053108 | Neural |
| OTOGL | ENSG00000165899 | Neural |
| POU6F2 | ENSG00000106536 | Neural |
| RORB | ENSG00000198963 | Neural |
| SLC17A6 | ENSG00000091664 | Neural |
| PCSK1 | ENSG00000175426 | Neural |
| RIT2 | ENSG00000152214 | Neural |
| SNCG | ENSG00000173267 | Neural |
| SORCS1 | ENSG00000108018 | Neural |
| HTR2A | ENSG00000102468 | Neural |
| RXFP1 | ENSG00000171509 | Neural |
| ATP2C2 | ENSG00000064270 | Neural |
| CD36 | ENSG00000135218 | Neural |
| CRYM | ENSG00000103316 | Neural |
| HTR2C | ENSG00000147246 | Neural |
| TPH2 | ENSG00000139287 | Neural |
| TSHZ2 | ENSG00000182463 | Neural |
| VWC2L | ENSG00000174453 | Neural |
| FILIP1 | ENSG00000118407 | Neural |
| HS3ST2 | ENSG00000122254 | Neural |
| HS3ST4 | ENSG00000182601 | Neural |
| KCNH5 | ENSG00000140015 | Neural |

| | | |
|---|---|---|
| ADAMTS3 | ENSG00000156140 | Neural |
| SNTB2 | ENSG00000168807 | Neural |
| CDH12 | ENSG00000154162 | Neural |
| GAS2L3 | ENSG00000139354 | Neural |
| NPY1R | ENSG00000164128 | Neural |
| NTNG2 | ENSG00000196358 | Neural |
| NWD2 | ENSG00000174145 | Neural |
| RASGRP1 | ENSG00000172575 | Neural |
| SLC26A4 | ENSG00000091137 | Neural |
| THEMIS | ENSG00000172673 | Neural |
| TRPC5 | ENSG00000072315 | Neural |
| ZDHHC23 | ENSG00000184307 | Neural |
| NPFFR2 | ENSG00000056291 | Neural |
| ROS1 | ENSG00000047936 | Neural |
| SLC17A7 | ENSG00000104888 | Neural |
| DNER | ENSG00000187957 | Neural |
| PTCHD4 | ENSG00000244694 | Neural |
| ADRA1A | ENSG00000120907 | Neural |
| ADRA1B | ENSG00000170214 | Neural |
| LYPD6B | ENSG00000150556 | Neural |
| PROX1 | ENSG00000117707 | Neural |
| THSD7B | ENSG00000144229 | Neural |
| COL25A1 | ENSG00000188517 | Neural |
| PLCH1 | ENSG00000114805 | Neural |
| SYNPR | ENSG00000163630 | Neural |
| TRHDE | ENSG00000072657 | Neural |
| SLC24A3 | ENSG00000185052 | Neural |
| VIP | ENSG00000146469 | Neural |
| NLGN3 | ENSG00000196338 | Neural |
| BDNF | ENSG00000176697 | Neural |
| GRP78 | ENSG00000044574 | Neural |
| ADAM10 | ENSG00000137845 | Neural |
| IGF-1 | ENSG00000017427 | Neural |
| NTRK2 | ENSG00000148053 | Neural |
| PIK3CA | ENSG00000121879 | Neural |
| GAP43 | ENSG00000172020 | Neural |
| | | |
| | | |
| B4GALNT1 | ENSG00000135454 | GBM |
| BCAN | ENSG00000132692 | GBM |
| CAV1 | ENSG00000105974 | GBM |

| | | |
|---|---|---|
| CHODL | ENSG00000154645 | GBM |
| ELOVL2 | ENSG00000197977 | GBM |
| HES1 | ENSG00000114315 | GBM |
| HILPDA | ENSG00000135245 | GBM |
| IGFBP3 | ENSG00000146674 | GBM |
| IGFBP5 | ENSG00000115461 | GBM |
| LOX | ENSG00000113083 | GBM |
| MGST1 | ENSG00000008394 | GBM |
| NNAT | ENSG00000053438 | GBM |
| PSEN2 | ENSG00000143801 | GBM |
| PSENEN | ENSG00000205155 | GBM |
| SOX11 | ENSG00000176887 | GBM |
| SOX4 | ENSG00000124766 | GBM |
| TP53 | ENSG00000141510 | GBM |
| TRIL | ENSG00000255690 | GBM |
| TTYH1 | ENSG00000167614 | GBM |
| | | |
| APOE | ENSG00000130203 | Microglia-PVM |
| ARHGAP24 | ENSG00000138639 | Microglia-PVM |
| CCL4 | ENSG00000275302 | Microglia-PVM |
| CD86 | ENSG00000114013 | Microglia-PVM |
| CTSH | ENSG00000103811 | Microglia-PVM |
| FASLG | ENSG00000117560 | Microglia-PVM |
| HLA-DQA1 | ENSG00000196735 | Microglia-PVM |
| IFITM3 | ENSG00000142089 | Microglia-PVM |
| ITGAM | ENSG00000169896 | Microglia-PVM |
| ITGAX | ENSG00000140678 | Microglia-PVM |
| LRRK1 | ENSG00000154237 | Microglia-PVM |
| LYVE1 | ENSG00000133800 | Microglia-PVM |
| P2RY12 | ENSG00000169313 | Microglia-PVM |
| P2RY13 | ENSG00000181631 | Microglia-PVM |
| PTPRC | ENSG00000081237 | Microglia-PVM |
| RGS16 | ENSG00000143333 | Microglia-PVM |
| SPI1 | ENSG00000066336 | Microglia-PVM |
| TGFB1 | ENSG00000105329 | Microglia-PVM |
| TREM2 | ENSG00000095970 | Microglia-PVM |
| | | |
| MKI67 | ENSG00000148773 | Proliferation |
| PCNA | ENSG00000132646 | Proliferation |
| CCNA1 | ENSG00000133101 | Proliferation |
| CCNB2 | ENSG00000157456 | Proliferation |

| | | |
|---|---|---|
| CDK1 | ENSG00000170312 | Proliferation |
| CENPF | ENSG00000117724 | Proliferation |
| KIT | ENSG00000157404 | Proliferation |
| TOP2A | ENSG00000131747 | Proliferation |
