# Supplementary material for: Single cell spatial profiling of the matrisome identifies region-specific adhesion and signaling networks in glioblastoma
Source: Commun Biol. 2025 Dec 9;9:17. doi: 10.1038/s42003-025-09270-7 (PMC12769532; doi:10.1038/s42003-025-09270-7)
Supplement: Supplementary file 2 — Reporting Summary [file 42003_2025_9270_MOESM2_ESM.pdf]

nature portfolio

Corresponding author(s): *Double-anonymous peer review submissions: write DAPR and your manuscript number here instead of author names.*

Last updated by author(s): *YYYY-MM-DD*

Reporting Summary

Nature Portfolio wishes to improve the reproducibility of the work that we publish. This form provides structure for consistency and transparency in reporting. For further information on Nature Portfolio policies, see our Editorial Policies and the Editorial Policy Checklist.

Statistics

For all statistical analyses, confirm that the following items are present in the figure legend, table legend, main text, or Methods section.

n/a	Confirmed	
☐	☒	The exact sample size (*n*) for each experimental group/condition, given as a discrete number and unit of measurement
☐	☒	A statement on whether measurements were taken from distinct samples or whether the same sample was measured repeatedly
☐	☒	The statistical test(s) used AND whether they are one- or two-sided<br>*Only common tests should be described solely by name; describe more complex techniques in the Methods section.*
☐	☒	A description of all covariates tested
☐	☒	A description of any assumptions or corrections, such as tests of normality and adjustment for multiple comparisons
☐	☒	A full description of the statistical parameters including central tendency (e.g. means) or other basic estimates (e.g. regression coefficient) AND variation (e.g. standard deviation) or associated estimates of uncertainty (e.g. confidence intervals)
☐	☒	For null hypothesis testing, the test statistic (e.g. *F*, *t*, *r*) with confidence intervals, effect sizes, degrees of freedom and *P* value noted<br>*Give P values as exact values whenever suitable.*
☒	☐	For Bayesian analysis, information on the choice of priors and Markov chain Monte Carlo settings
☒	☐	For hierarchical and complex designs, identification of the appropriate level for tests and full reporting of outcomes
☒	☐	Estimates of effect sizes (e.g. Cohen's *d*, Pearson's *r*), indicating how they were calculated

Our web collection on statistics for biologists contains articles on many of the points above.

Software and code

Policy information about availability of computer code

Data collection	10X Genomics Xenium Analyzer<br>Xenium ranger version 1.7.1.0<br>Xenium ranger version 2.0.0.10<br>Xenium Explorer Software (10X Genomics)<br>Olympus FLUOVIEW FV3000 confocal laser scanning microscope<br>Light microscope (model BX43, Olympus) with 10x and 20× objectives
Data analysis	RStudio 4.4.0 was used for downstream analysis of spatial transcriptomic data<br>Xenium Explorer Software was used to visualize spatial data

For manuscripts utilizing custom algorithms or software that are central to the research but not yet described in published literature, software must be made available to editors and reviewers. We strongly encourage code deposition in a community repository (e.g. GitHub). See the Nature Portfolio guidelines for submitting code & software for further information.

Data

Policy information about availability of data

All manuscripts must include a data availability statement. This statement should provide the following information, where applicable:

- Accession codes, unique identifiers, or web links for publicly available datasets
- A description of any restrictions on data availability
- For clinical datasets or third party data, please ensure that the statement adheres to our policy

Upon acceptance for publication, data will be made publicly available on the Gene Expression Omnibus (GEO) database. We will upload Xenium output data (.zip files) for each sample along with annotated cell metadata (.rds files) for reproducibility. Additionally, code will be made available upon request.

Research involving human participants, their data, or biological material

Policy information about studies with human participants or human data. See also policy information about sex, gender (identity/presentation), and sexual orientation and race, ethnicity and racism.

Reporting on sex and gender	From tissue samples collected, we have reported sex for each patient. Data is available in Supp. Table 1.
Reporting on race, ethnicity, or other socially relevant groupings	NA
Population characteristics	From each tissue sample collected, we have reported available IDH1 mutation status and sex. Data is available in Supp. Table 1.
Recruitment	N/A
Ethics oversight	All human tissue samples were collected in accordance with the Institutional Review Board of The University of Texas MD Anderson Cancer Center, Houston, and subsequently de-identified for all experimental studies. All samples were collected analyzed retrospectively via protocol 2021-1041_MOD001. This study is not associated with a clinical trial.

Note that full information on the approval of the study protocol must also be provided in the manuscript.

Field-specific reporting

Please select the one below that is the best fit for your research. If you are not sure, read the appropriate sections before making your selection.

☒ Life sciences ☐ Behavioural & social sciences ☐ Ecological, evolutionary & environmental sciences

For a reference copy of the document with all sections, see nature.com/documents/nr-reporting-summary-flat.pdf

Life sciences study design

All studies must disclose on these points even when the disclosure is negative.

Sample size	Grade II diffuse glioma (n=9), grade III astrocytoma (n=11), GBM (n=8) and non-cancerous brain tissues (n=7).
Data exclusions	No data was excluded
Replication	Gene expression validation was performed on a publicly available annotated scRNA-seq dataset. Data from Spitzer et al. 16was downloaded from Gene Expression Omnibus (GSE274546). From this dataset, primary GBM (n=59) samples were subset and used for validation of our spatial transcriptomics results.<br>Additionally, we analyzed additional brain tumor samples to validate the spatial transcriptomic data by immunohistochemistry and immunofluorescence. Additional samples used for validation included Grade II glioma (n=3), Grade III astrocytoma (n=3), GBM (n=5).
Randomization	Tumor samples were randomly selected from available tissue housed in the Brain Tumor Center/CNS Tumor Bank at The University of Texas MD Anderson Cancer Center. Collection occurred via approved IRB protocol 2021-1041_MOD001
Blinding	Blinding was not possible because of experimental setup.

Reporting for specific materials, systems and methods

We require information from authors about some types of materials, experimental systems and methods used in many studies. Here, indicate whether each material, system or method listed is relevant to your study. If you are not sure if a list item applies to your research, read the appropriate section before selecting a response.

Materials & experimental systems

n/a	Involved in the study
☐	☒ Antibodies
☒	☐ Eukaryotic cell lines
☒	☐ Palaeontology and archaeology
☒	☐ Animals and other organisms
☒	☐ Clinical data
☒	☐ Dual use research of concern
☒	☐ Plants

Methods

n/a	Involved in the study
☒	☐ ChIP-seq
☒	☐ Flow cytometry
☒	☐ MRI-based neuroimaging

Antibodies

Antibodies used

Primary antibodies:
Rabbit polyclonal anti-Annexin A1 antibody (cat# 21990-1-AP, lot#00118478, Proteintech).
Rabbit polyclonal anti-Annexin A2 antibody (cat#11256-1-AP, lot#00116068, Proteintech).
Goat polyclonal anti-Lumican antibody (cat# AF2846, lot#VPG052411A, R&D Systems).
Goat polyclonal anti-CD31(PECAM-1) antibody (cat#AF3628, lot#YZU0224011, R&D Systems).
Chicken polyclonal anti-GFAP antibody (cat#NBP1-05198, lot#7529-042324, Novus).
Rabbit polyclonal anti-Iba1 (AIF1) antibody (cat#013-27691, lot#CKP1215, Fujifilm Wako).
Mouse monoclonal anti-Fibronectin antibody (cat#66042-1-Ig, lot#10026517, Proteintech).
Rabbit monoclonal anti-IGFBP2 antibody (cat#ab188200, lot#1000635-48, Abcam).
Rabbit polyclonal anti-Brevican antibody (cat#NBP2-15616, lot#41087, Novus).
Goat polyclonal anti-Netrin-4 antibody (cat#AF1254-SP, lot#HYR022508A, R&D Systems).

Secondary antibodies:
Alexa Flour® 594 donkey anti-chicken (1:500, cat#703-585-155, lot#147524, Jackson ImmunoResearch).
Alexa Flour® 488 donkey anti-rabbit (1:500, cat#711-545-152, lot#176130, Jackson ImmunoResearch).
Alexa Fluor® 647 donkey anti-goat (1:500, cat#705-605-147, lot#168818, Jackson ImmunoResearch).
Horse anti-goat IgG (H+L), biotinylated (1:500, cat#BA-9500, lot#ZK0217, Vector Laboratories Inc.).
Horse anti-rabbit IgG (H+L), biotinylated (1:500, cat#BA-1100, lot#ZH1026, Vector Laboratories Inc.).

Validation

Annexin A1
Supplier: Proteintech
Catalog #: 21990-1-AP
Lot #: 00118478
Clone / Clonality: Rabbit polyclonal
RRID: AB_11182596
Applications used in this study: IF-paraffin
Dilution(s) used: 1:200
Manufacturer's validation data: Validated by Proteintech for WB, IHC-P, IF in human/mouse/rat. Datasheet shows WB and IHC images and recommended dilutions.

Annexin A2
Supplier: Proteintech
Catalog #: 11256-1-AP
Lot #: 00116068
Clone / Clonality: Rabbit polyclonal
RRID: AB_2057311
Applications used in this study: IF-paraffin
Dilution(s) used: 1:200.
Manufacturer's validation data: Validated by Proteintech for WB, IHC-P, IF in human/mouse/rat. Datasheet provides WB/IHC validation images and dilution recommendations.

Lumican
Supplier: R&D Systems
Catalog #: AF2846
Lot #: VPG052411A
Clone / Clonality: Goat polyclonal
RRID: AB_2136173
Applications used in this study: IHC-paraffin
Dilution(s) used: 1:500.
Manufacturer's validation data: Validated by R&D for human. Datasheet reports ELISA/WB specificity and ~10% cross-reactivity with mouse recombinant Lumican.

CD31 (PECAM-1)
Supplier: R&D Systems
Catalog #: AF3628
Lot #: YZU0224011
Clone / Clonality: Goat polyclonal

RRID: AB_2161028
Applications used in this study: IF-paraffin
Dilution(s) used: 1:100.
Manufacturer's validation data: Validated by R&D for IHC, IF, WB. Datasheet includes endothelial staining images and multiple application examples.

GFAP
Supplier: Novus
Catalog #: NBP1-05198
Lot #: 7529-042324
Clone / Clonality: Chicken polyclonal
RRID: AB_1625983
Applications used in this study: IF-paraffin
Dilution(s) used: 1:2000.
Manufacturer's validation data: Validated by Novus for WB, IHC-P, ICC/IF. Datasheet provides WB image (bands at 55 and 48 kDa) and IHC staining examples.

Iba1 (AIF1)
Supplier: Fujifilm Wako
Catalog #: 013-27691
Lot #: CKP1215
Clone / Clonality: Rabbit polyclonal
RRID: AB_839504
Applications used in this study: IF-paraffin
Dilution(s) used: 1:200.
Manufacturer's validation data: Validated by Wako for paraffin IHC and ICC. Datasheet describes antigen (C-terminal peptide) and provides IHC validation images.

Fibronectin
Supplier: Proteintech
Catalog #: 66042-1-Ig
Lot #: 10026517
Clone / Clonality: Mouse monoclonal, clone 1G10F9 (IgG1)
RRID: AB_2880736
Applications used in this study: IHC-paraffin.
Dilution(s) used: 1:300.
Manufacturer's validation data: Validated for WB, IHC, IF in human/mouse/rat. Datasheet includes clone ID, WB bands, and IHC staining images.

IGFBP2
Supplier: Abcam
Catalog #: ab188200
Lot #: 1000635-48
Clone / Clonality: Rabbit recombinant monoclonal, clone EPR18012-257
RRID: AB_2938998
Applications used in this study: IF-paraffin
Dilution(s) used: 1:200.
Manufacturer's validation data: Validated by Abcam for WB, IHC-P, IF, IP. Datasheet provides WB/IP/IHC images and flow cytometry validation.

Brevican
Supplier: Novus
Catalog #: NBP2-15616
Lot #: 41087
Clone / Clonality: Rabbit polyclonal
RRID: AB_3262996
Applications used in this study: IHC-paraffin.
Dilution(s) used: 1:500.
Manufacturer's validation data: Validated by Novus for WB, IHC, ICC. Datasheet states immunogen (N-terminal human brevican) and provides validation images.

Netrin-4
Supplier: R&D Systems
Catalog #: AF1254-SP
Lot #: HYR022508A
Clone / Clonality: Goat polyclonal
RRID: AB_2296234
Applications used in this study: IF-paraffin.
Dilution(s) used: 1:200.
Manufacturer's validation data: Validated by R&D for WB, IHC, ELISA. Datasheet notes ~50% cross-reactivity to mouse recombinant NTN4, with specific WB/IHC examples.

Donkey anti-chicken IgY (Alexa Fluor 594)
Supplier: Jackson ImmunoResearch
Catalog #: 703-585-155
Lot #: Specify

Clone / Clonality: Donkey polyclonal, cross-adsorbed
RRID: AB_2340376
Applications used in this study: IF-paraffin
Dilution(s) used: 1:500.
Manufacturer's validation data: Affinity purified, cross-adsorbed to minimize species cross-reactivity. Manufacturer datasheet confirms use for IF with Alexa Fluor 594 label.

Donkey anti-rabbit IgG (Alexa Fluor 488)
Supplier: Jackson ImmunoResearch
Catalog #: 711-545-152
Lot #: Specify
Clone / Clonality: Donkey polyclonal, cross-adsorbed
RRID: AB_2313584
Applications used in this study: IF-paraffin
Dilution(s) used: 1:500
Manufacturer's validation data: Affinity purified, cross-adsorbed. Validated by manufacturer for IF; datasheet includes fluorophore details and tested species reactivity.

Donkey anti-goat IgG (Alexa Fluor 647)
Supplier: Jackson ImmunoResearch
Catalog #: 705-605-147
Lot #: Specify
Clone / Clonality: Donkey polyclonal, cross-adsorbed
RRID: AB_2340437
Applications used in this study: IF-paraffin
Dilution(s) used: 1:500.
Manufacturer's validation data: Manufacturer validated for IF; cross-adsorbed to minimize cross-reactivity; datasheet includes spectral properties.
Horse anti-goat IgG (biotinylated)
Supplier: Vector Laboratories
Catalog #: BA-9500
Lot #: Specify
Clone / Clonality: Horse polyclonal
RRID: AB_2336123
Applications used in this study: IHC-paraffin.
Dilution(s) used: 1:500.
Manufacturer's validation data: Validated by Vector for IHC and immunoblotting. Datasheet includes recommended dilutions and specificity for goat IgG.

Horse anti-rabbit IgG (biotinylated)
Supplier: Vector Laboratories
Catalog #: BA-1100
Lot #: Specify
Clone / Clonality: Horse polyclonal
RRID: AB_2336201
Applications used in this study: IHC-paraffin.
Dilution(s) used: 1:500.
Manufacturer's validation data: Validated by Vector for IHC and immunoblotting. Manufacturer datasheet includes staining examples and dilution ranges.

Plants

Seed stocks

NA

Novel plant genotypes

NA

Authentication

NA
